# Supplementary material for: Characterization of Alternative Splicing in High-Risk Wilms’ Tumors
Source: Int J Mol Sci. 2024 Apr 20;25(8):4520. doi: 10.3390/ijms25084520 (PMC11050615; doi:10.3390/ijms25084520)
Supplement: Supplementary file 1 [file ijms-25-04520-s001.zip › Supplementary information.combined.pdf]

Supplementary information for:

Characterization of alternative splicing in high-risk Wilms' tumors

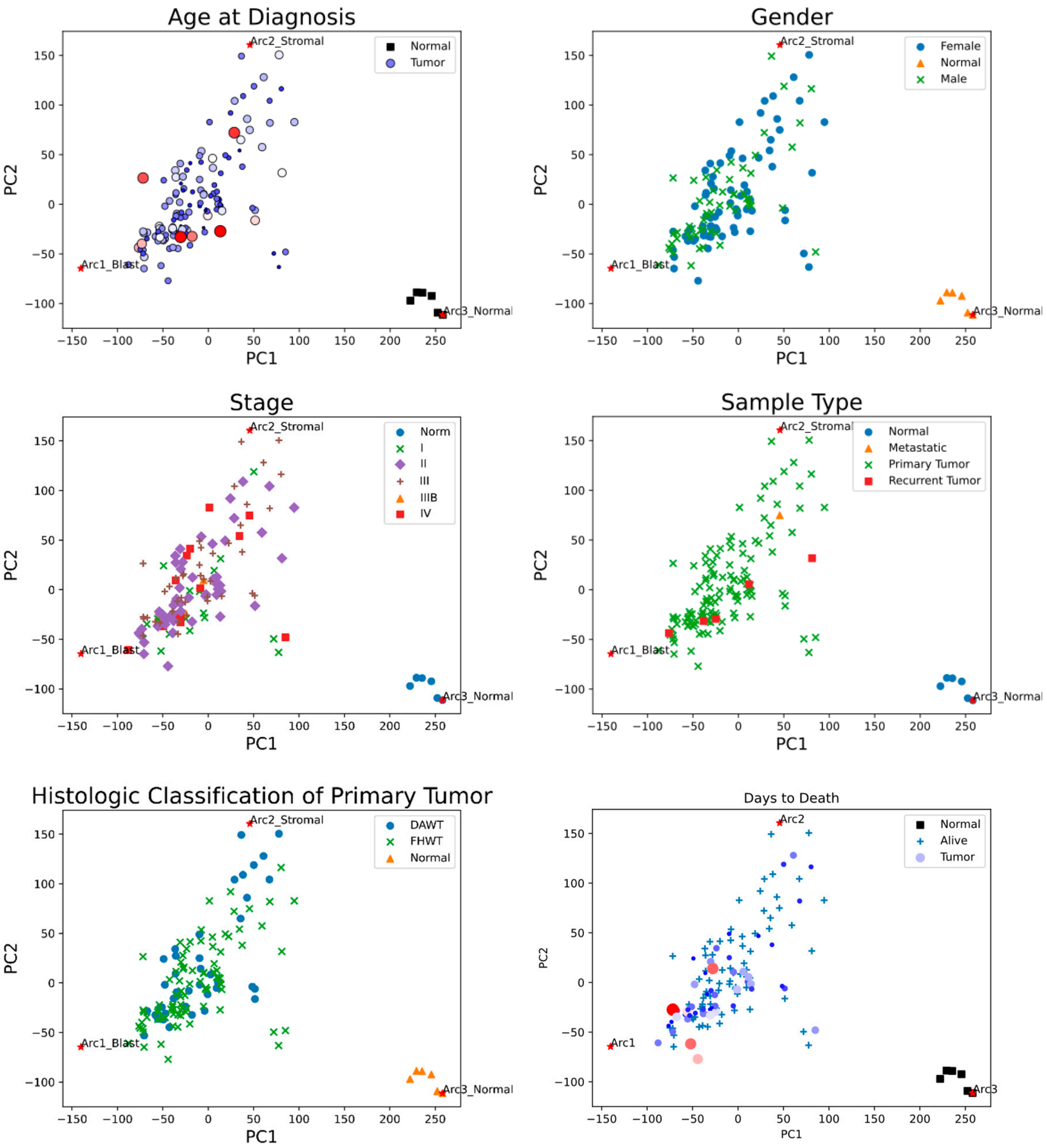

Figure S1: Patient clinical data.

Shown are PCA plots of tumors and normal kidney samples, where each sample is marked according to reported patient clinical data (age and days to death: large red – high, small blue – low).

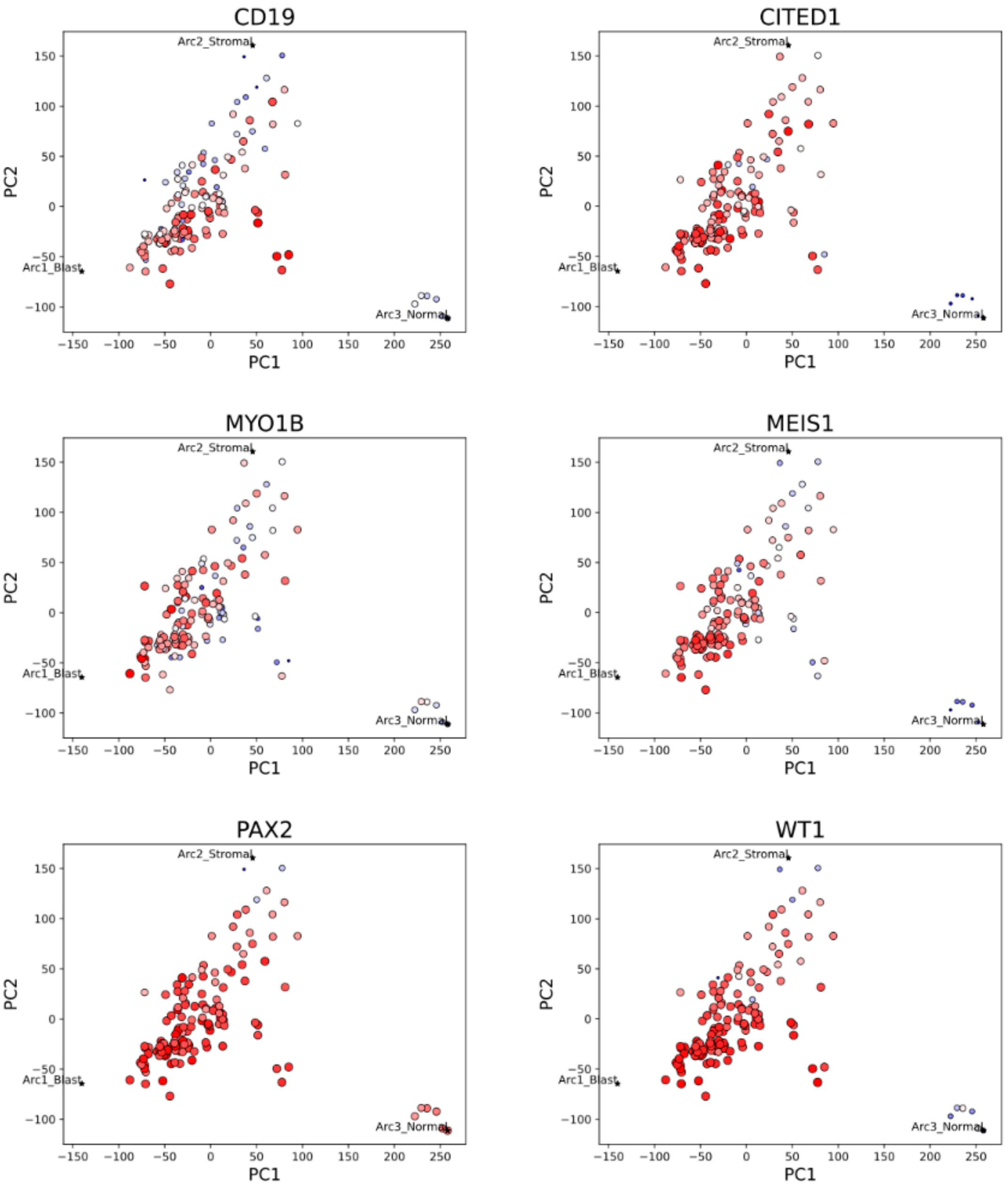

Figure S2: Genes over-expressed near the blastemal archetype.

Shown are PCA plots of tumors and normal kidney samples, where each sample is marked according to the expression levels of selected genes (large red – high, small blue – low).

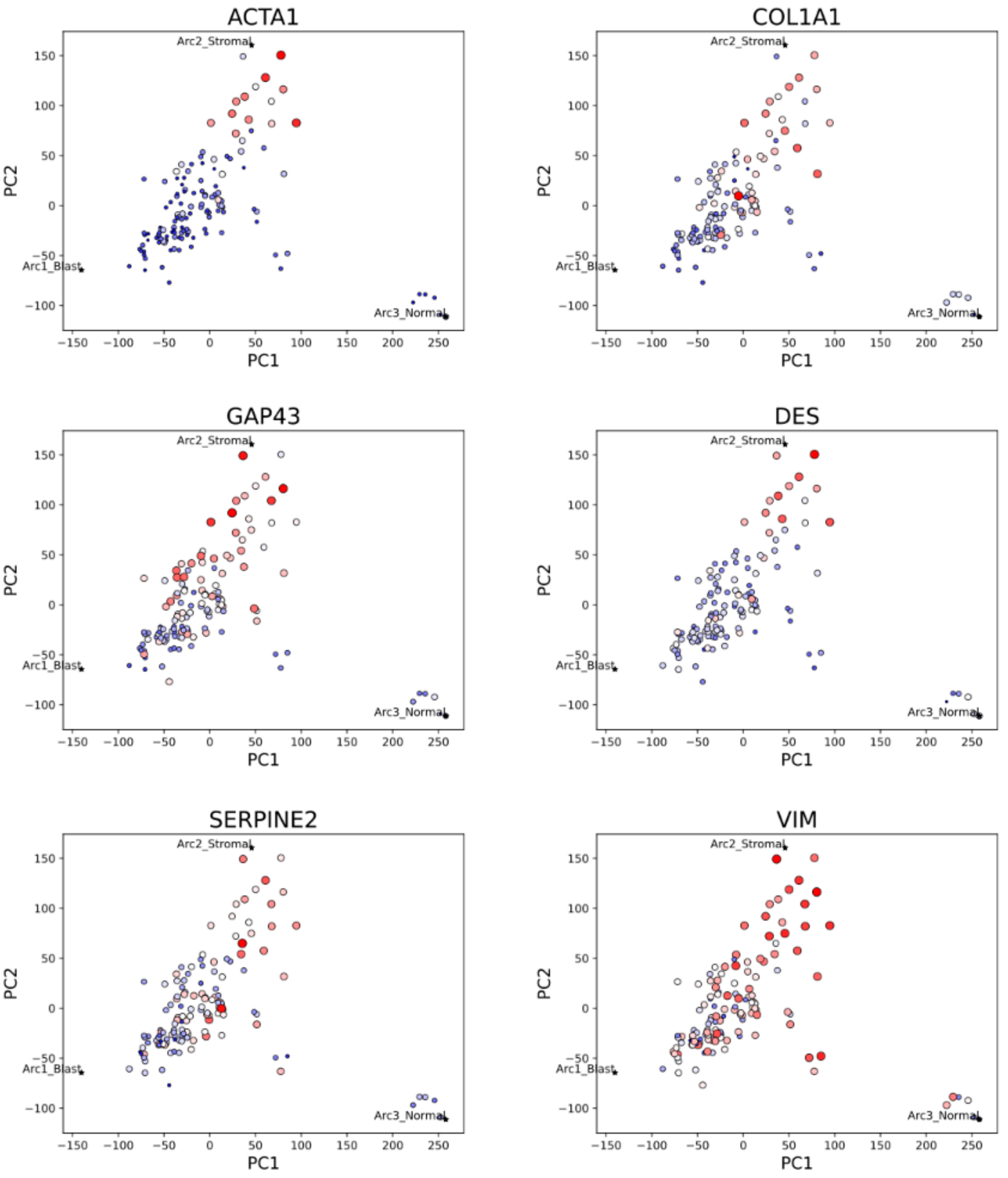

Figure S3: Genes over-expressed near the stromal archetype.

Shown are PCA plots of tumors and normal kidney samples, where each sample is marked according to the expression levels of selected genes (large red – high, small blue – low).

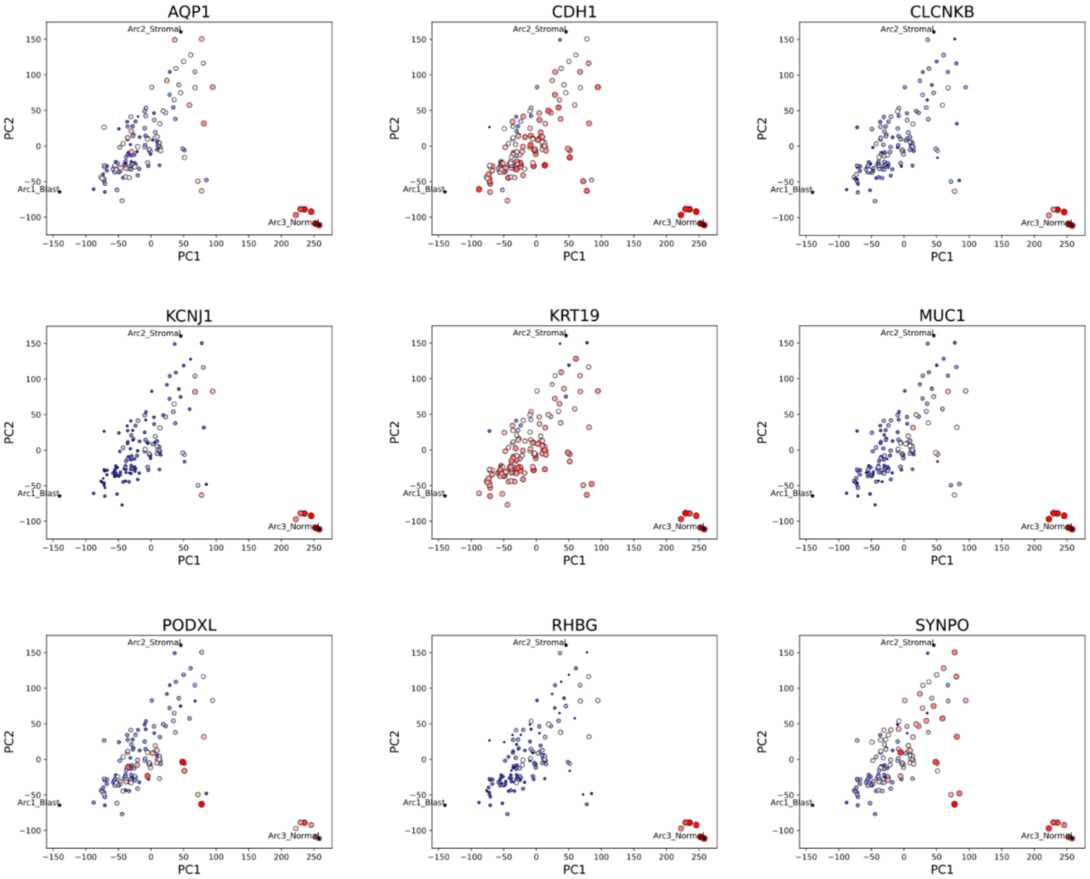

Figure S4: Genes over-expressed near the normal archetype.

Shown are PCA plots of tumors and normal kidney samples, where each sample is marked according to the expression levels of selected genes (large red – high, small blue – low).

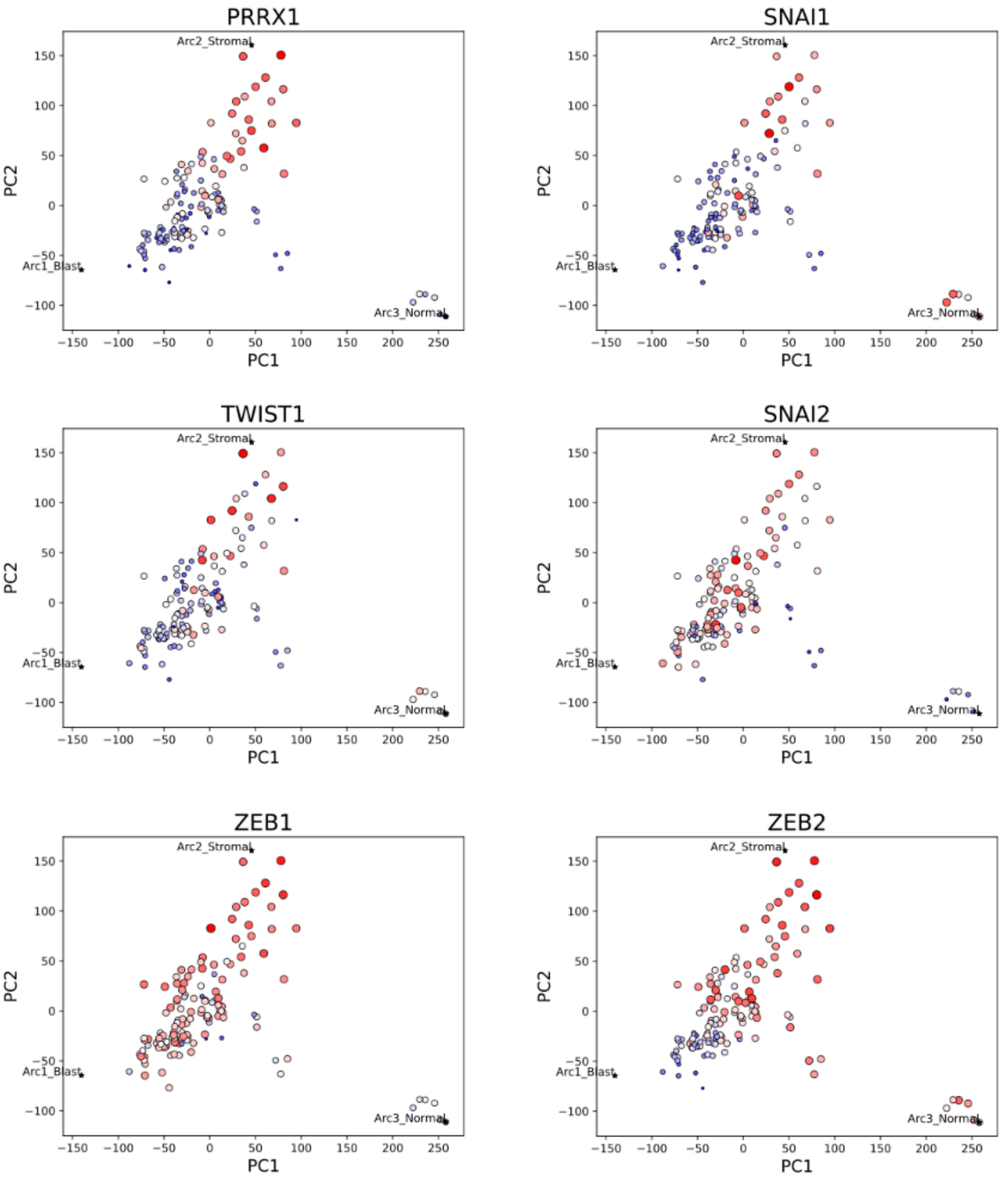

Figure S5: Transcription factors involved in the epithelial to mesenchymal transition (EMT).

Shown are PCA plots of tumors and normal kidney samples, where each sample is marked according to the expression levels of selected genes (large red – high, small blue – low).

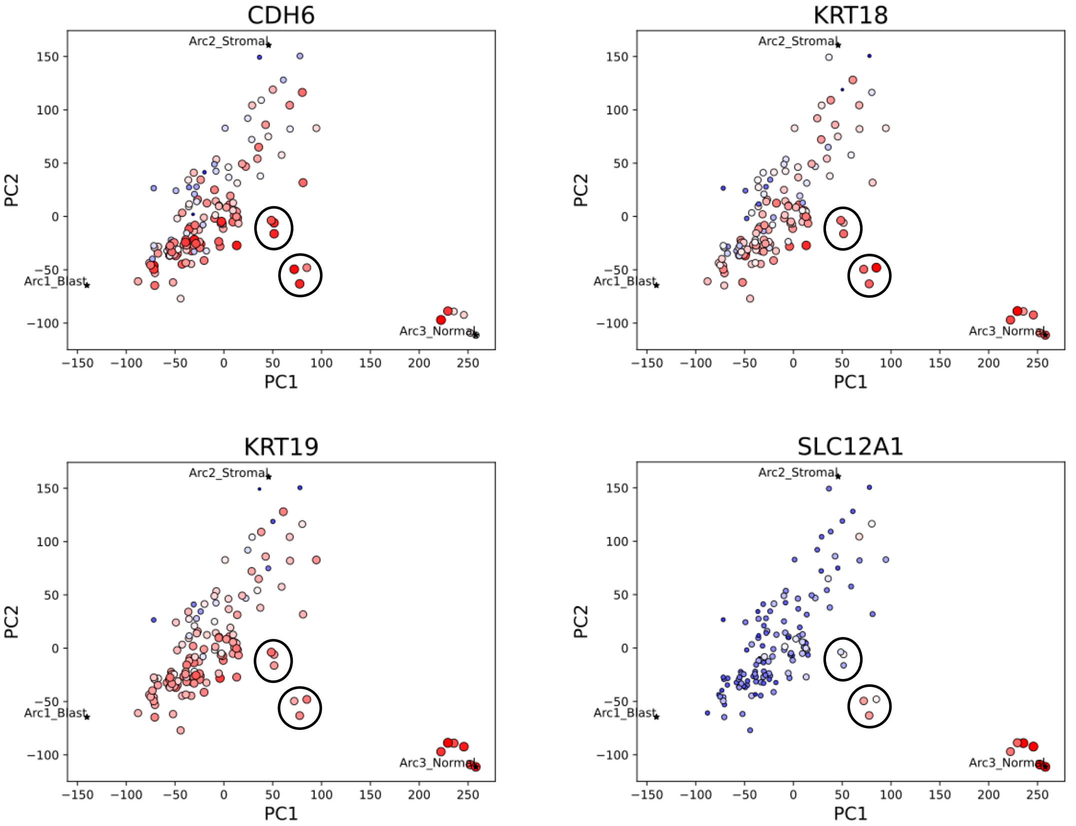

Figure S6: The six “epithelial-like” tumors express some renal epithelial associated markers.

Shown are PCA plots of tumors and normal kidney samples, where each sample is marked according to the expression levels of selected genes (large red – high, small blue – low).

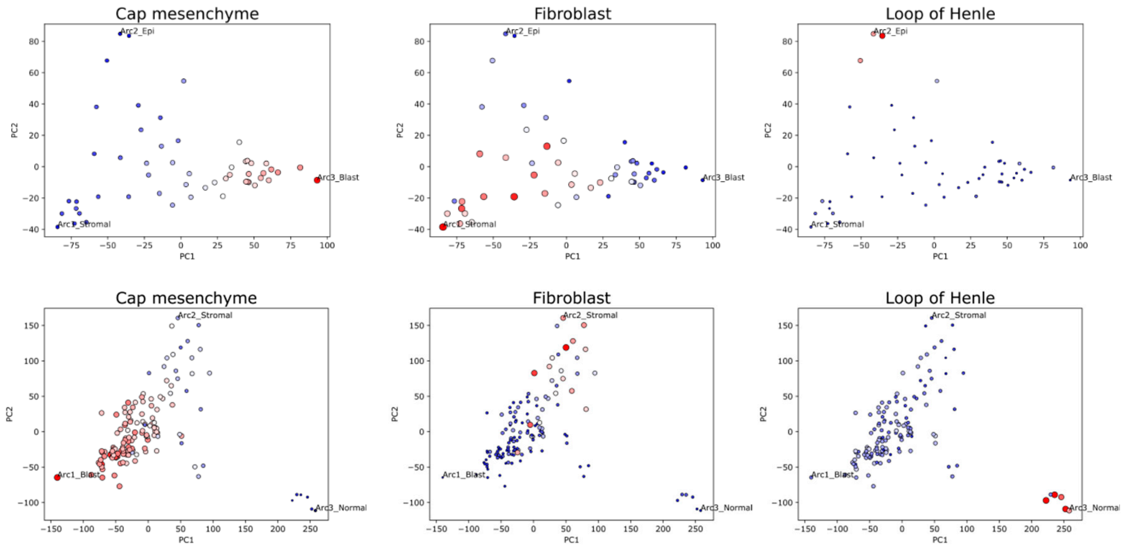

Figure S7: A comparison of cell deconvolution between two datasets of high-risk Wilms' tumors – part I.

We performed cell deconvolution and compared the inferred cell composition between two Wilms' tumor datasets: (i) Top: a dataset published by Wegert et al. [1], containing gene expression microarray measurements from high-risk Wilms' tumors that were treated with chemotherapy prior to surgery according to the *SIOP* protocol, but still contained a significant amount of remaining viable blastema (ii) Bottom: the dataset that we used in this manuscript, published by Gadd et al. [2] and the NCI TARGET initiative. This dataset contains RNAseq measurements from high-risk Wilms' tumors, that is, favorable histology Wilms' tumors (FHWT) that relapsed and diffuse anaplastic Wilms' tumors (DAWT), both treated according to the *COG* protocol and published along with associated normal kidney samples. The inferred cell composition was similar for both datasets, with the exception that in the Wegert et al. dataset, tumors near the stromal archetype were found to contain a large fraction of macrophages with respect to most other tumors. Shown are PCA plots of tumors and normal kidney samples, where each sample is marked according to the predicted proportion of a given cell type (large red – high, small blue – low).

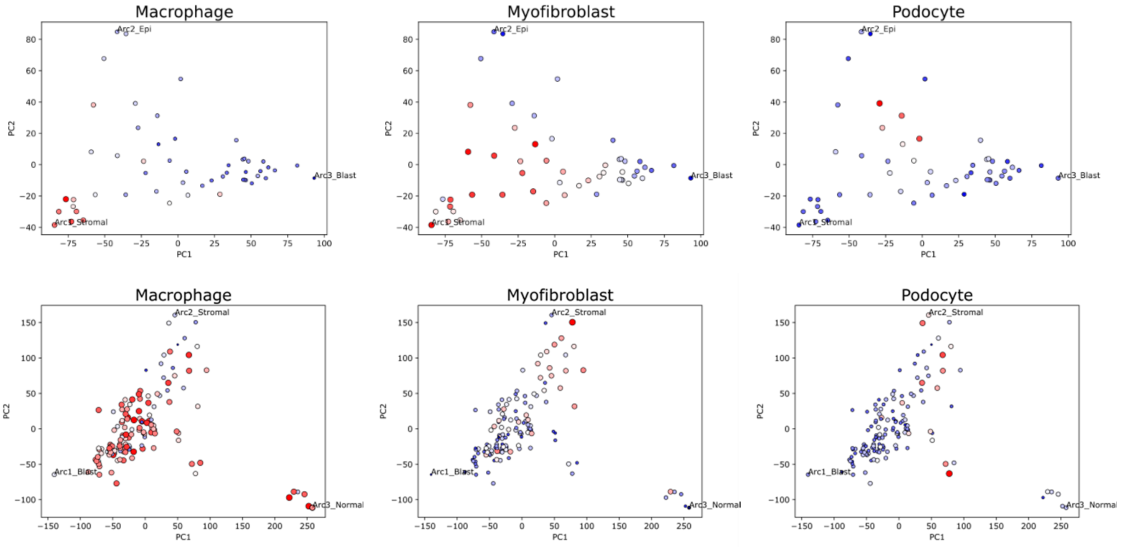

Figure S8: A comparison of cell deconvolution between two datasets of high-risk Wilms' tumors – part II.

We performed cell deconvolution and compared the inferred cell composition between two Wilms' tumor datasets: (i) Top: a dataset published by Wegert et al. [1], containing gene expression microarray measurements from high-risk Wilms' tumors that were treated with chemotherapy prior to surgery according to the *SIOP* protocol, but still contained a significant amount of remaining viable blastema (ii) Bottom: the dataset that we used in this manuscript, published by Gadd et al. [2] and the NCI TARGET initiative. This dataset contains RNAseq measurements from high-risk Wilms' tumors, that is, favorable histology Wilms' tumors (FHWT) that relapsed and diffuse anaplastic Wilms' tumors (DAWT), both treated according to the *COG* protocol and published along with associated normal kidney samples. The inferred cell composition was similar for both datasets, with the exception that in the Wegert et al. dataset, tumors near the stromal archetype were found to contain a large fraction of macrophages with respect to most other tumors. Shown are PCA plots of tumors and normal kidney samples, where each sample is marked according to the predicted proportion of a given cell type (large red – high, small blue – low).

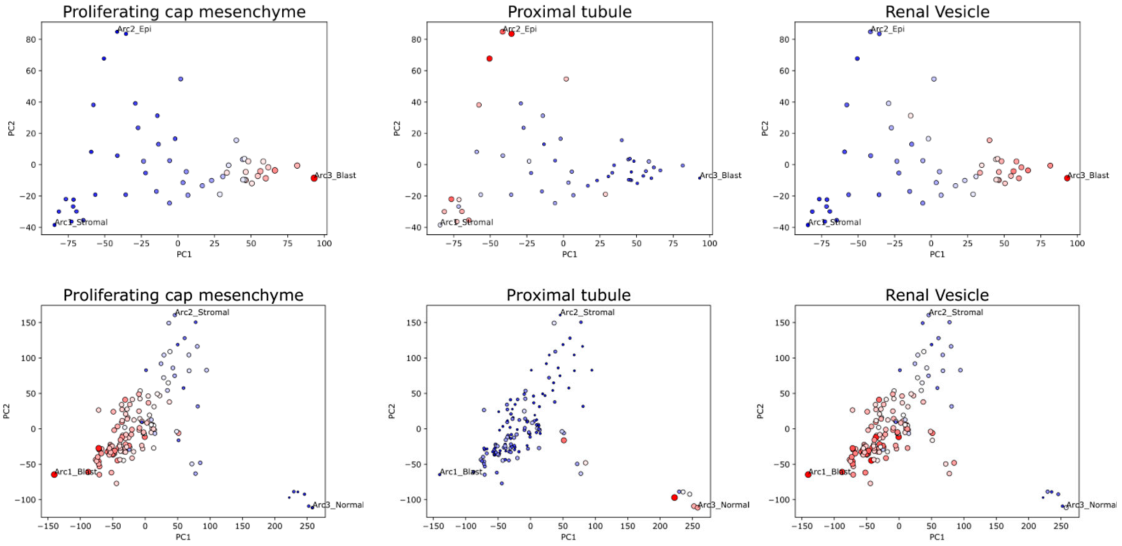

Figure S9: A comparison of cell deconvolution between two datasets of high-risk Wilms' tumors – part III.

We performed cell deconvolution and compared the inferred cell composition between two Wilms' tumor datasets: (i) Top: a dataset published by Wegert et al. [1], containing gene expression microarray measurements from high-risk Wilms' tumors that were treated with chemotherapy prior to surgery according to the *SIOP* protocol, but still contained a significant amount of remaining viable blastema (ii) Bottom: the dataset that we used in this manuscript, published by Gadd et al. [2] and the NCI TARGET initiative. This dataset contains RNAseq measurements from high-risk Wilms' tumors, that is, favorable histology Wilms' tumors (FHWT) that relapsed and diffuse anaplastic Wilms' tumors (DAWT), both treated according to the *COG* protocol and published along with associated normal kidney samples. The inferred cell composition was similar for both datasets, with the exception that in the Wegert et al. dataset, tumors near the stromal archetype were found to contain a large fraction of macrophages with respect to most other tumors. Shown are PCA plots of tumors and normal kidney samples, where each sample is marked according to the predicted proportion of a given cell type (large red – high, small blue – low).

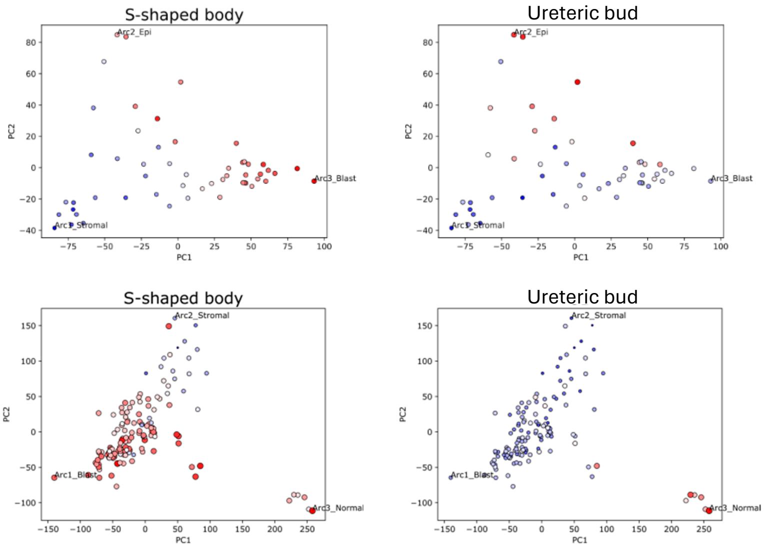

Figure S10: A comparison of cell deconvolution between two datasets of high-risk Wilms' tumors – part IV.

We performed cell deconvolution and compared the inferred cell composition between two Wilms' tumor datasets: (i) Top: a dataset published by Wegert et al. [1], containing gene expression microarray measurements from high-risk Wilms' tumors that were treated with chemotherapy prior to surgery according to the *SIOP* protocol, but still contained a significant amount of remaining viable blastema (ii) Bottom: the dataset that we used in this manuscript, published by Gadd et al. [2] and the NCI TARGET initiative. This dataset contains RNAseq measurements from high-risk Wilms' tumors, that is, favorable histology Wilms' tumors (FHWT) that relapsed and diffuse anaplastic Wilms' tumors (DAWT), both treated according to the *COG* protocol and published along with associated normal kidney samples. The inferred cell composition was similar for both datasets, with the exception that in the Wegert et al. dataset, tumors near the stromal archetype were found to contain a large fraction of macrophages with respect to most other tumors. Shown are PCA plots of tumors and normal kidney samples, where each sample is marked according to the predicted proportion of a given cell type (large red – high, small blue – low).

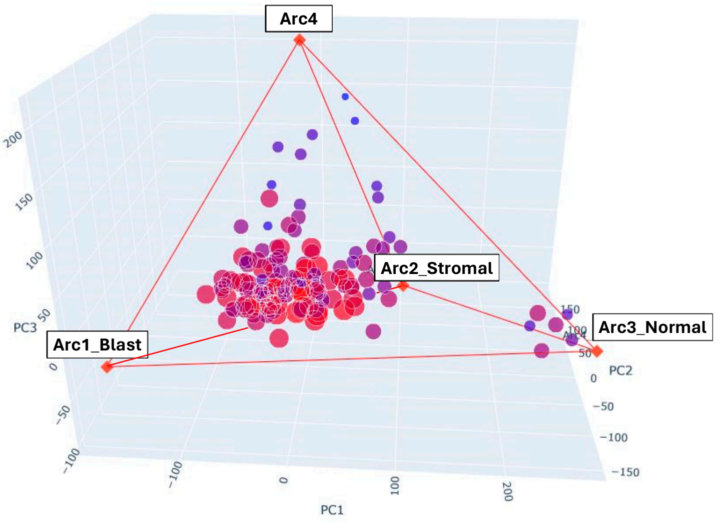

Figure S11: A fourth archetype highlights tumor samples with smaller RNAseq library size.

Shown is a 3-dimensional PCA plot of tumors and normal kidney samples, where each sample is marked according to its DESeq2 size factor (large red – high, small blue – low).

Alternative splicing analysis and additional examples for alternatively spliced transcripts:

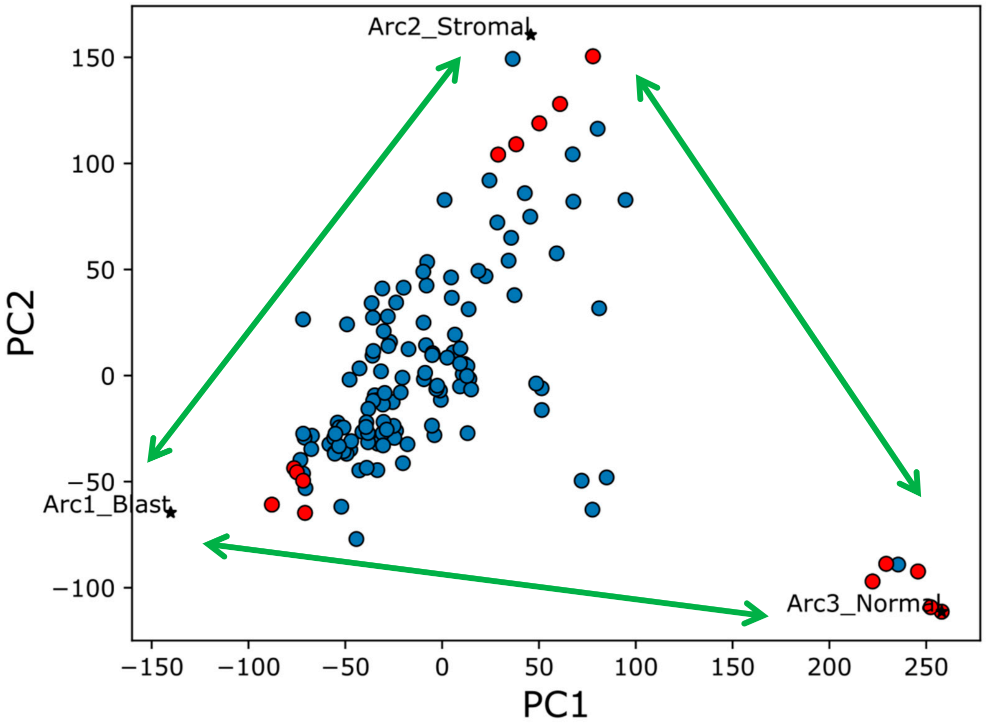

Figure S12: The five samples near the archetypes that were chosen for the three comparisons with rMAPS.

The chosen samples are marked in red, comparisons are marked with green arrows.

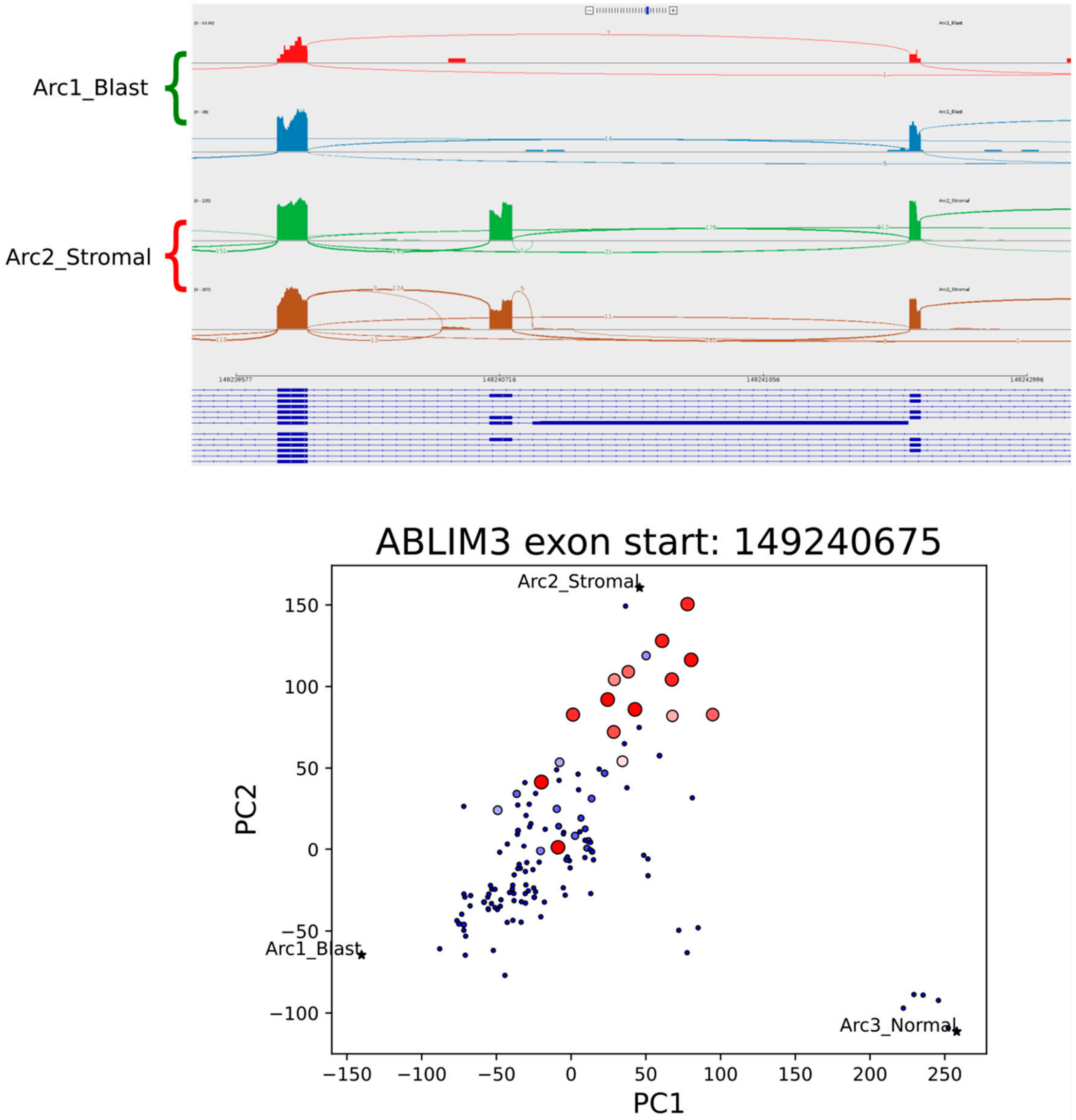

Figure S13: The gene ABLIM3 is alternatively spliced between samples located in different regions of latent space.

Top: A Shashimi plot of representative samples located near different archetypes. Bottom: a PCA plot of tumors and normal kidney samples, where each sample is marked according to the inclusion level of a selected mRNA isoform (large red – high, small blue – low).

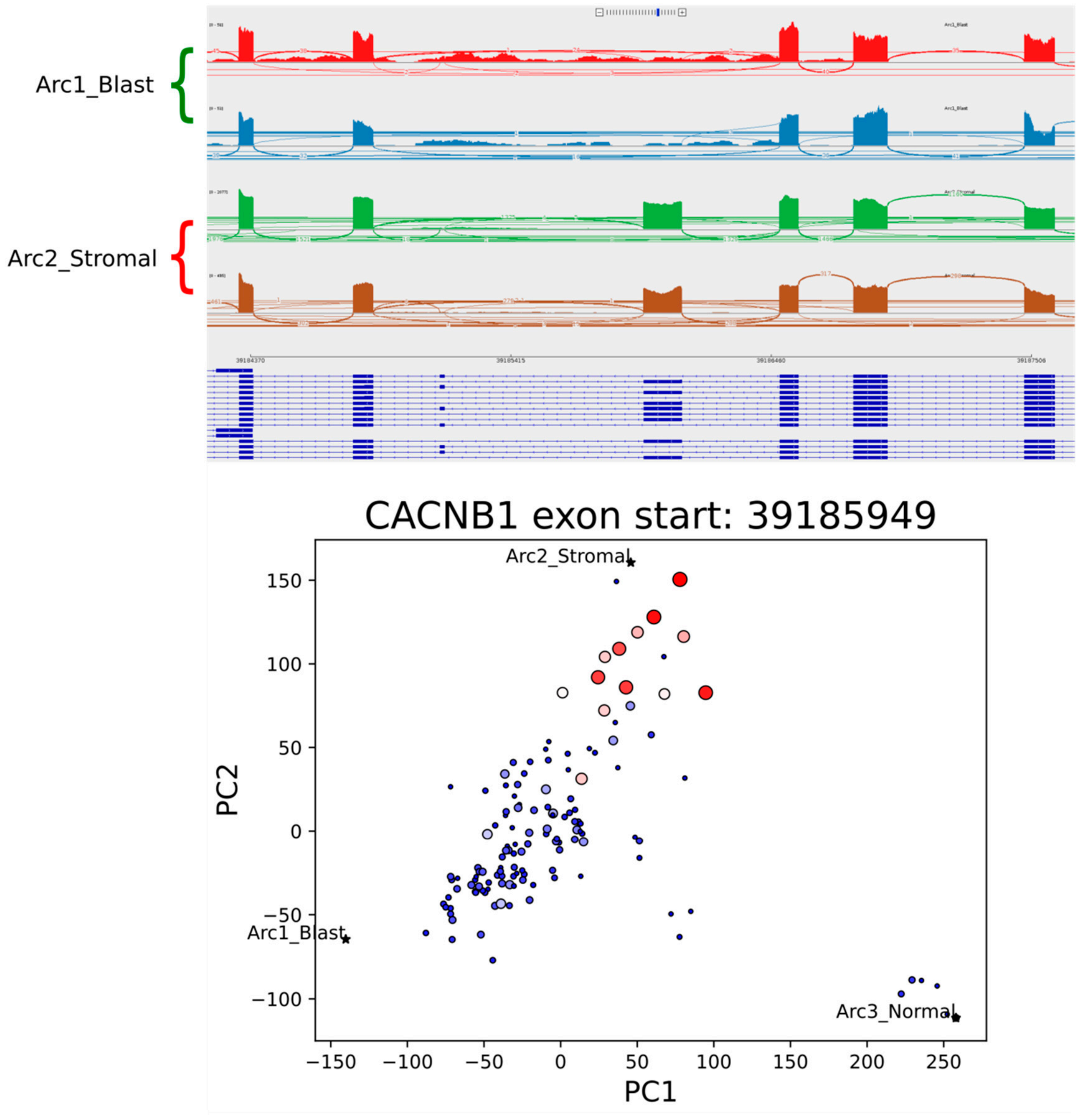

Figure S14: The gene CACNB1 is alternatively spliced between samples located in different regions of latent space.

Top: A Shashimi plot of representative samples located near different archetypes. Bottom: a PCA plot of tumors and normal kidney samples, where each sample is marked according to the inclusion level of a selected mRNA isoform (large red – high, small blue – low).

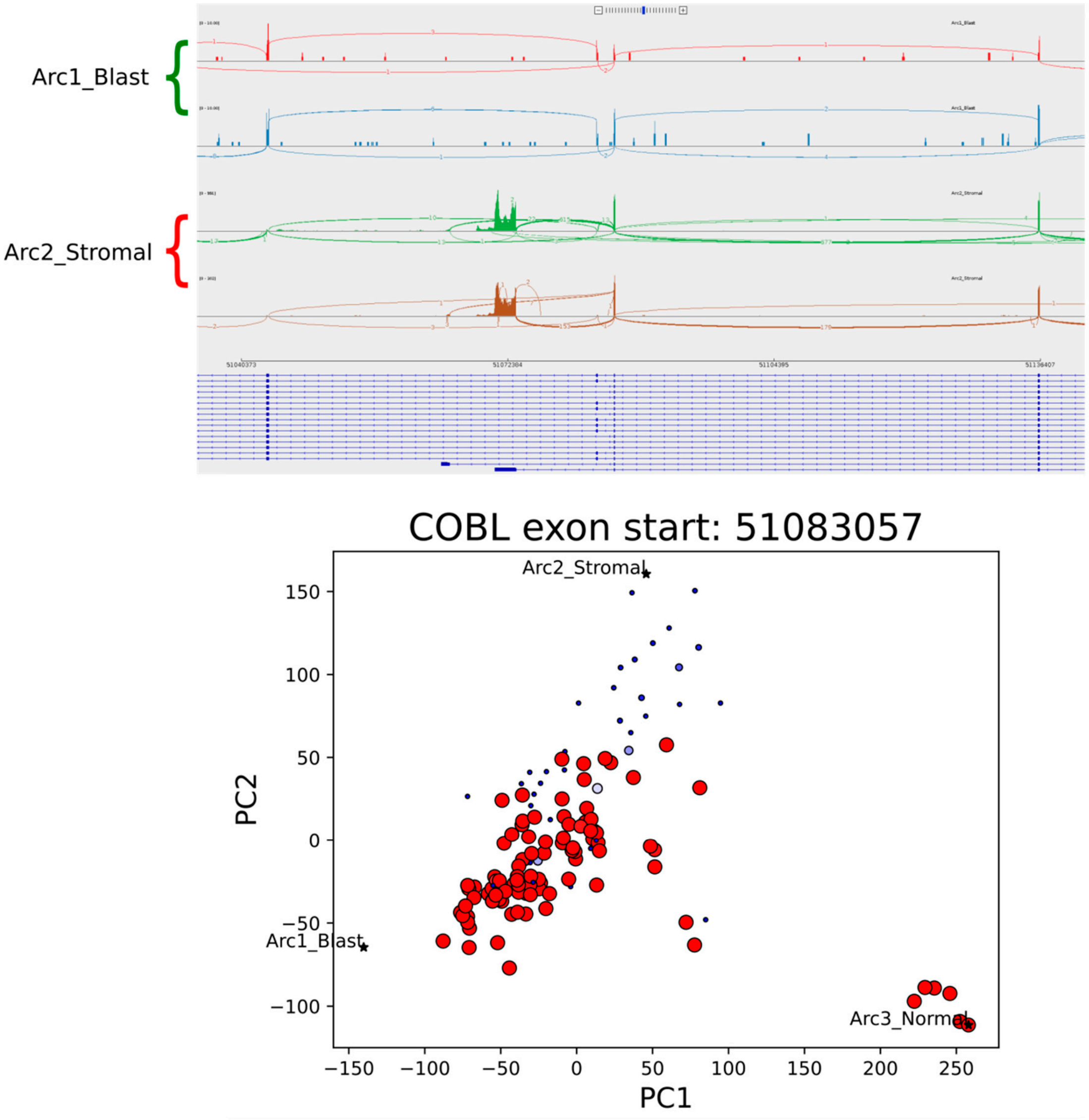

Figure S15: The gene COBL is alternatively spliced between samples located in different regions of latent space.

Top: A Shashimi plot of representative samples located near different archetypes. Bottom: a PCA plot of tumors and normal kidney samples, where each sample is marked according to the inclusion level of a selected mRNA isoform (large red – high, small blue – low).

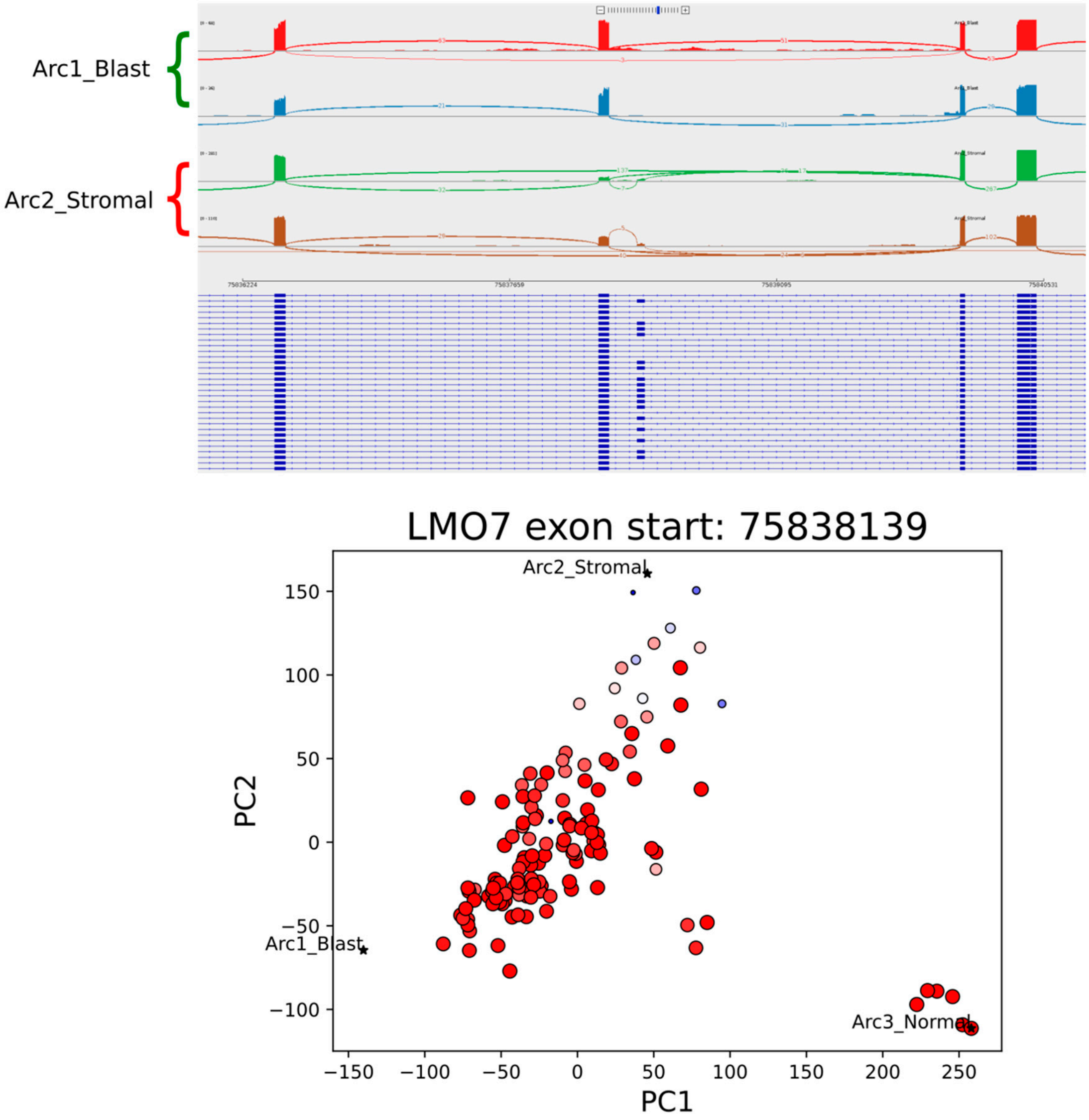

Figure S16: The gene LMO7 is alternatively spliced between samples located in different regions of latent space.

Top: A Shashimi plot of representative samples located near different archetypes. Bottom: a PCA plot of tumors and normal kidney samples, where each sample is marked according to the inclusion level of a selected mRNA isoform (large red – high, small blue – low).

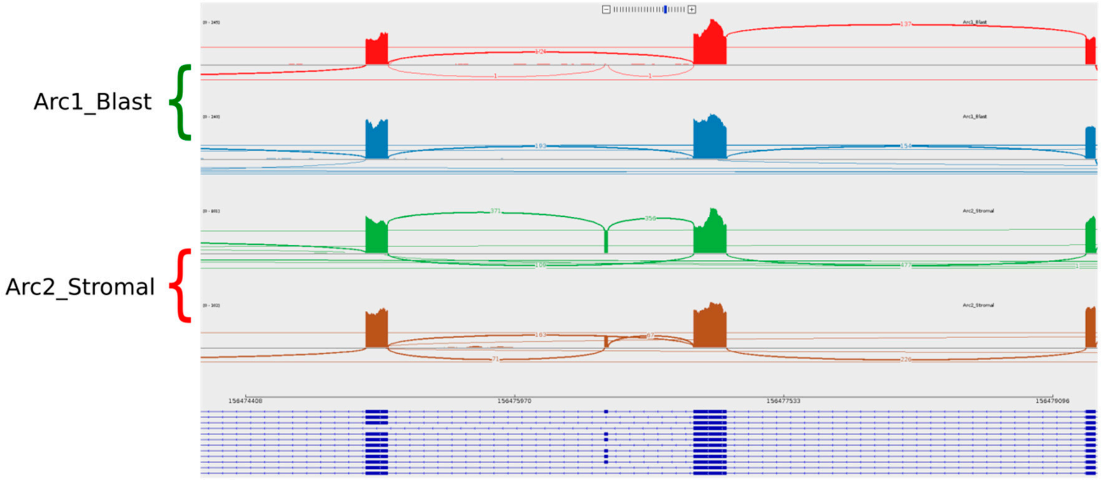

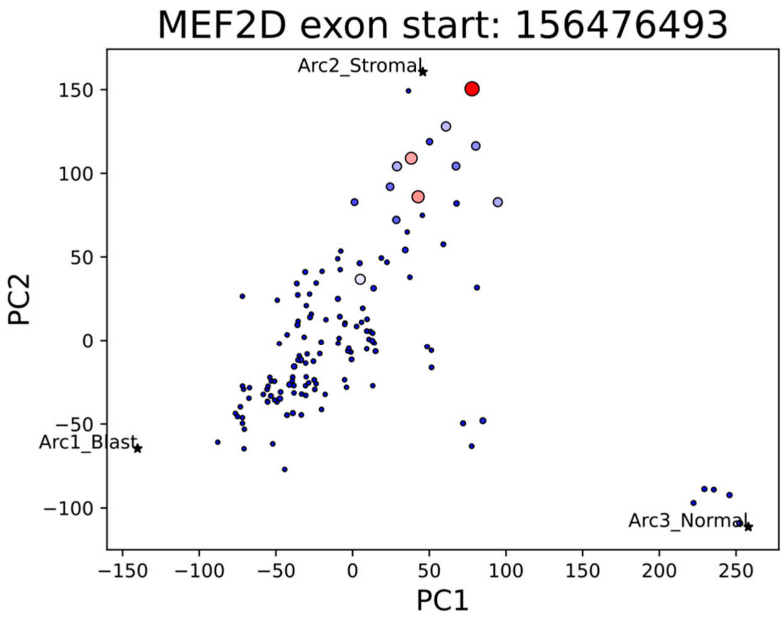

Figure S17: The gene MEF2D is alternatively spliced between samples located in different regions of latent space.

Top: A Shashimi plot of representative samples located near different archetypes. Bottom: a PCA plot of tumors and normal kidney samples, where each sample is marked according to the inclusion level of a selected mRNA isoform (large red – high, small blue – low). The cassette exon shown here is the muscle-specific “beta” exon, whose inclusion was previously found to be dependent on the splicing regulator RBFOX2 binding downstream, and also found to be required for myoblast fusion (an essential step of muscle differentiation) in the C2C12 mouse myoblast cell line [3].

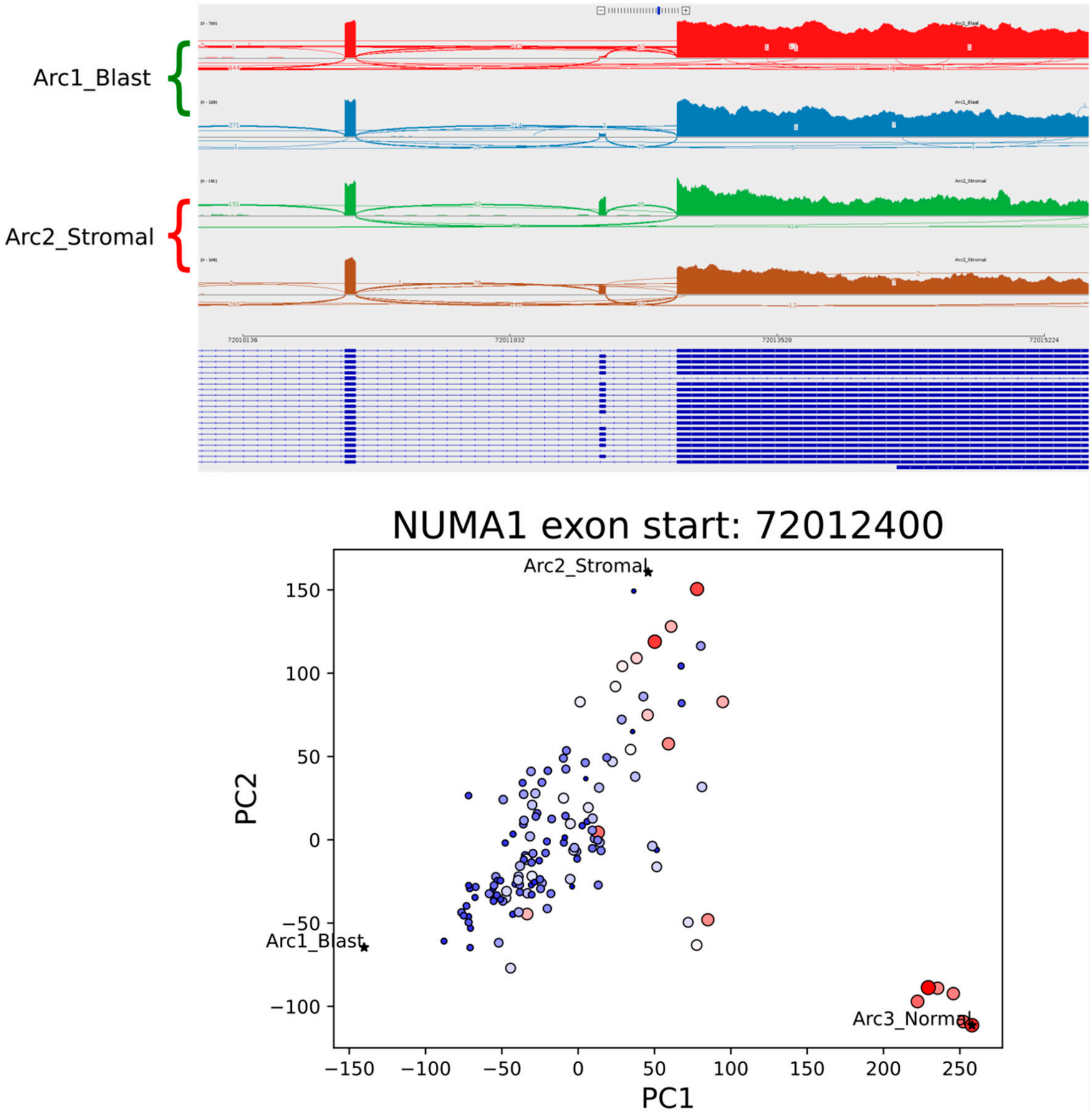

Figure S18: The gene NUMA1 is alternatively spliced between samples located in different regions of latent space.

Top: A Shashimi plot of representative samples located near different archetypes. Bottom: a PCA plot of tumors and normal kidney samples, where each sample is marked according to the inclusion level of a selected mRNA isoform (large red – high, small blue – low). The skipping of NUMA1 exon 16 in the more blastemal tumors is consistent with previous observations that depleting the expression of the splicing regulator MBNL1 induced skipping of exon 16 in NUMA1 [4].

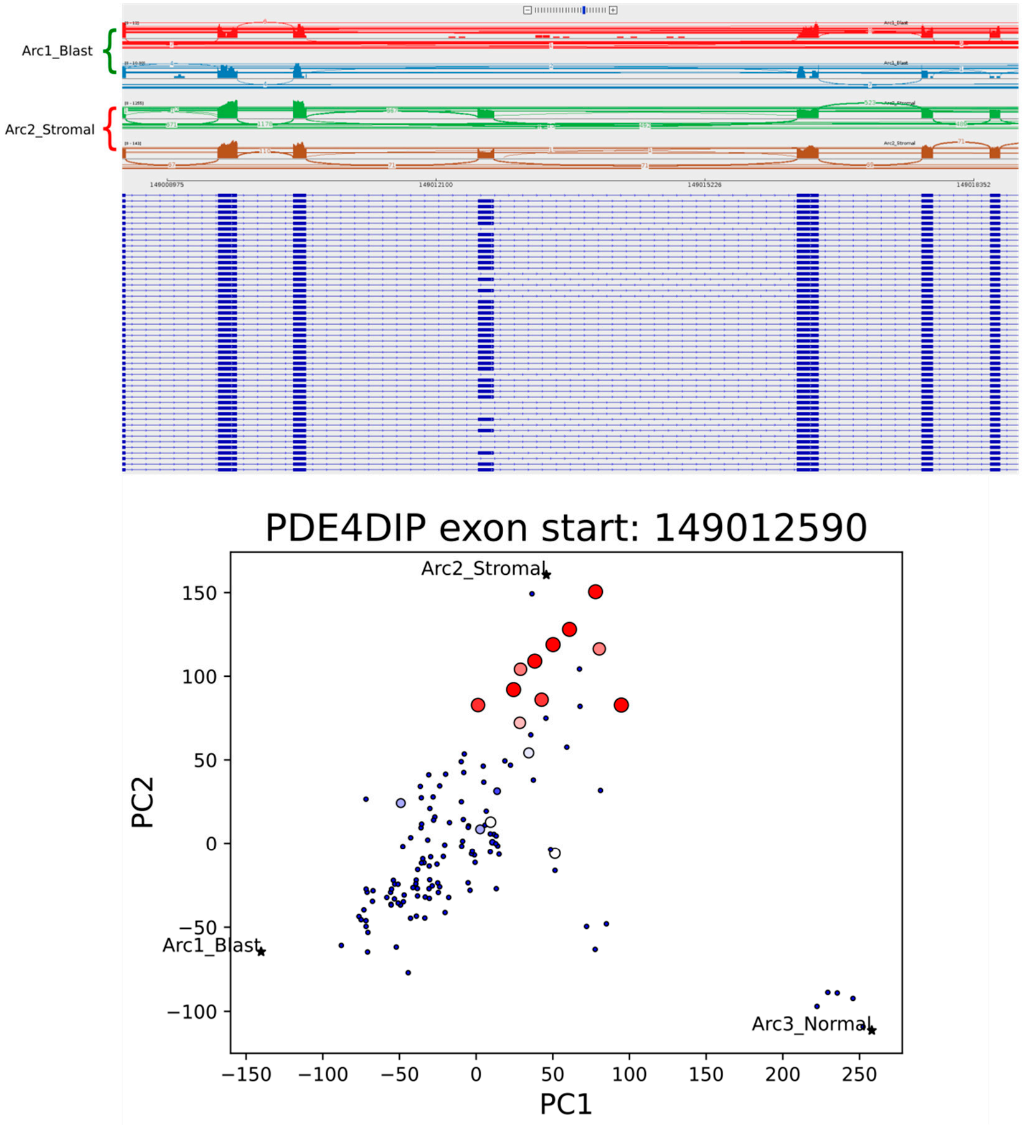

Figure S19: The gene PDE4DIP is alternatively spliced between samples located in different regions of latent space.

Top: A Shashimi plot of representative samples located near different archetypes. Bottom: a PCA plot of tumors and normal kidney samples, where each sample is marked according to the inclusion level of a selected mRNA isoform (large red – high, small blue – low).

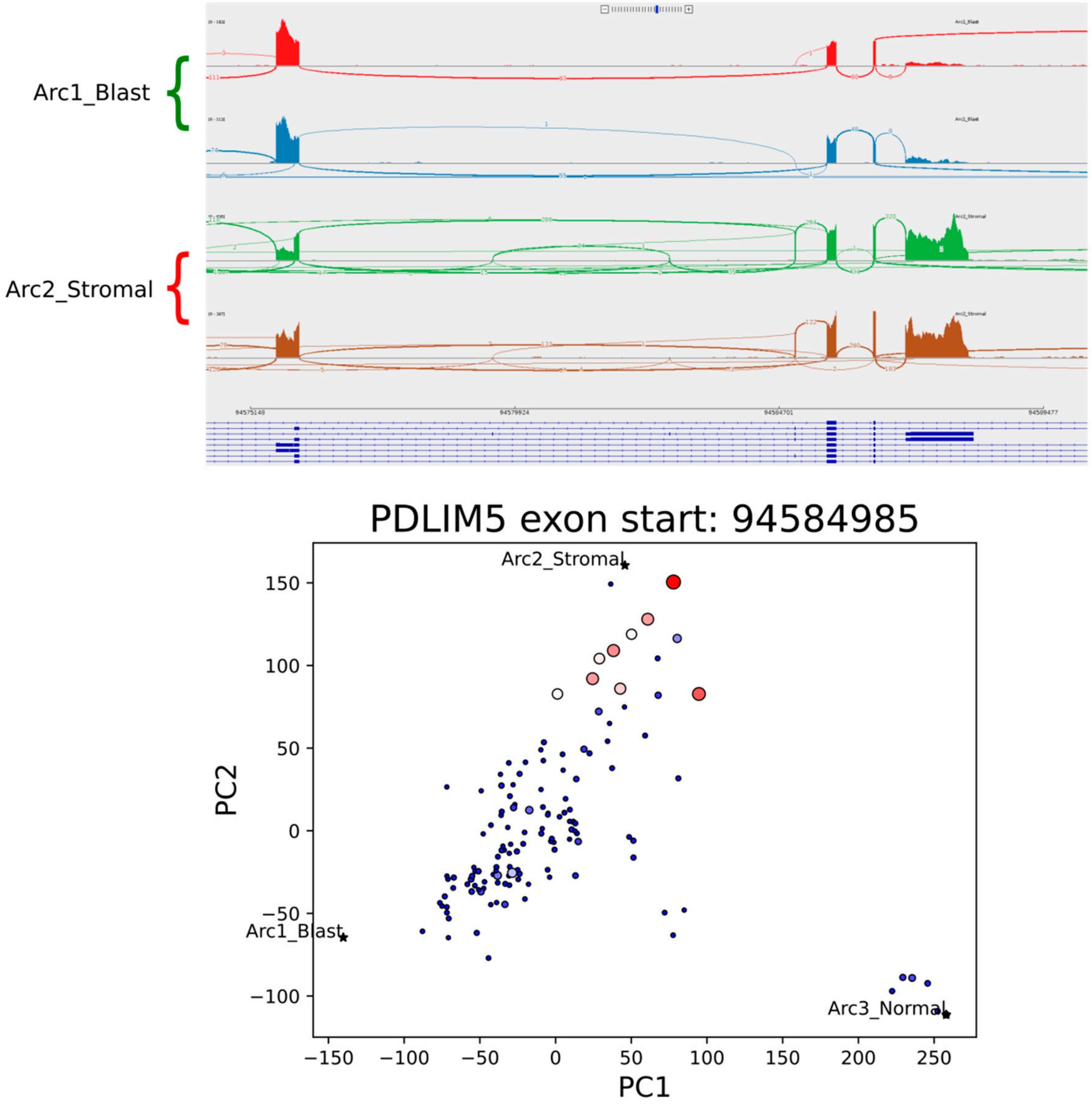

Figure S20: The gene PDLIM5 is alternatively spliced between samples located in different regions of latent space.

Top: A Shashimi plot of representative samples located near different archetypes. Bottom: a PCA plot of tumors and normal kidney samples, where each sample is marked according to the inclusion level of a selected mRNA isoform (large red – high, small blue – low).

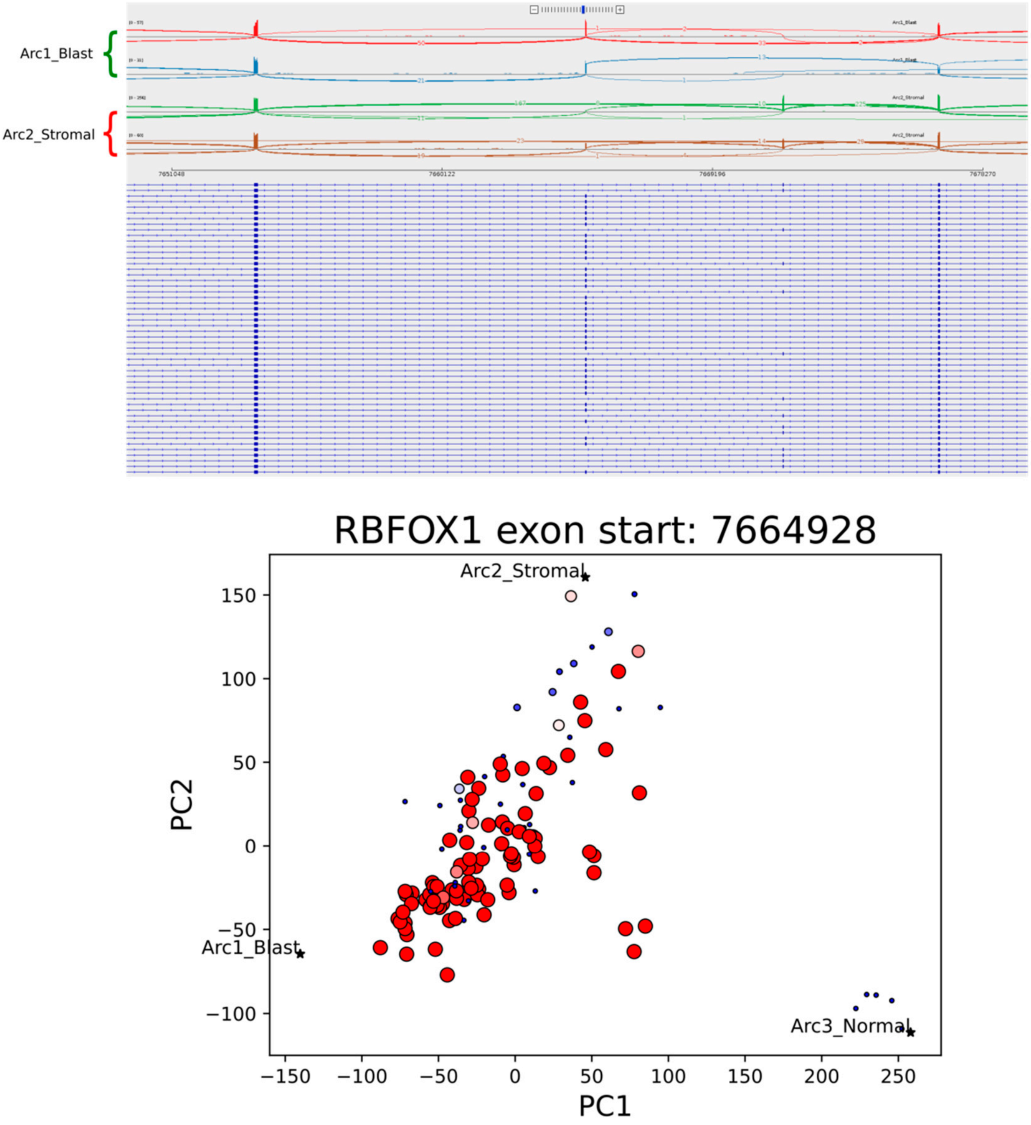

Figure S21: The gene RBFOX1 is alternatively spliced between samples located in different regions of latent space.

Top: A Shashimi plot of representative samples located near different archetypes. Bottom: a PCA plot of tumors and normal kidney samples, where each sample is marked according to the inclusion level of a selected mRNA isoform (large red – high, small blue – low).

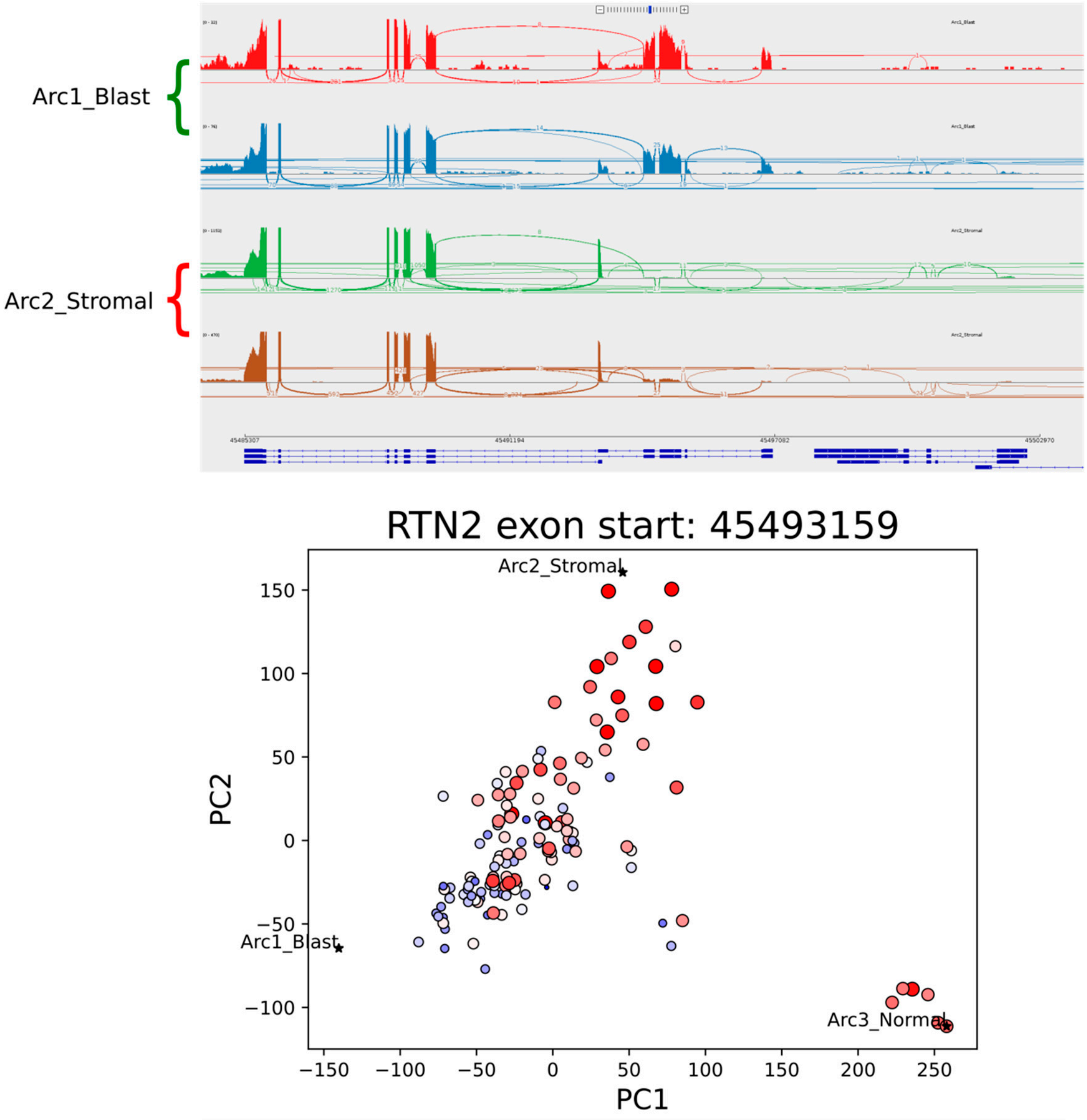

Figure S22: The gene RTN2 is alternatively spliced between samples located in different regions of latent space.

Top: A Shashimi plot of representative samples located near different archetypes. Bottom: a PCA plot of tumors and normal kidney samples, where each sample is marked according to the inclusion level of a selected mRNA isoform (large red – high, small blue – low).

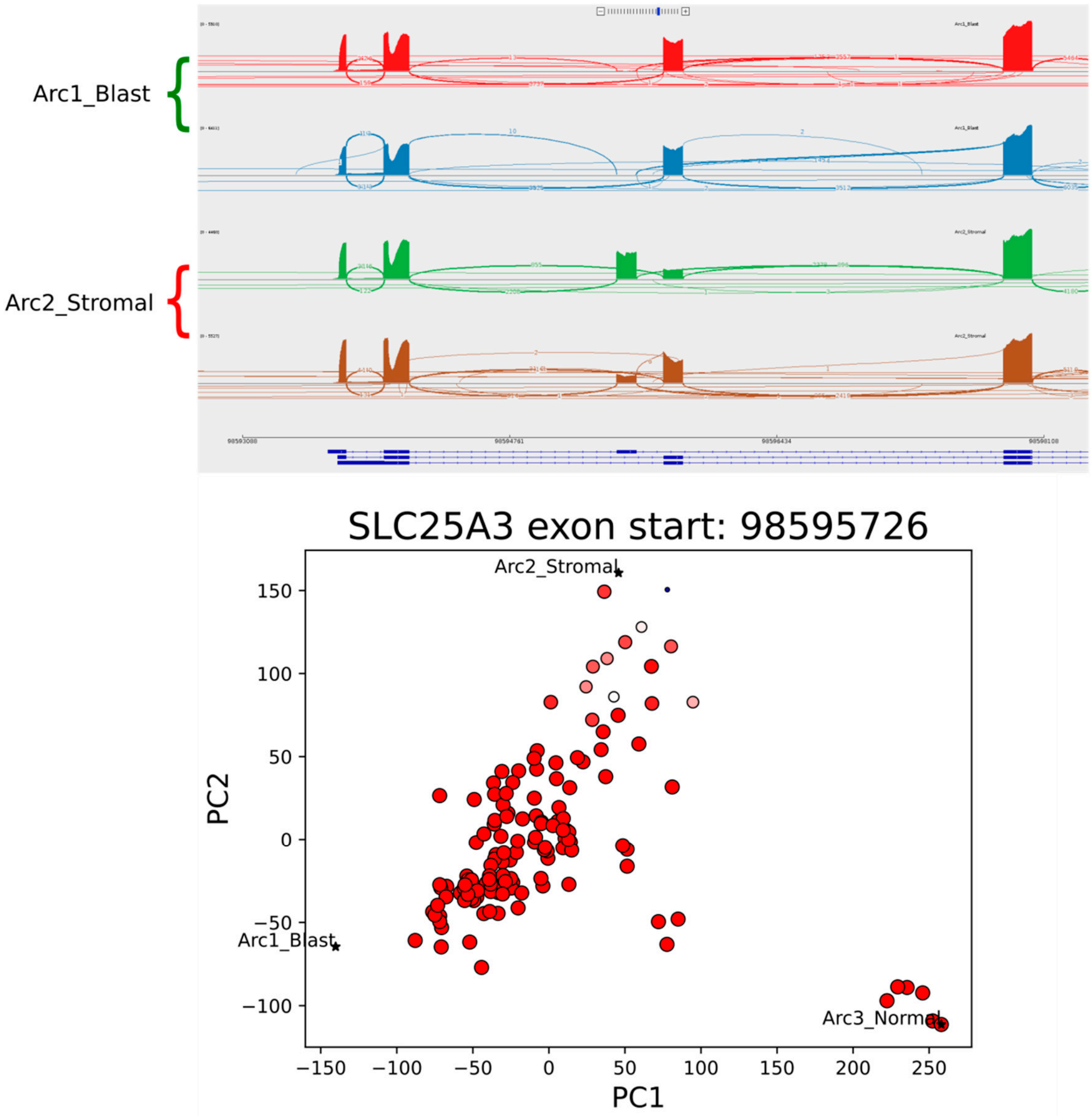

Figure S23: The gene SLC25A3 is alternatively spliced between samples located in different regions of latent space.

Top: A Shashimi plot of representative samples located near different archetypes. Bottom: a PCA plot of tumors and normal kidney samples, where each sample is marked according to the inclusion level of a selected mRNA isoform (large red – high, small blue – low).

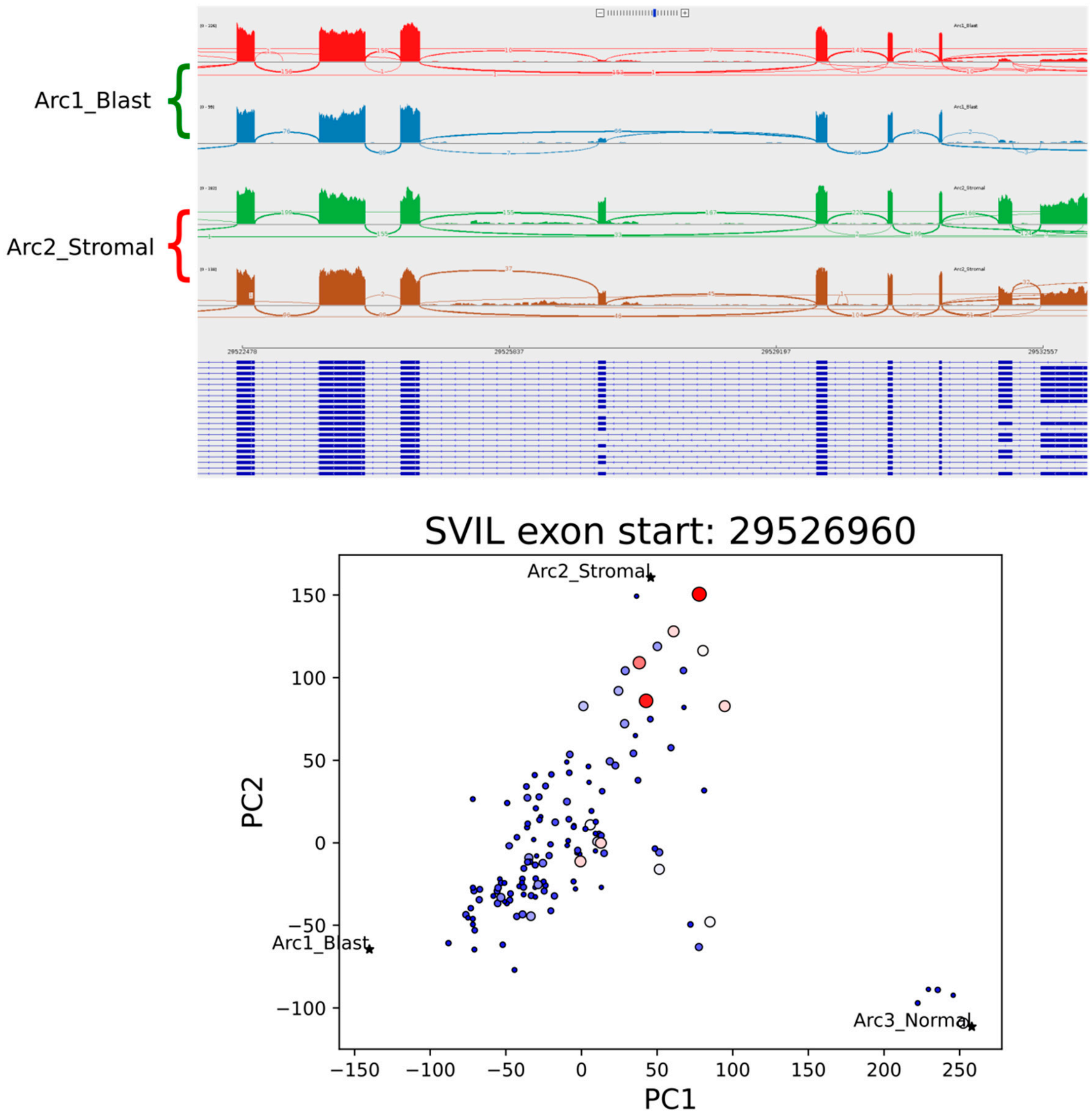

Figure S24: The gene SVIL is alternatively spliced between samples located in different regions of latent space.

Top: A Shashimi plot of representative samples located near different archetypes. Bottom: a PCA plot of tumors and normal kidney samples, where each sample is marked according to the inclusion level of a selected mRNA isoform (large red – high, small blue – low).

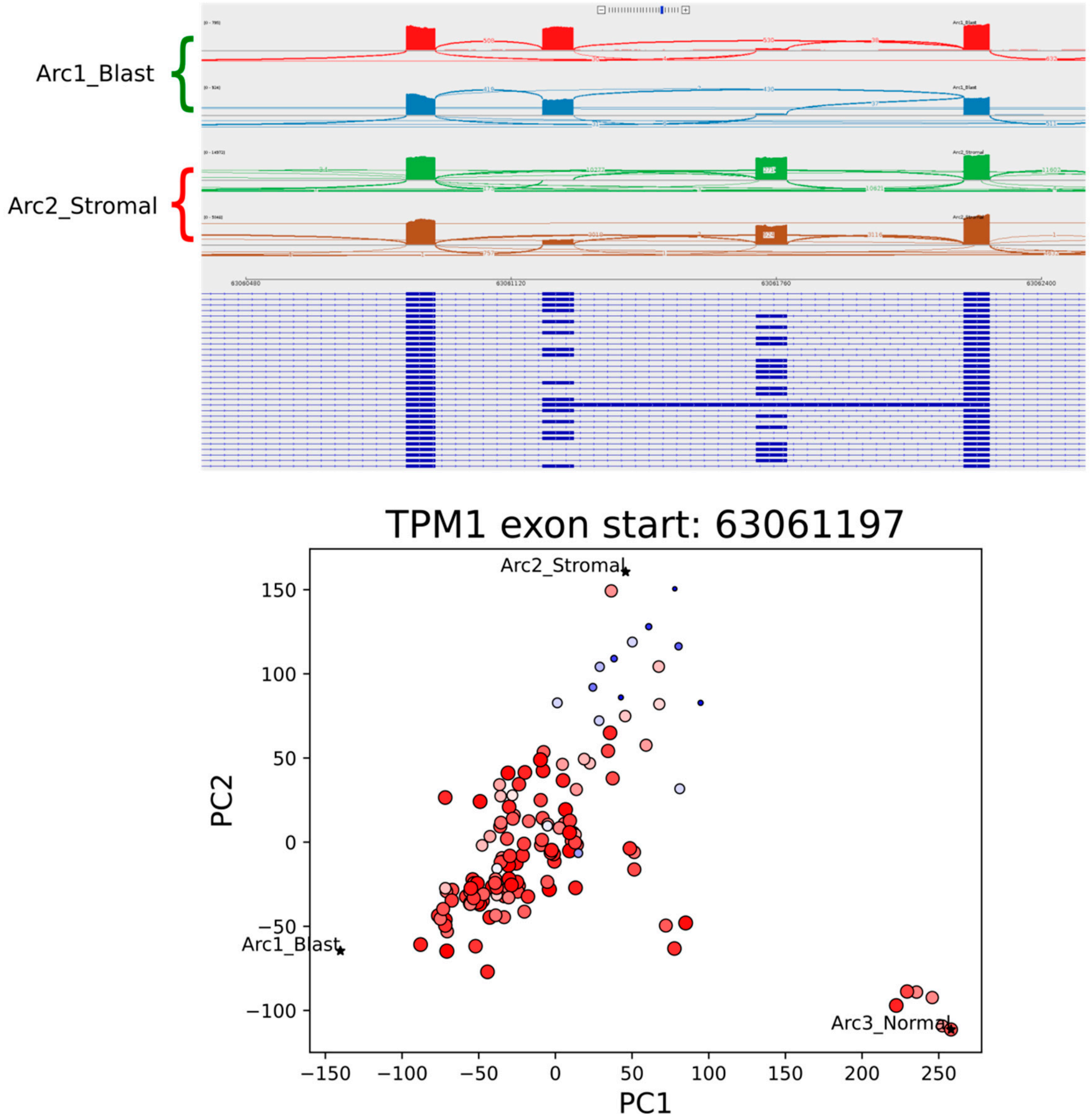

Figure S25: The gene TPM1 is alternatively spliced between samples located in different regions of latent space.

Top: A Shashimi plot of representative samples located near different archetypes. Bottom: a PCA plot of tumors and normal kidney samples, where each sample is marked according to the inclusion level of a selected mRNA isoform (large red – high, small blue – low).

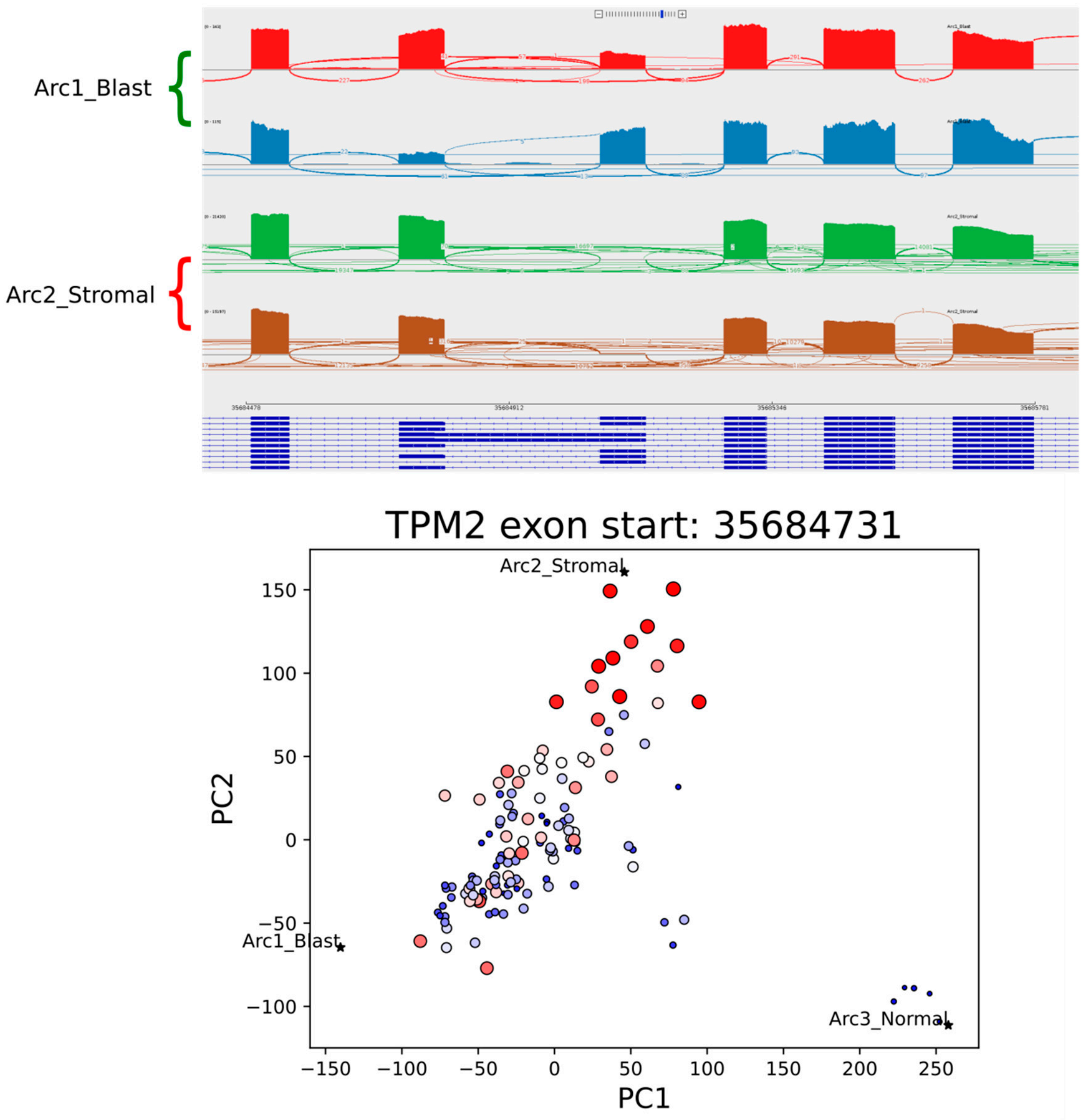

Figure S26: The gene TPM2 is alternatively spliced between samples located in different regions of latent space.

Top: A Shashimi plot of representative samples located near different archetypes. Bottom: a PCA plot of tumors and normal kidney samples, where each sample is marked according to the inclusion level of a selected mRNA isoform (large red – high, small blue – low).

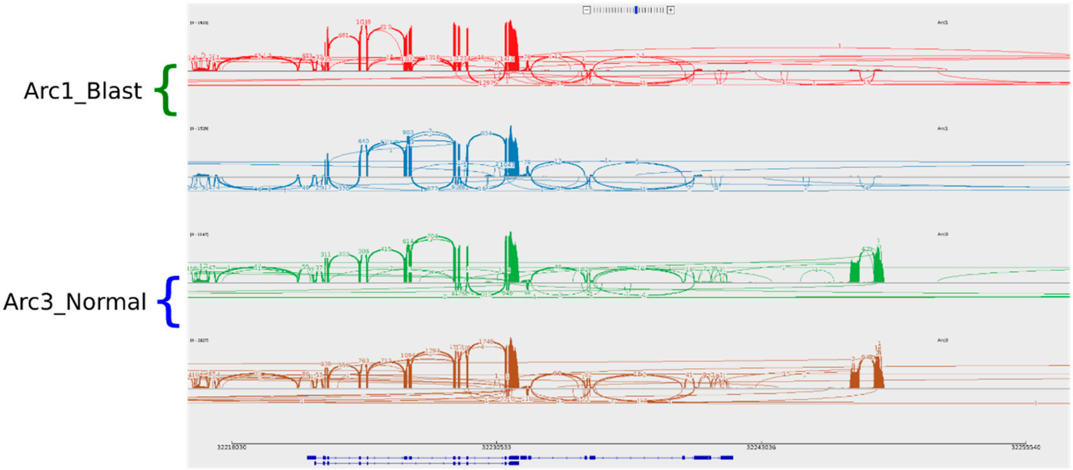

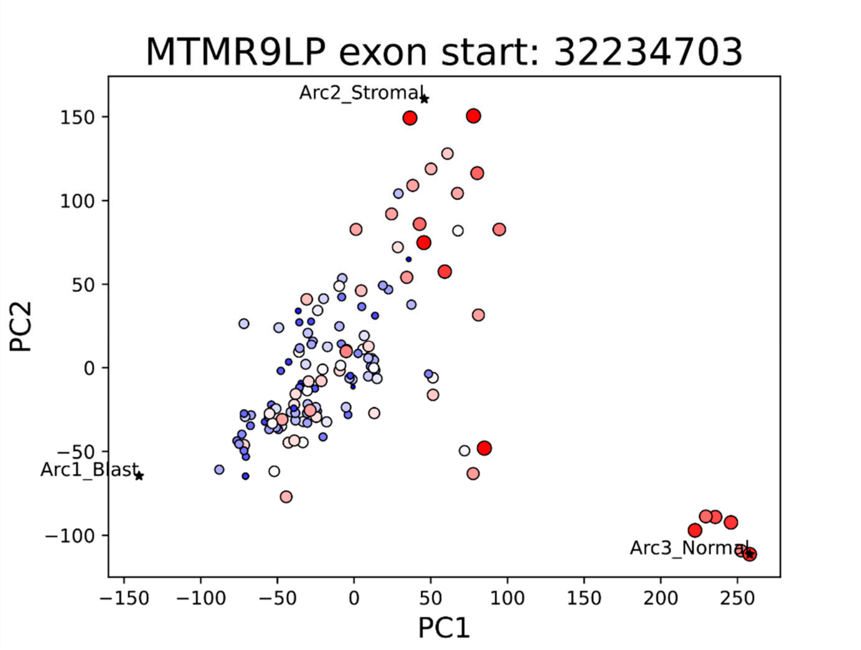

Figure S27: The gene MTMR9LP is alternatively spliced between samples located in different regions of latent space.

Top: A Shashimi plot of representative samples located near different archetypes. Bottom: a PCA plot of tumors and normal kidney samples, where each sample is marked according to the inclusion level of a selected mRNA isoform (large red – high, small blue – low).

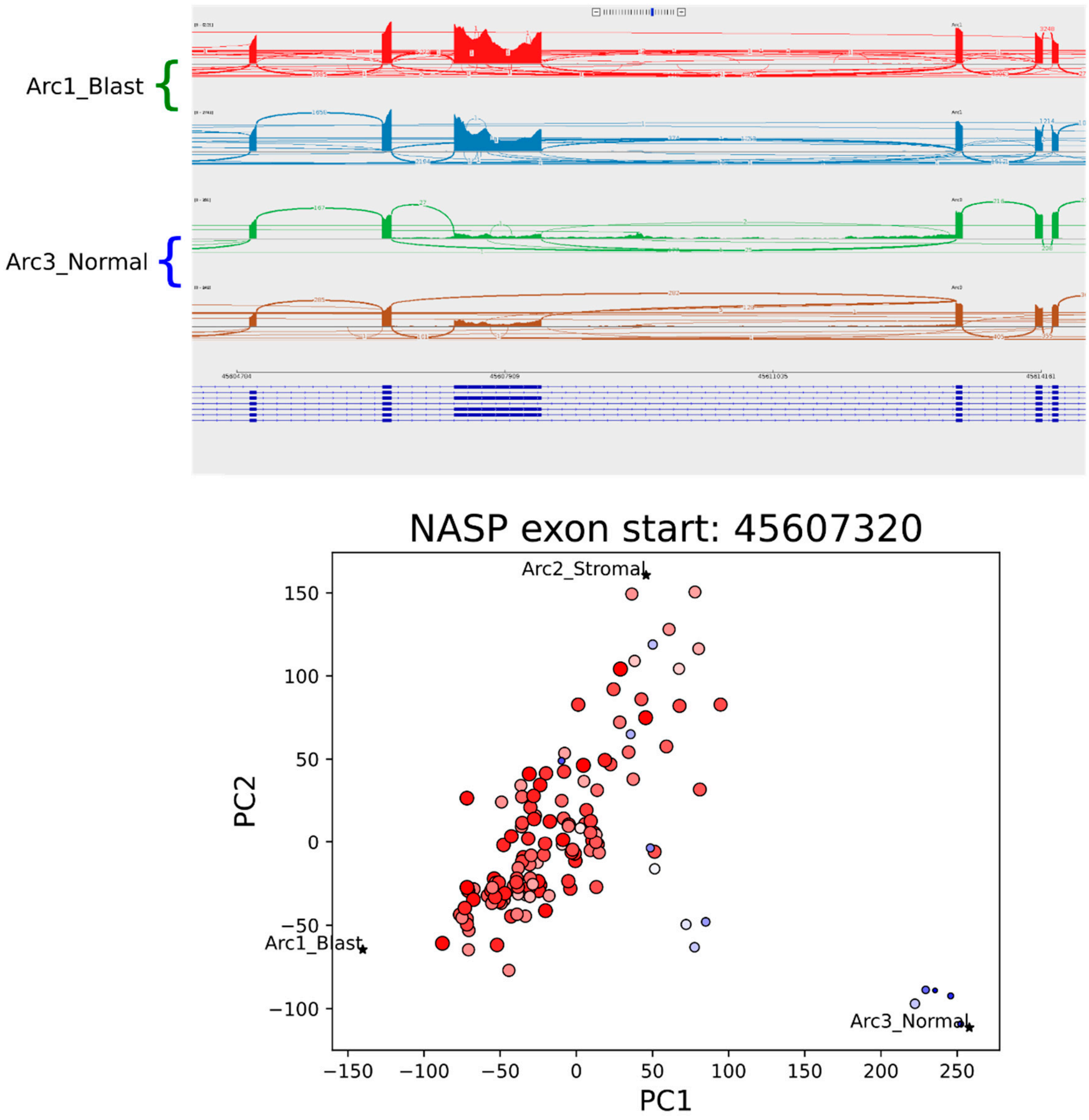

Figure S28: The gene NASP is alternatively spliced between samples located in different regions of latent space.

Top: A Shashimi plot of representative samples located near different archetypes. Bottom: a PCA plot of tumors and normal kidney samples, where each sample is marked according to the inclusion level of a selected mRNA isoform (large red – high, small blue – low).

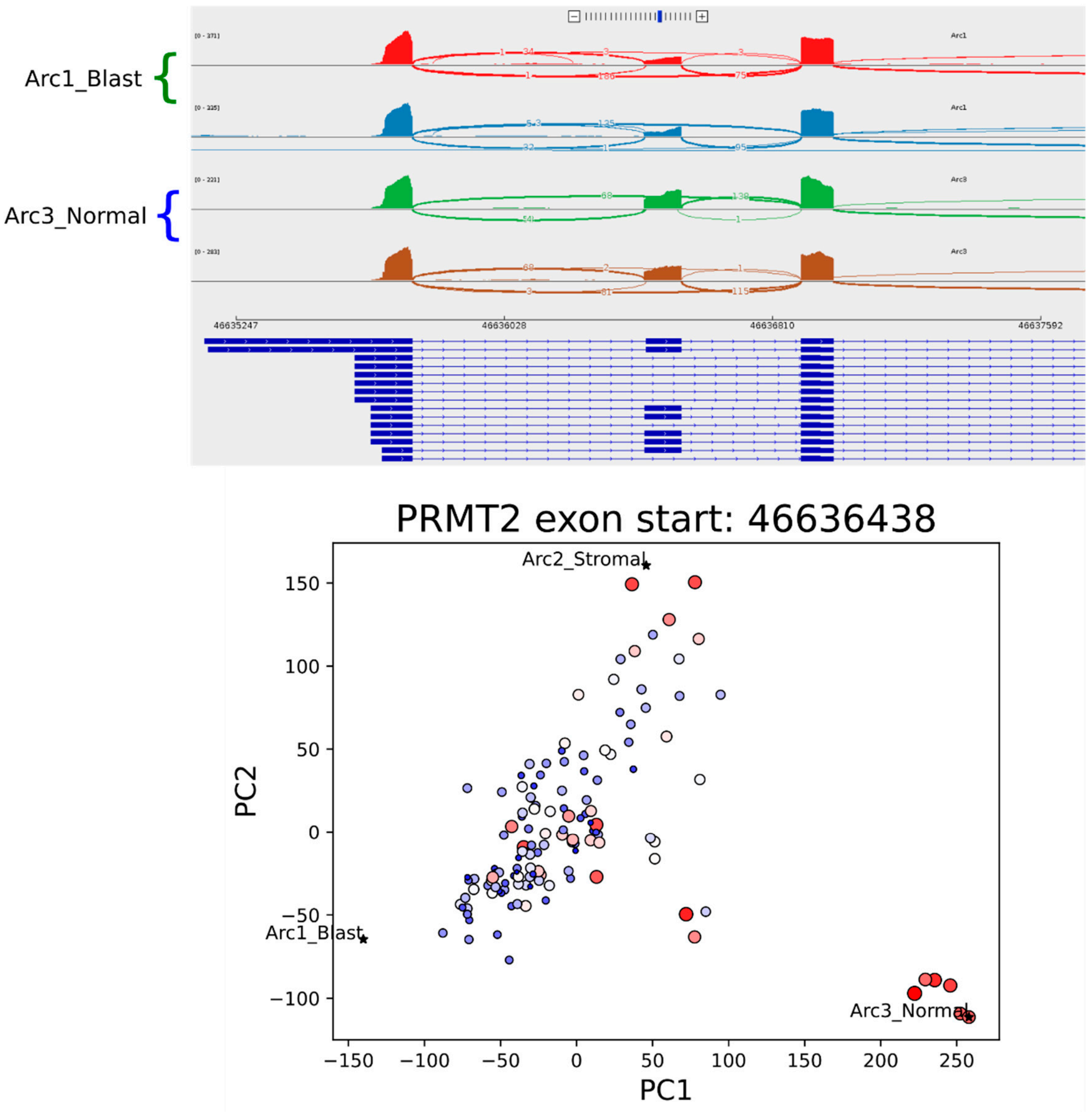

Figure S29: The gene PRMT2 is alternatively spliced between samples located in different regions of latent space.

Top: A Shashimi plot of representative samples located near different archetypes. Bottom: a PCA plot of tumors and normal kidney samples, where each sample is marked according to the inclusion level of a selected mRNA isoform (large red – high, small blue – low). This gene is known to be involved in cancer invasion, growth, and presumably EMT, and its splice variants have been linked to cancer [5], [6].

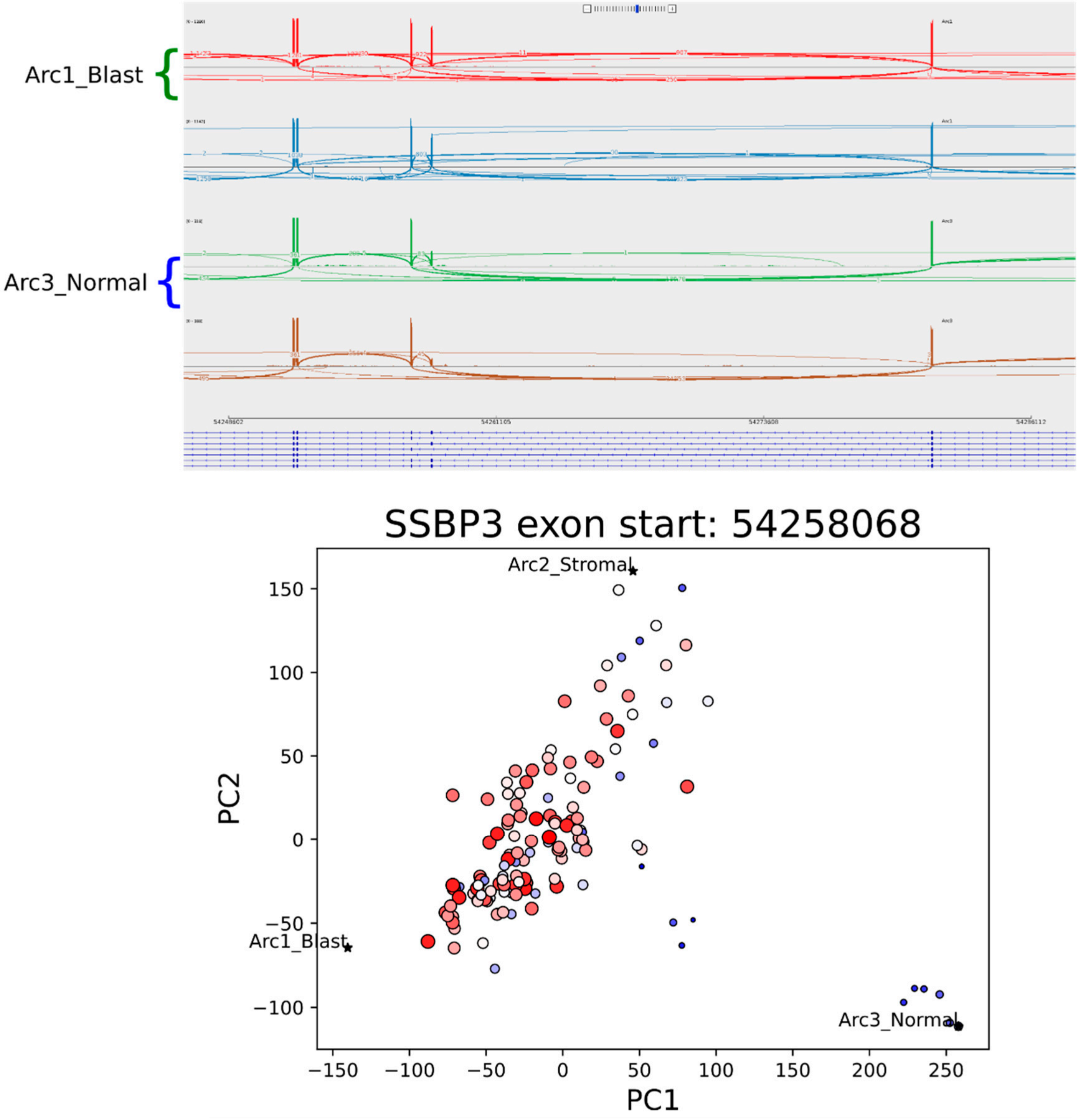

Figure S30: The gene SSBP3 is alternatively spliced between samples located in different regions of latent space.

Top: A Shashimi plot of representative samples located near different archetypes. Bottom: a PCA plot of tumors and normal kidney samples, where each sample is marked according to the inclusion level of a selected mRNA isoform (large red – high, small blue – low).

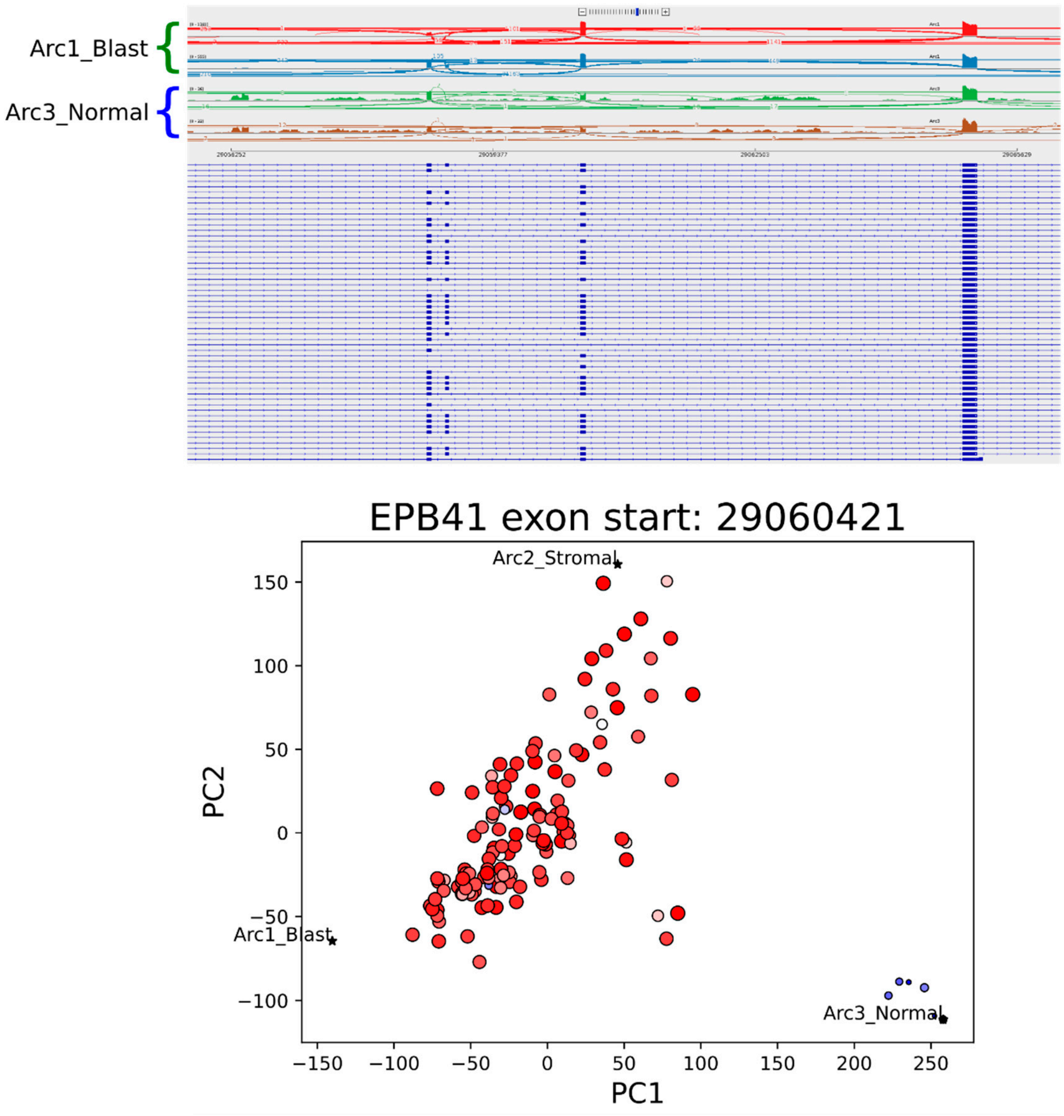

Figure S31: The gene EPB41 is alternatively spliced between samples located in different regions of latent space.

Top: A Shashimi plot of representative samples located near different archetypes. Bottom: a PCA plot of tumors and normal kidney samples, where each sample is marked according to the inclusion level of a selected mRNA isoform (large red – high, small blue – low).

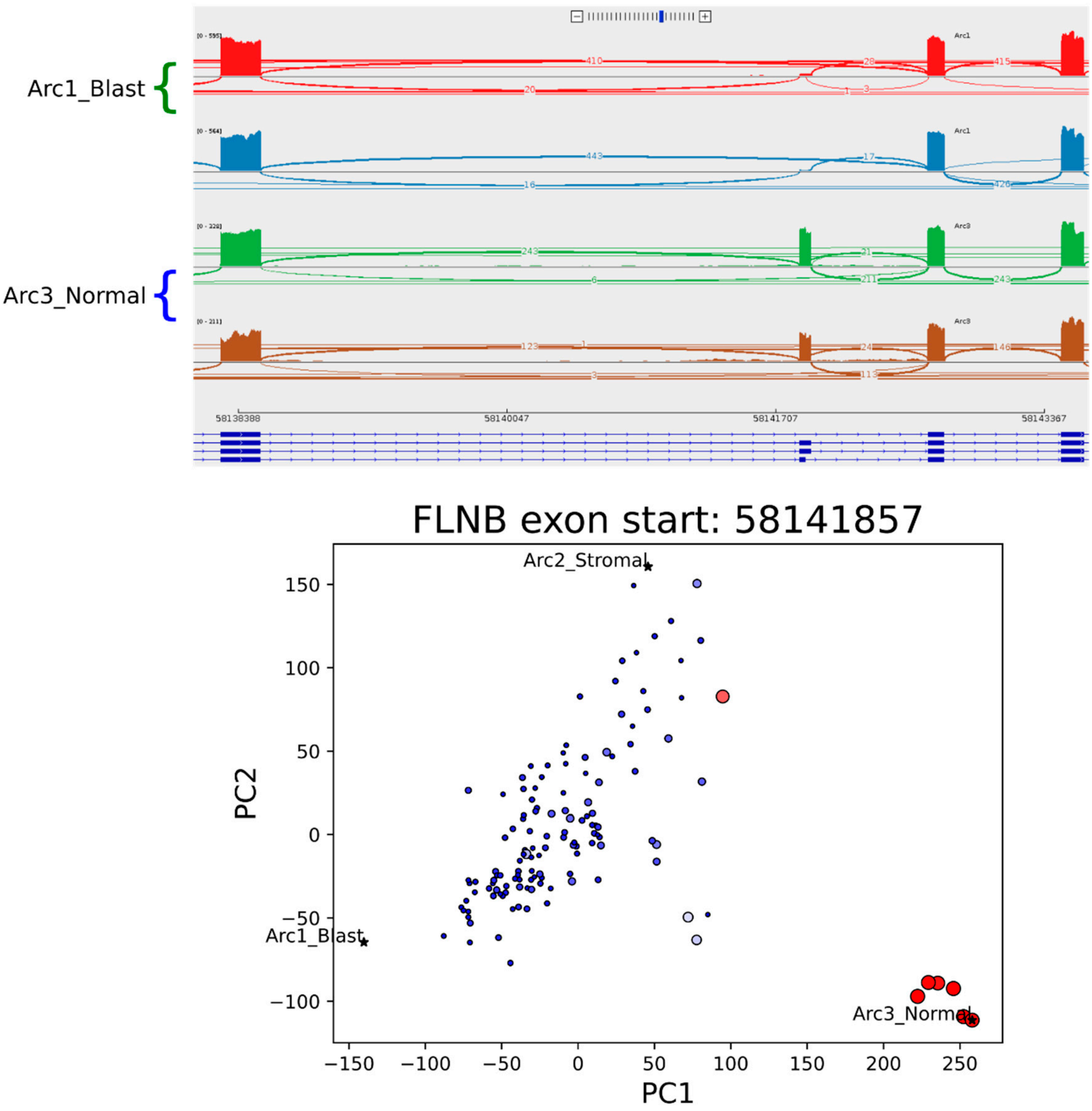

Figure S32: The gene FLNB is alternatively spliced between samples located in different regions of latent space.

Top: A Shashimi plot of representative samples located near different archetypes. Bottom: a PCA plot of tumors and normal kidney samples, where each sample is marked according to the inclusion level of a selected mRNA isoform (large red – high, small blue – low). These results are consistent with previous observations that skipping of exon 30 of FLNB induces EMT in human mammary epithelial cells, is associated with basal-like breast cancer, and is regulated by the RNA binding proteins QKI and RBFOX1 [7].

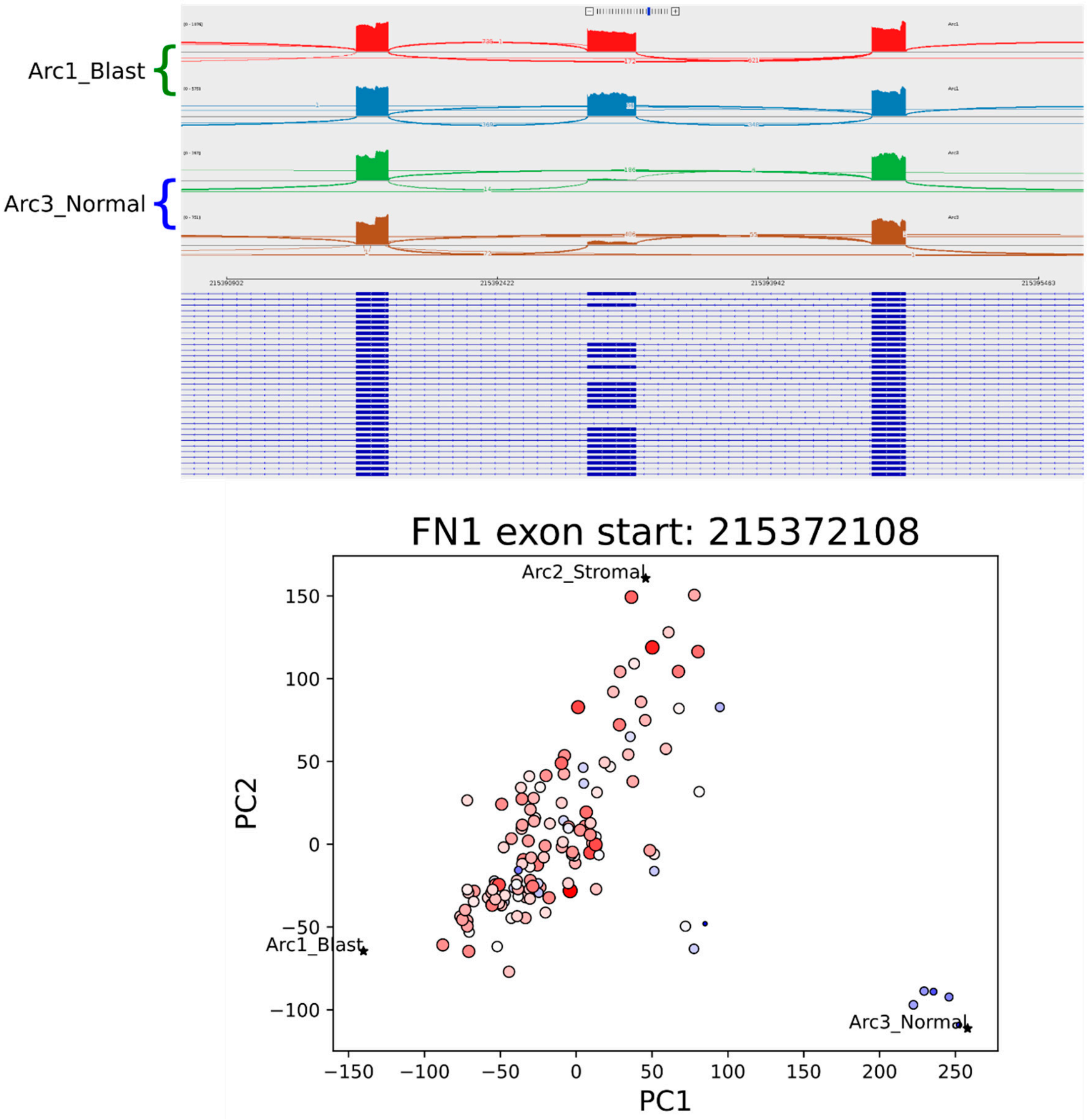

Figure S33: The gene FN1 is alternatively spliced between samples located in different regions of latent space.

Top: A Shashimi plot of representative samples located near different archetypes. Bottom: a PCA plot of tumors and normal kidney samples, where each sample is marked according to the inclusion level of a selected mRNA isoform (large red – high, small blue – low).

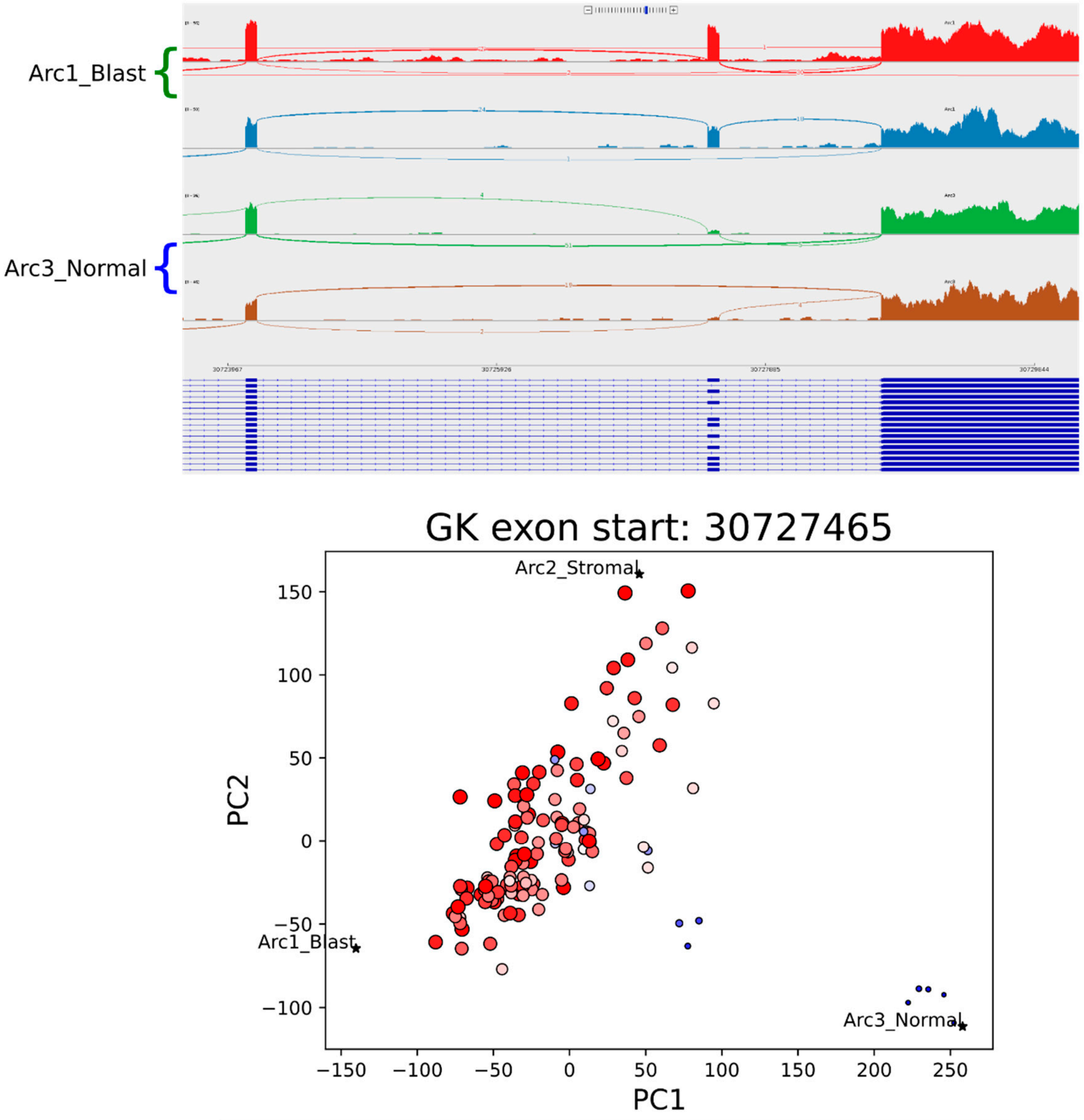

Figure S34: The gene GK is alternatively spliced between samples located in different regions of latent space.

Top: A Shashimi plot of representative samples located near different archetypes. Bottom: a PCA plot of tumors and normal kidney samples, where each sample is marked according to the inclusion level of a selected mRNA isoform (large red – high, small blue – low).

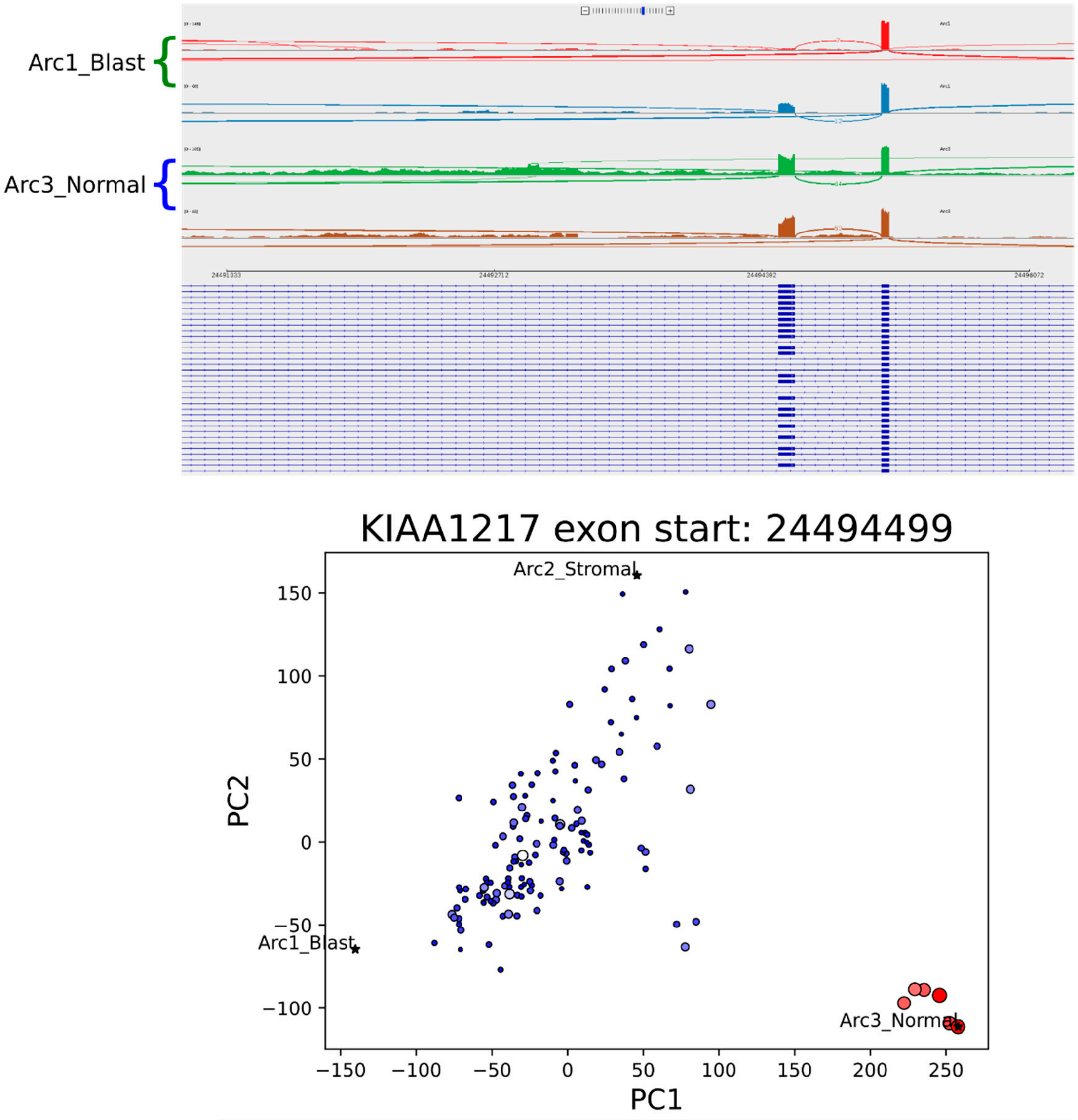

Figure S35: The gene KIAA1217 is alternatively spliced between samples located in different regions of latent space.

Top: A Shashimi plot of representative samples located near different archetypes. Bottom: a PCA plot of tumors and normal kidney samples, where each sample is marked according to the inclusion level of a selected mRNA isoform (large red – high, small blue – low).

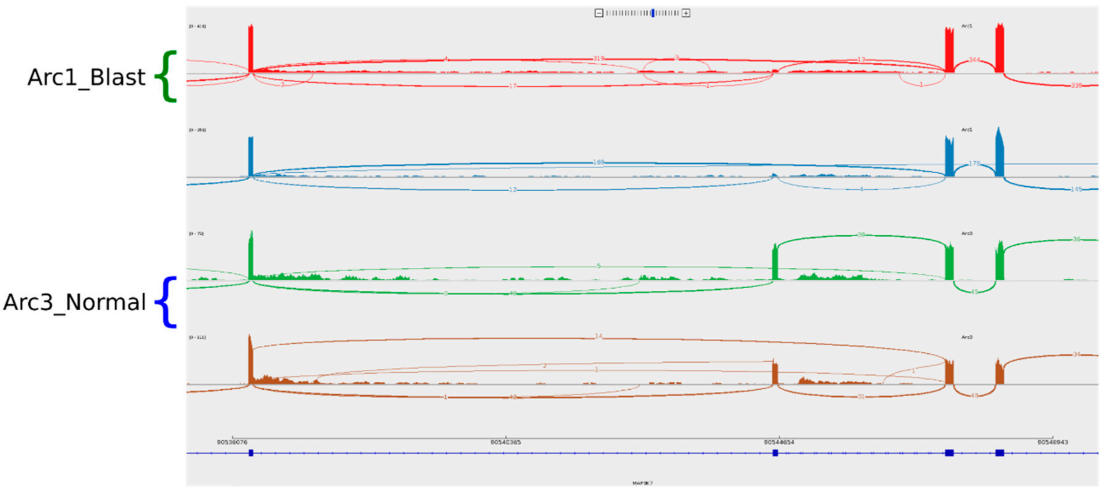

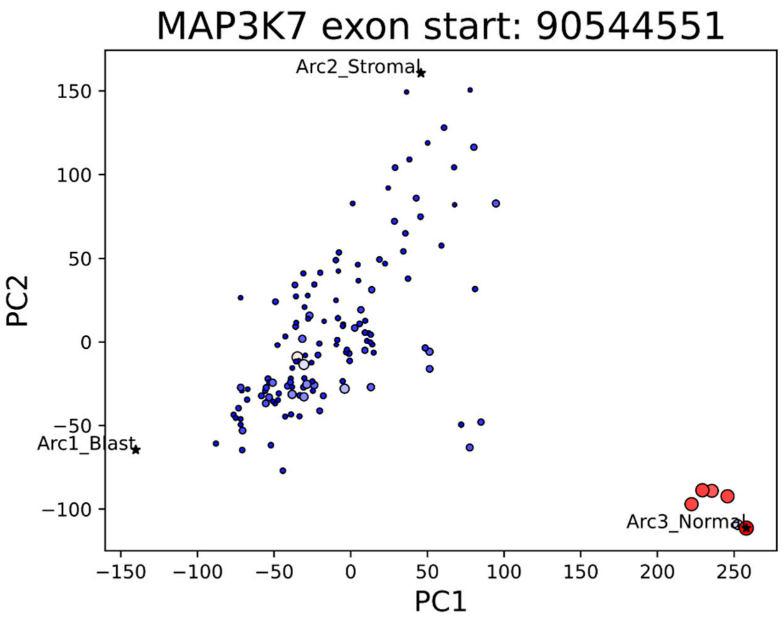

Figure S36: The gene MAP3K7 is alternatively spliced between samples located in different regions of latent space.

Top: A Shashimi plot of representative samples located near different archetypes. Bottom: a PCA plot of tumors and normal kidney samples, where each sample is marked according to the inclusion level of a selected mRNA isoform (large red – high, small blue – low). This gene is known to have muscle-specific transcripts that are dependent on the splicing regulator RBFOX2 [3].

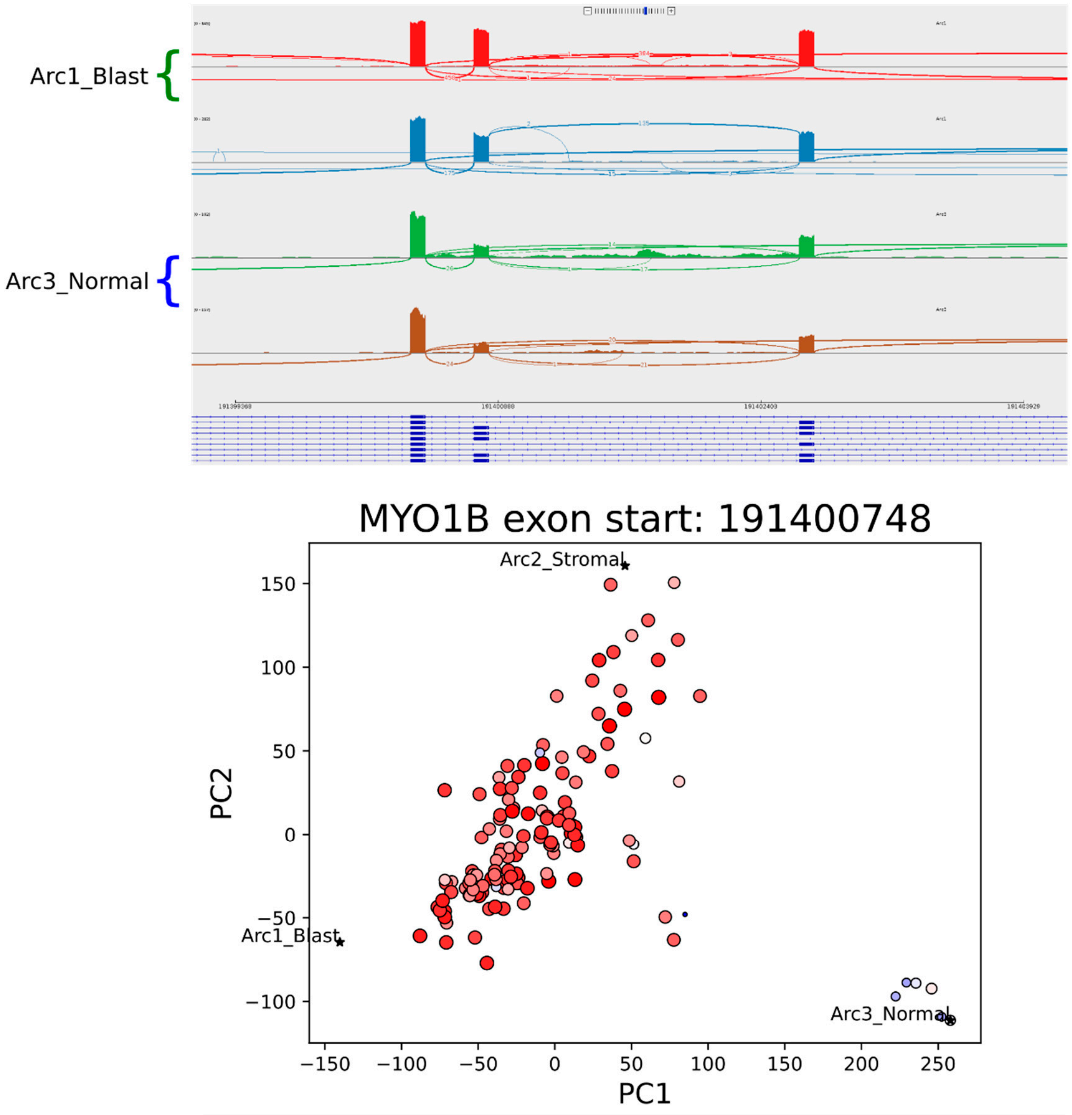

Figure S37: The gene MYO1B is alternatively spliced between samples located in different regions of latent space.

Top: A Shashimi plot of representative samples located near different archetypes. Bottom: a PCA plot of tumors and normal kidney samples, where each sample is marked according to the inclusion level of a selected mRNA isoform (large red – high, small blue – low).

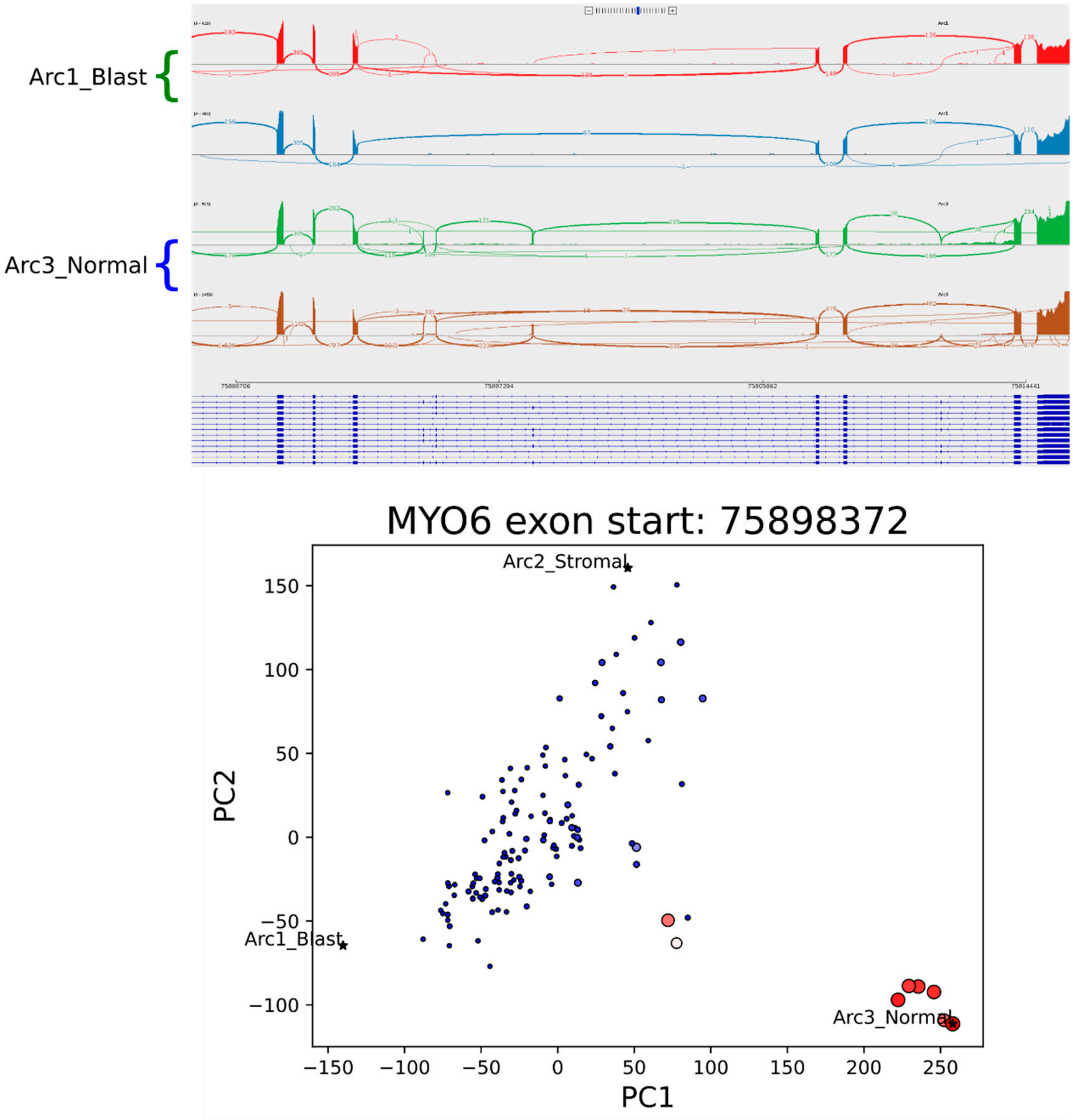

Figure S38: The gene MYO6 is alternatively spliced between samples located in different regions of latent space.

Top: A Shashimi plot of representative samples located near different archetypes. Bottom: a PCA plot of tumors and normal kidney samples, where each sample is marked according to the inclusion level of a selected mRNA isoform (large red – high, small blue – low).

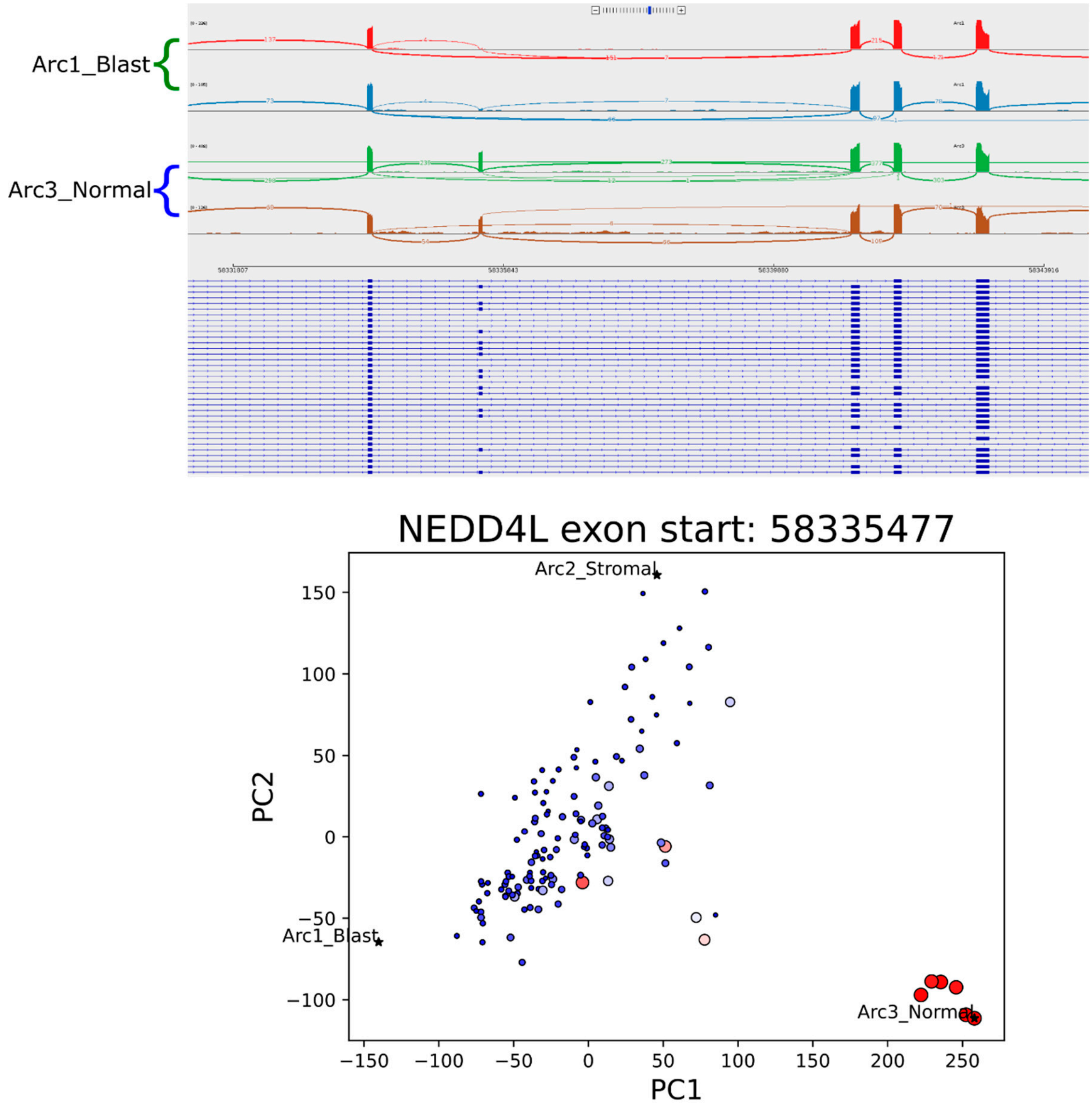

Figure S39: The gene NEDD4L is alternatively spliced between samples located in different regions of latent space.

Top: A Shashimi plot of representative samples located near different archetypes. Bottom: a PCA plot of tumors and normal kidney samples, where each sample is marked according to the inclusion level of a selected mRNA isoform (large red – high, small blue – low).

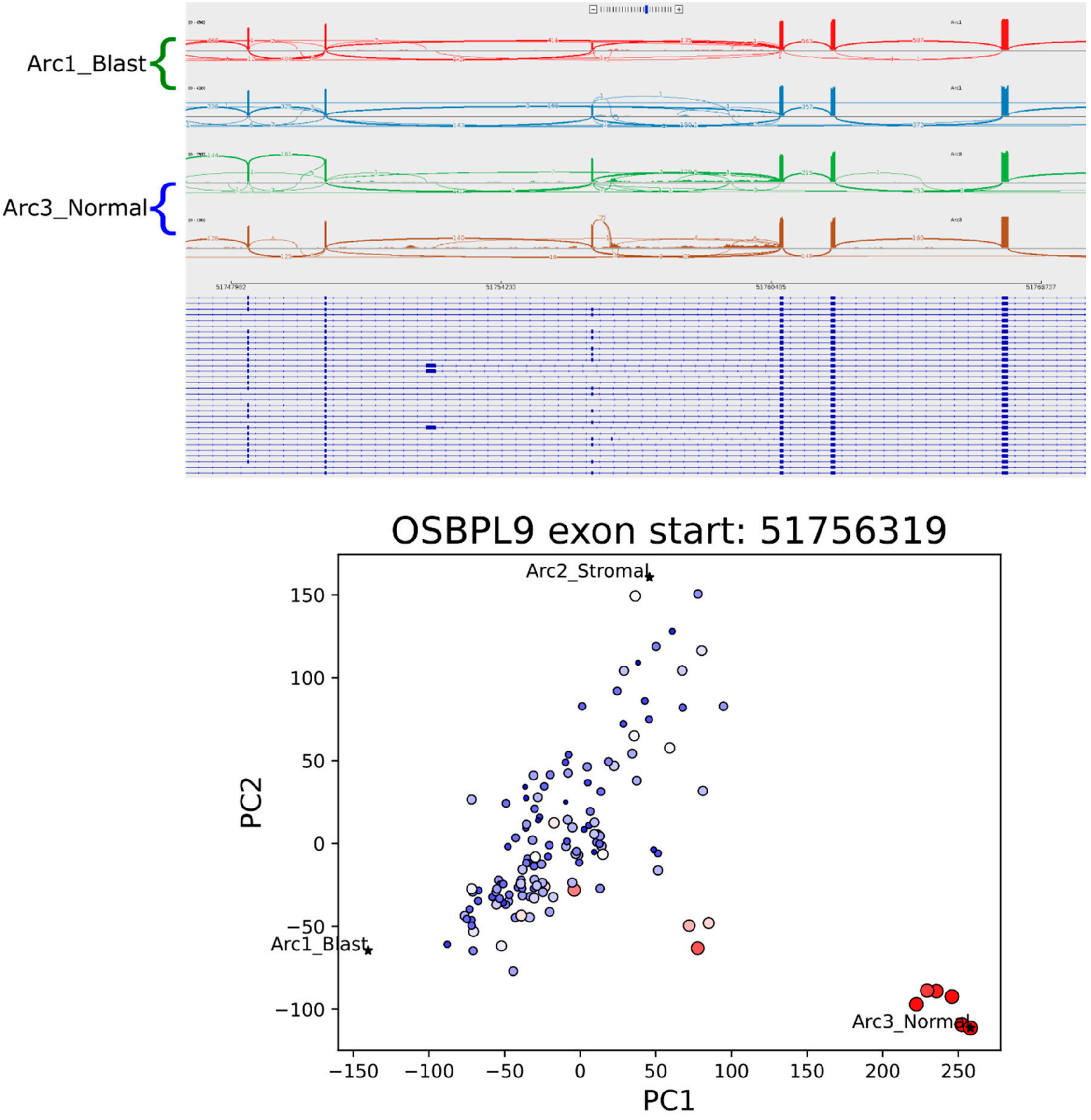

Figure S40: The gene OSBPL9 is alternatively spliced between samples located in different regions of latent space.

Top: A Shashimi plot of representative samples located near different archetypes. Bottom: a PCA plot of tumors and normal kidney samples, where each sample is marked according to the inclusion level of a selected mRNA isoform (large red – high, small blue – low).

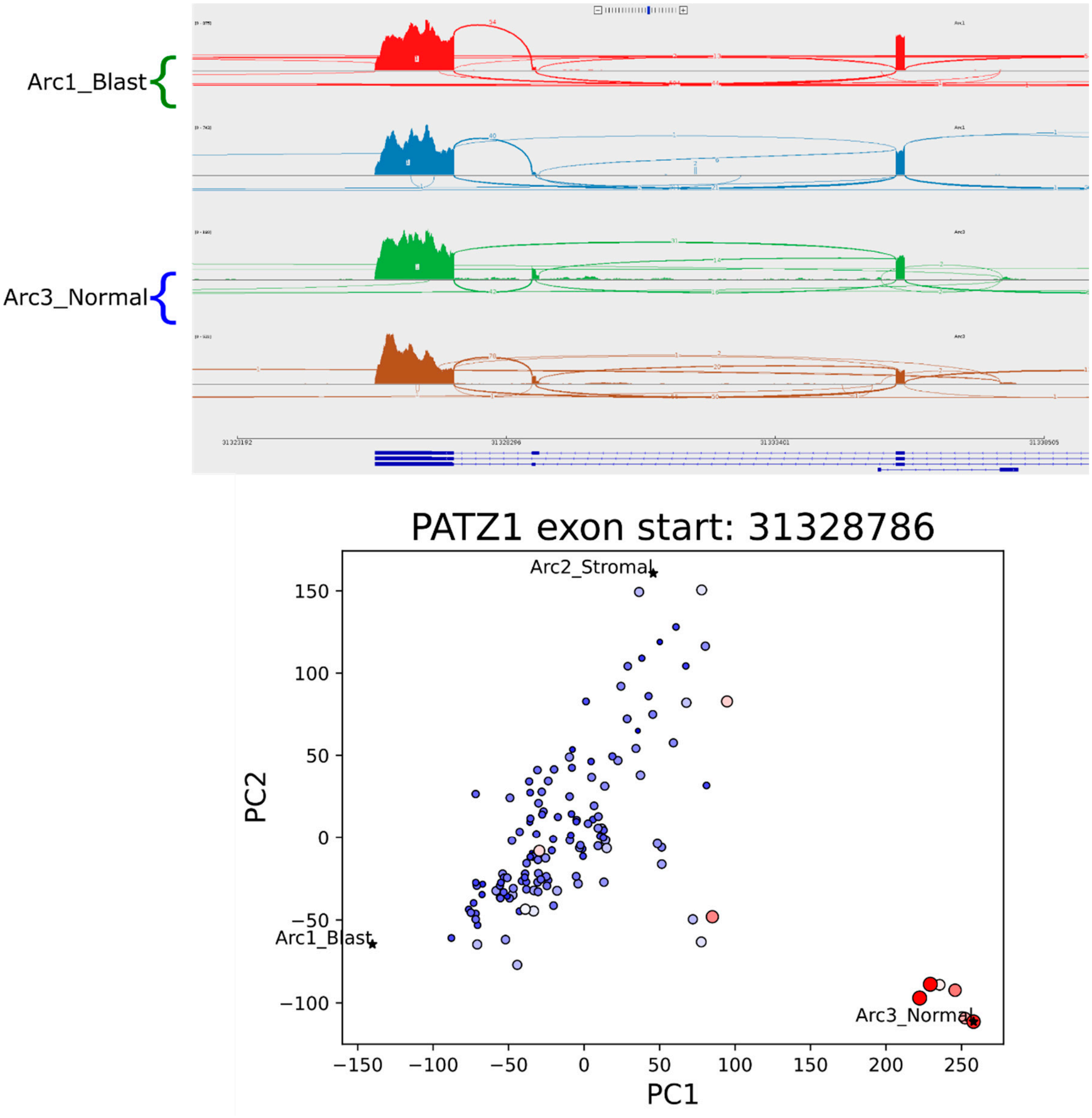

Figure S41: The gene PATZ1 is alternatively spliced between samples located in different regions of latent space.

Top: A Shashimi plot of representative samples located near different archetypes. Bottom: a PCA plot of tumors and normal kidney samples, where each sample is marked according to the inclusion level of a selected mRNA isoform (large red – high, small blue – low).

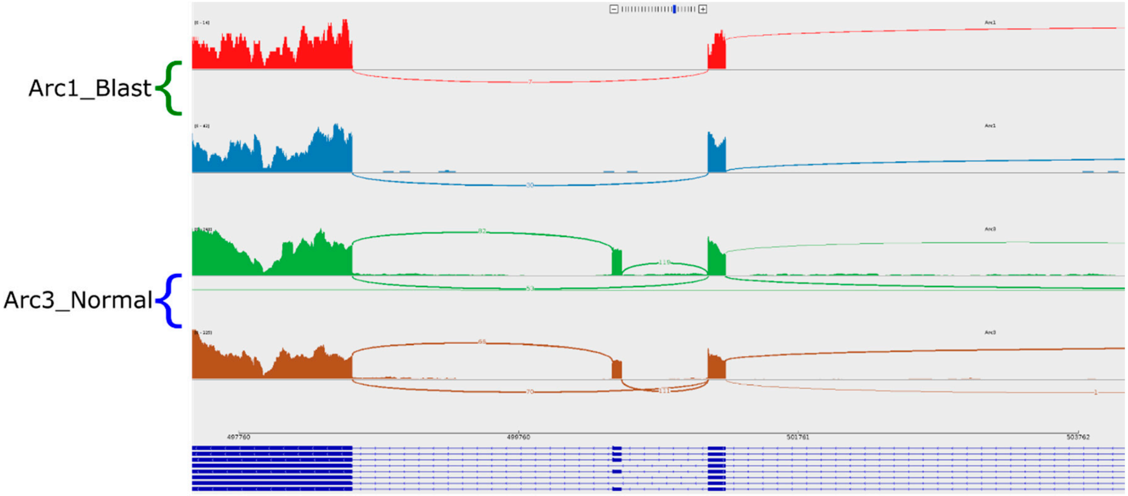

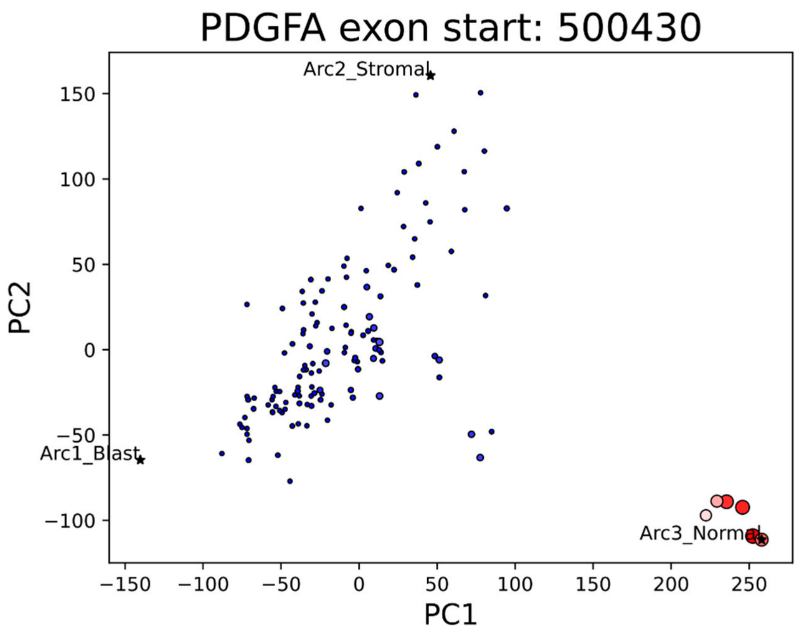

Figure S42: The gene PDGFA is alternatively spliced between samples located in different regions of latent space.

Top: A Shashimi plot of representative samples located near different archetypes. Bottom: a PCA plot of tumors and normal kidney samples, where each sample is marked according to the inclusion level of a selected mRNA isoform (large red – high, small blue – low).

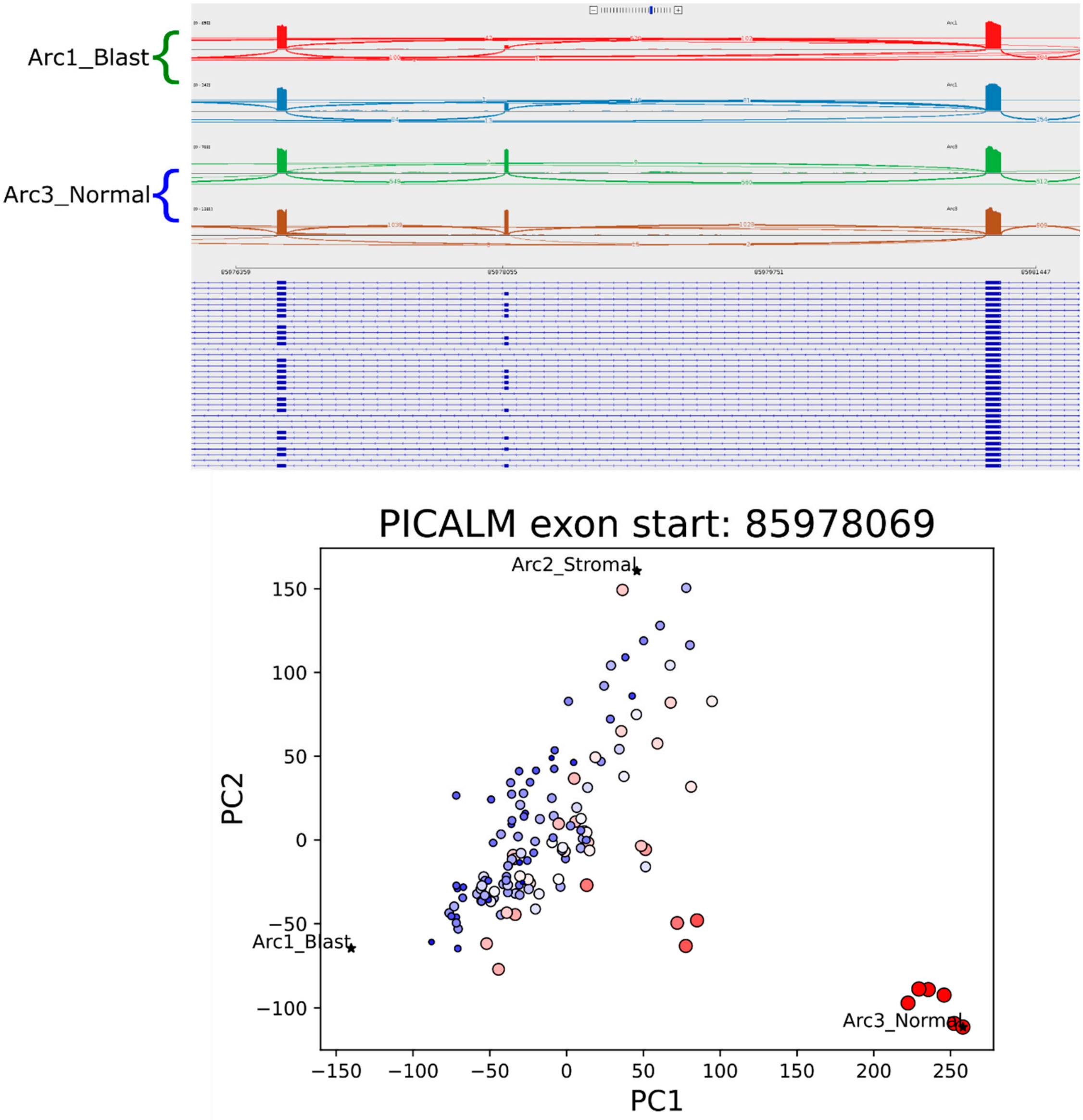

Figure S43: The gene PICALM is alternatively spliced between samples located in different regions of latent space.

Top: A Shashimi plot of representative samples located near different archetypes. Bottom: a PCA plot of tumors and normal kidney samples, where each sample is marked according to the inclusion level of a selected mRNA isoform (large red – high, small blue – low).

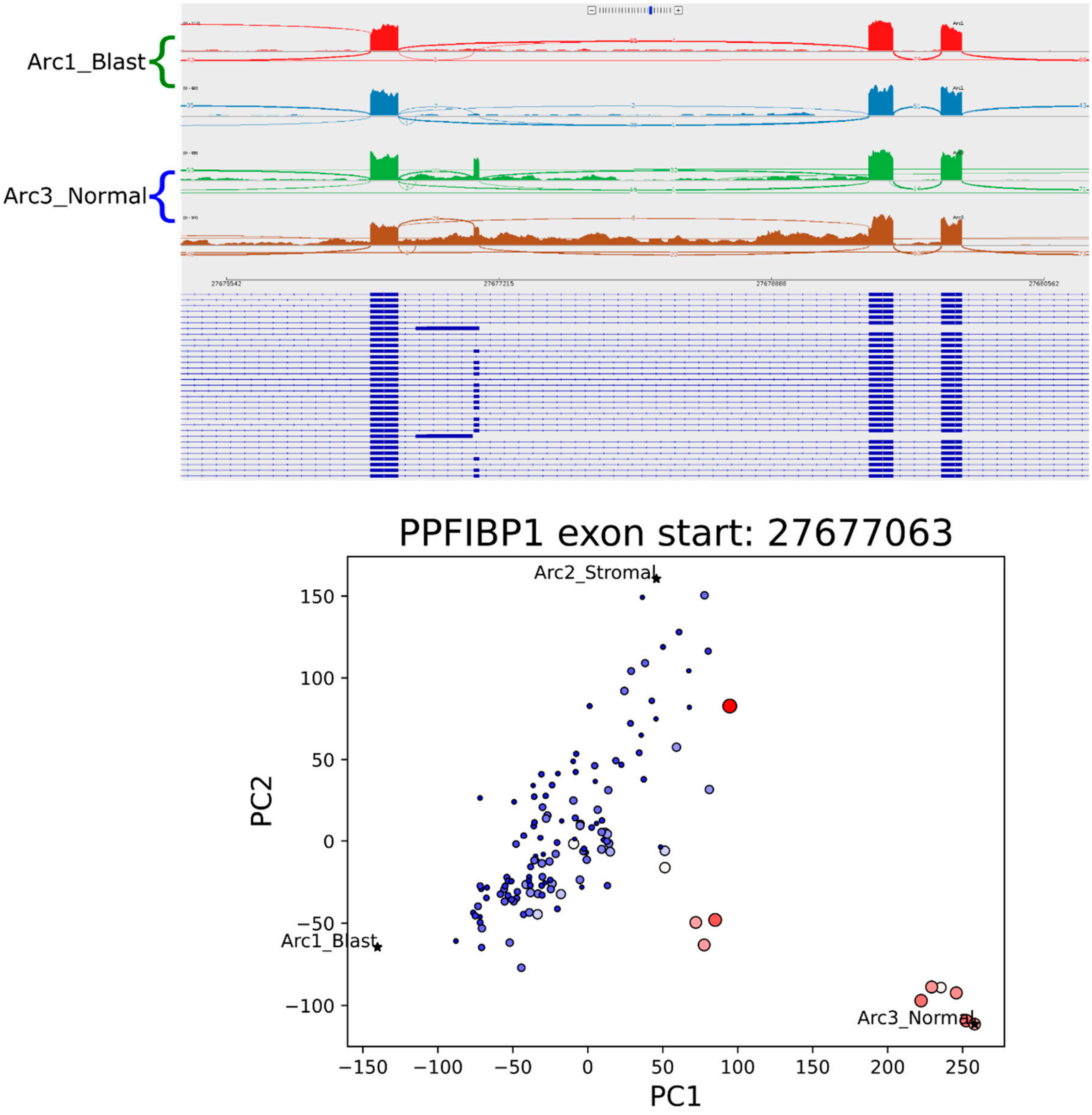

Figure S44: The gene PPFIBP1 is alternatively spliced between samples located in different regions of latent space.

Top: A Shashimi plot of representative samples located near different archetypes. Bottom: a PCA plot of tumors and normal kidney samples, where each sample is marked according to the inclusion level of a selected mRNA isoform (large red – high, small blue – low).

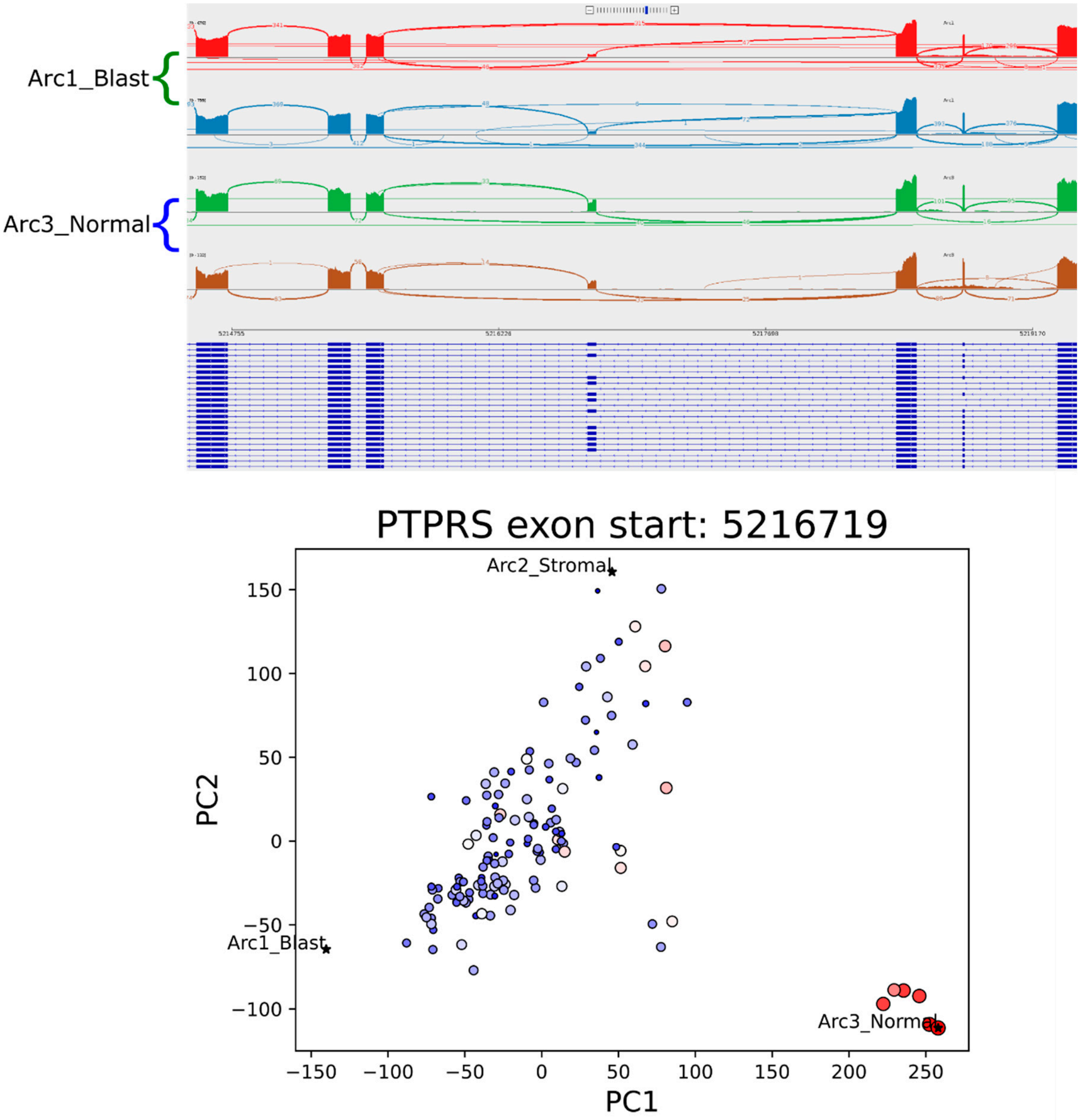

Figure S45: The gene PTPRS is alternatively spliced between samples located in different regions of latent space.

Top: A Shashimi plot of representative samples located near different archetypes. Bottom: a PCA plot of tumors and normal kidney samples, where each sample is marked according to the inclusion level of a selected mRNA isoform (large red – high, small blue – low).

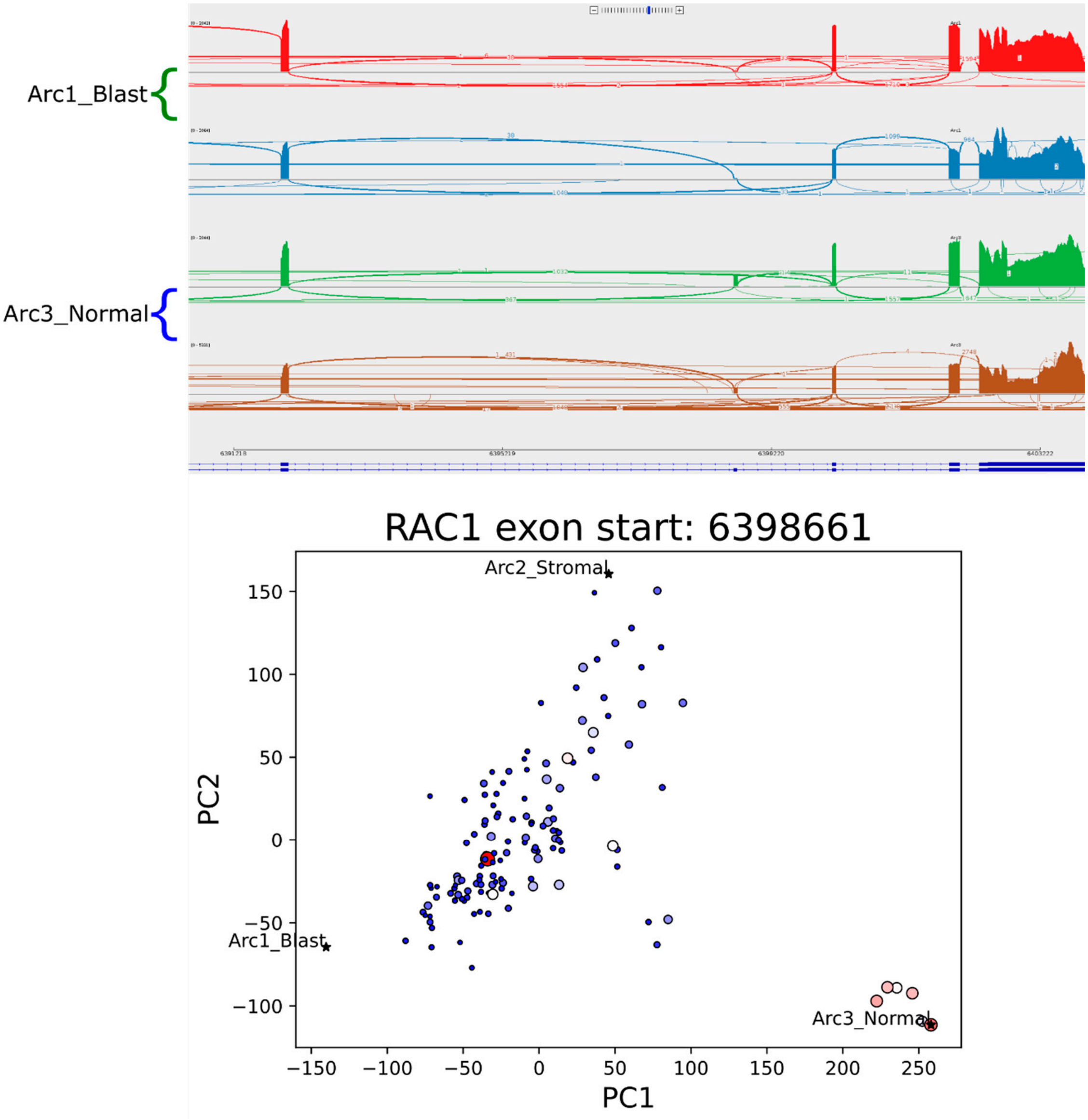

Figure S46: The gene RAC1 [8] is alternatively spliced between samples located in different regions of latent space.

Top: A Shashimi plot of representative samples located near different archetypes. Bottom: a PCA plot of tumors and normal kidney samples, where each sample is marked according to the inclusion level of a selected mRNA isoform (large red – high, small blue – low).

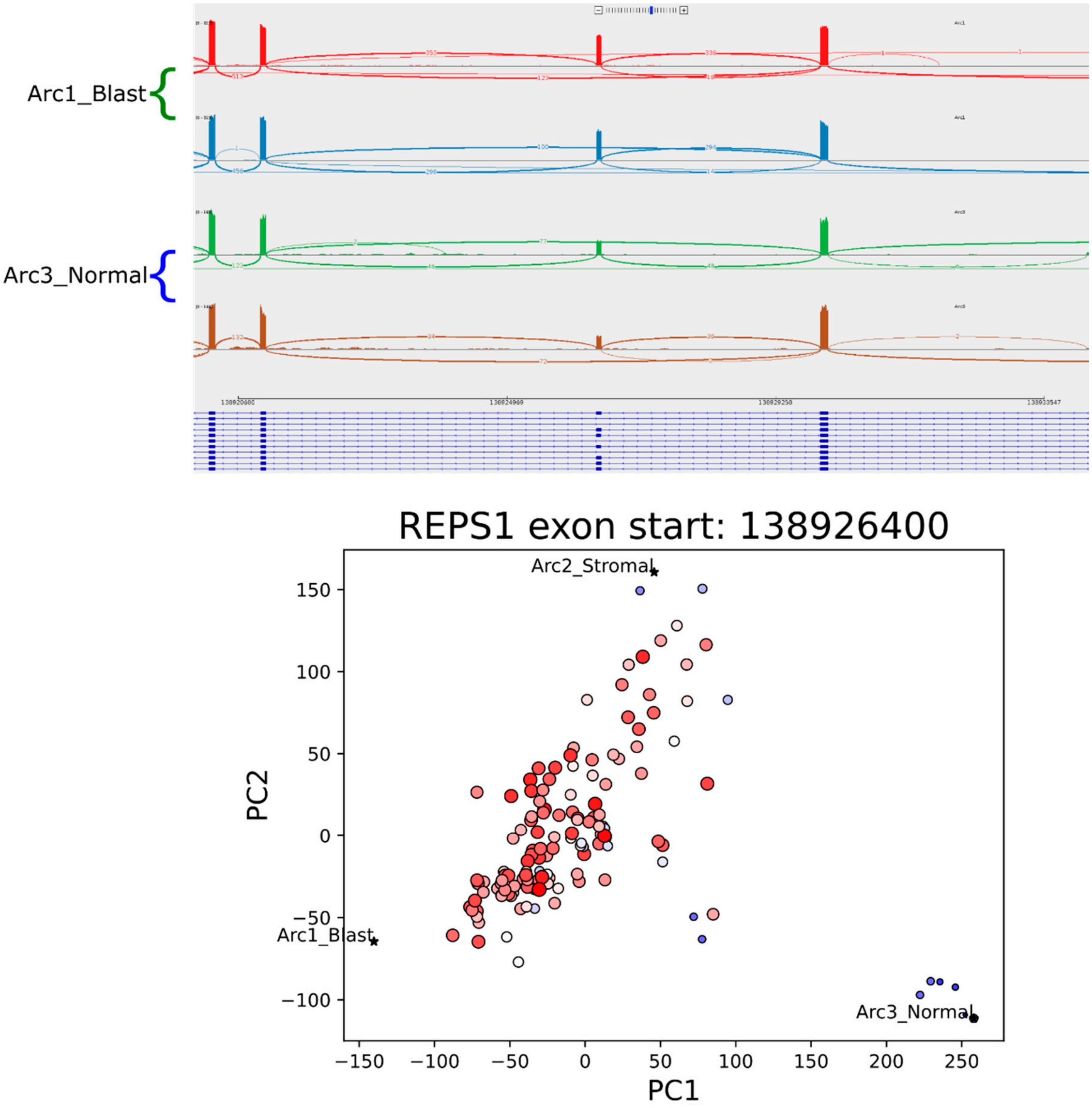

Figure S47: The gene REPS1 is alternatively spliced between samples located in different regions of latent space.

Top: A Shashimi plot of representative samples located near different archetypes. Bottom: a PCA plot of tumors and normal kidney samples, where each sample is marked according to the inclusion level of a selected mRNA isoform (large red – high, small blue – low).

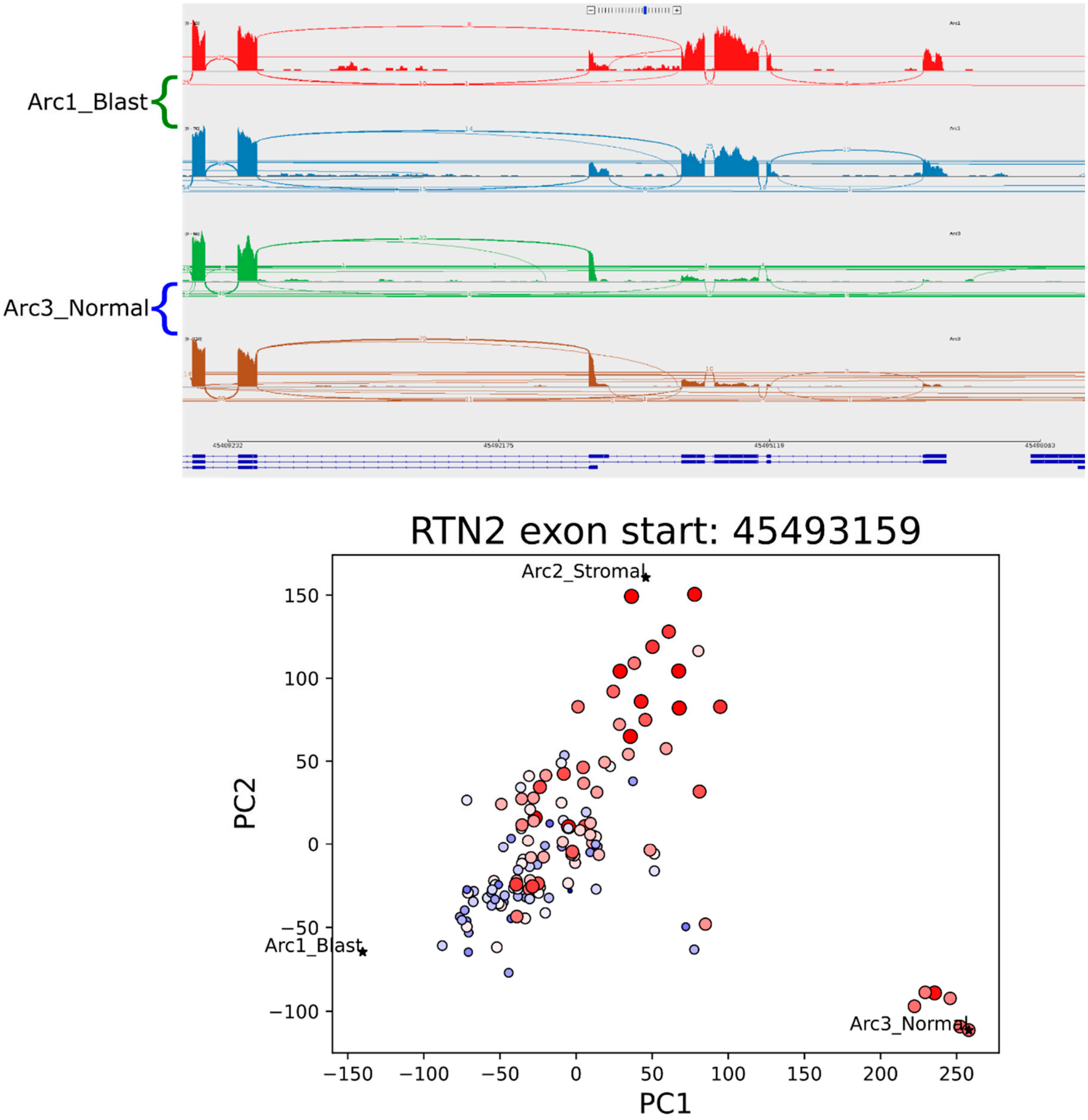

Figure S48: The gene RTN2 is alternatively spliced between samples located in different regions of latent space.

Top: A Shashimi plot of representative samples located near different archetypes. Bottom: a PCA plot of tumors and normal kidney samples, where each sample is marked according to the inclusion level of a selected mRNA isoform (large red – high, small blue – low).

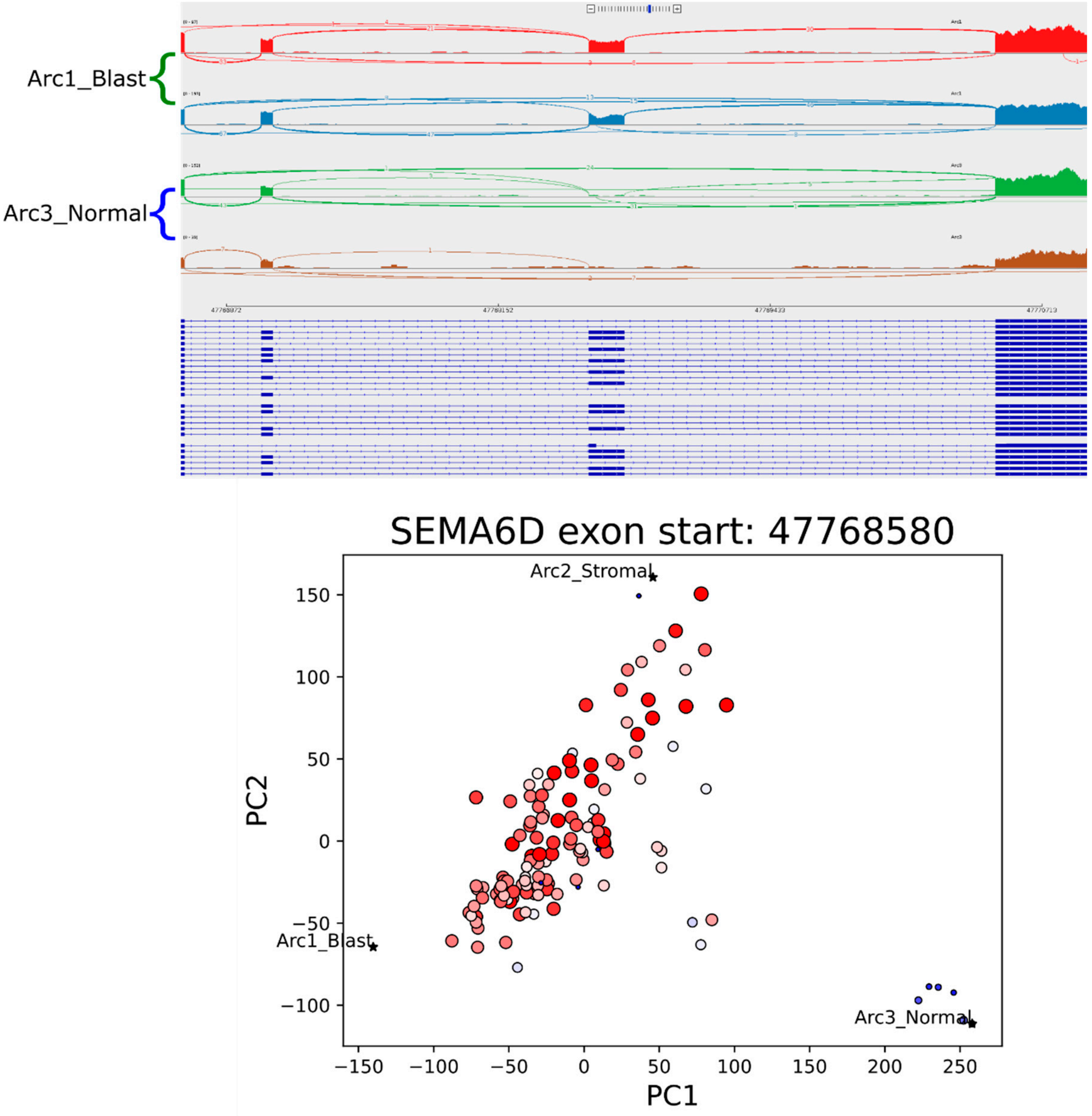

Figure S49: The gene SEMA6D is alternatively spliced between samples located in different regions of latent space.

Top: A Shashimi plot of representative samples located near different archetypes. Bottom: a PCA plot of tumors and normal kidney samples, where each sample is marked according to the inclusion level of a selected mRNA isoform (large red – high, small blue – low).

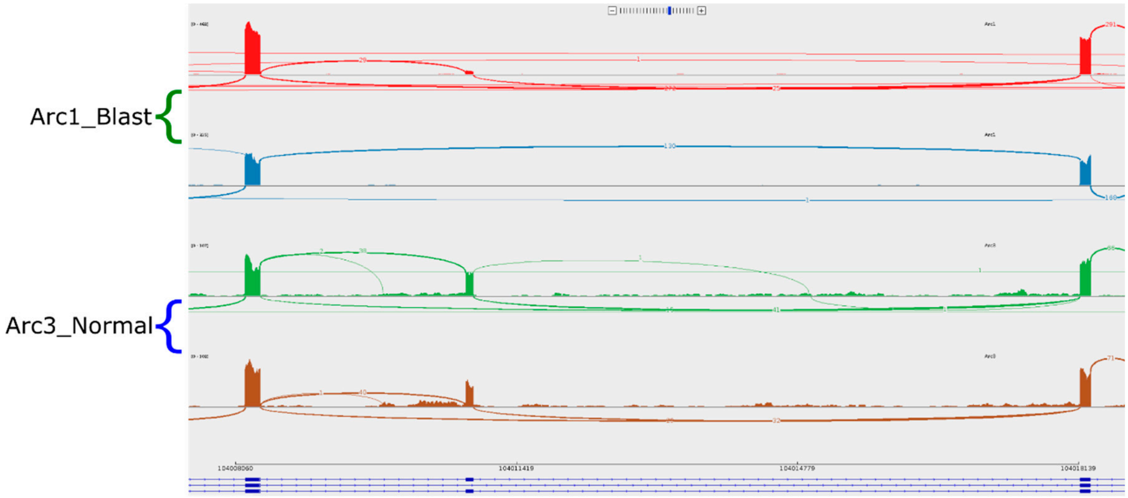

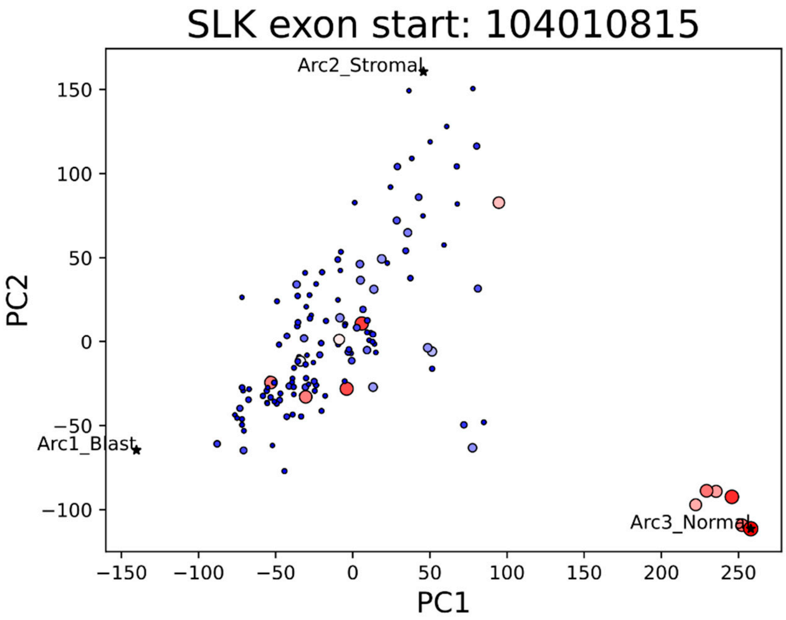

Figure S50: The gene SLK is alternatively spliced between samples located in different regions of latent space.

Top: A Shashimi plot of representative samples located near different archetypes. Bottom: a PCA plot of tumors and normal kidney samples, where each sample is marked according to the inclusion level of a selected mRNA isoform (large red – high, small blue – low). These results are consistent with previous observations that exon skipping in this gene in human mammary epithelial cells is associated with EMT and occurs when QKI and RBFOX1 are overexpressed [7].

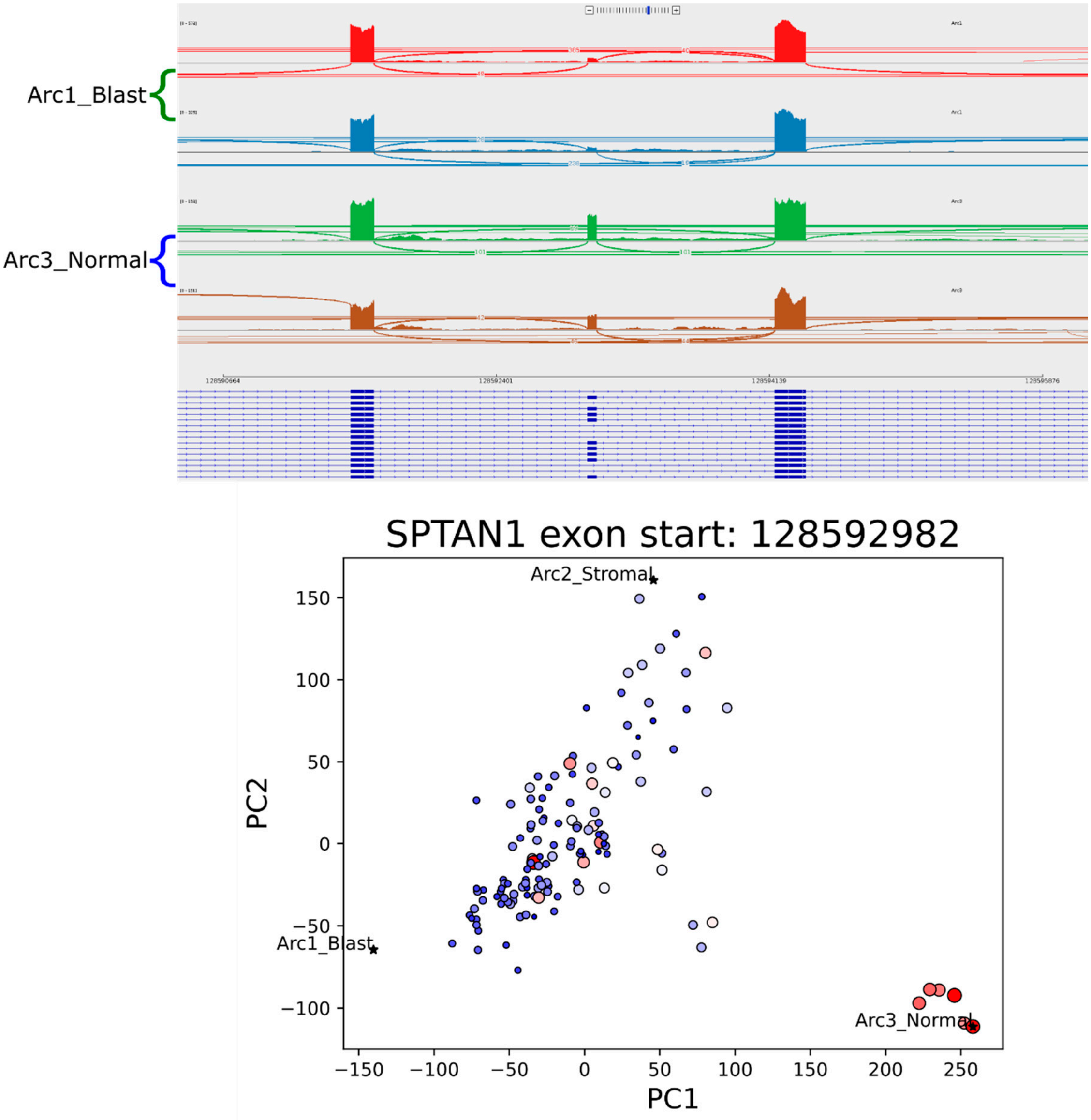

Figure S51: The gene SPTAN1 is alternatively spliced between samples located in different regions of latent space.

Top: A Shashimi plot of representative samples located near different archetypes. Bottom: a PCA plot of tumors and normal kidney samples, where each sample is marked according to the inclusion level of a selected mRNA isoform (large red – high, small blue – low).

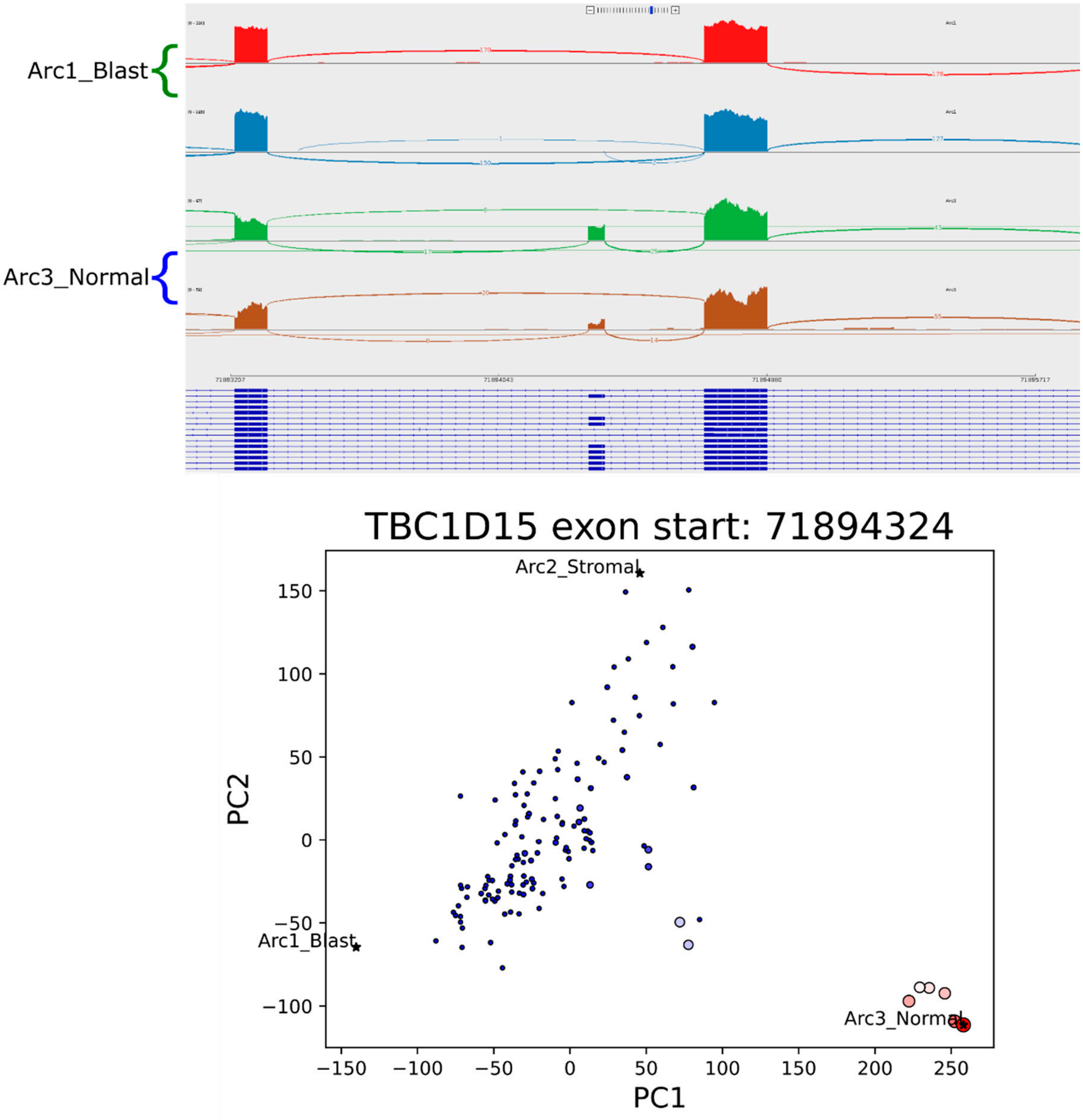

Figure S52: The gene TBC1D15 is alternatively spliced between samples located in different regions of latent space.

Top: A Shashimi plot of representative samples located near different archetypes. Bottom: a PCA plot of tumors and normal kidney samples, where each sample is marked according to the inclusion level of a selected mRNA isoform (large red – high, small blue – low).

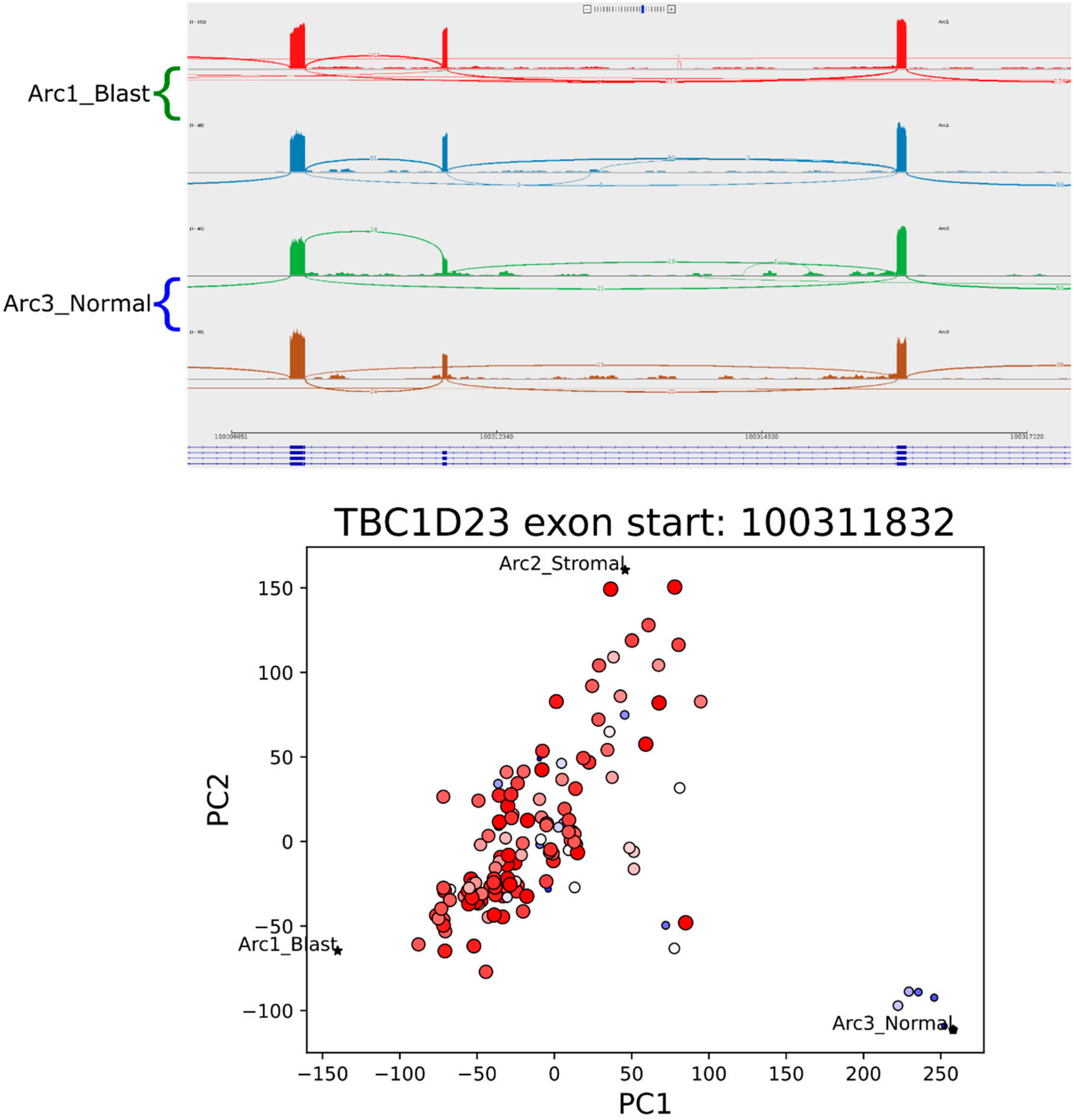

Figure S53: The gene TBC1D23 is alternatively spliced between samples located in different regions of latent space.

Top: A Shashimi plot of representative samples located near different archetypes. Bottom: a PCA plot of tumors and normal kidney samples, where each sample is marked according to the inclusion level of a selected mRNA isoform (large red – high, small blue – low).

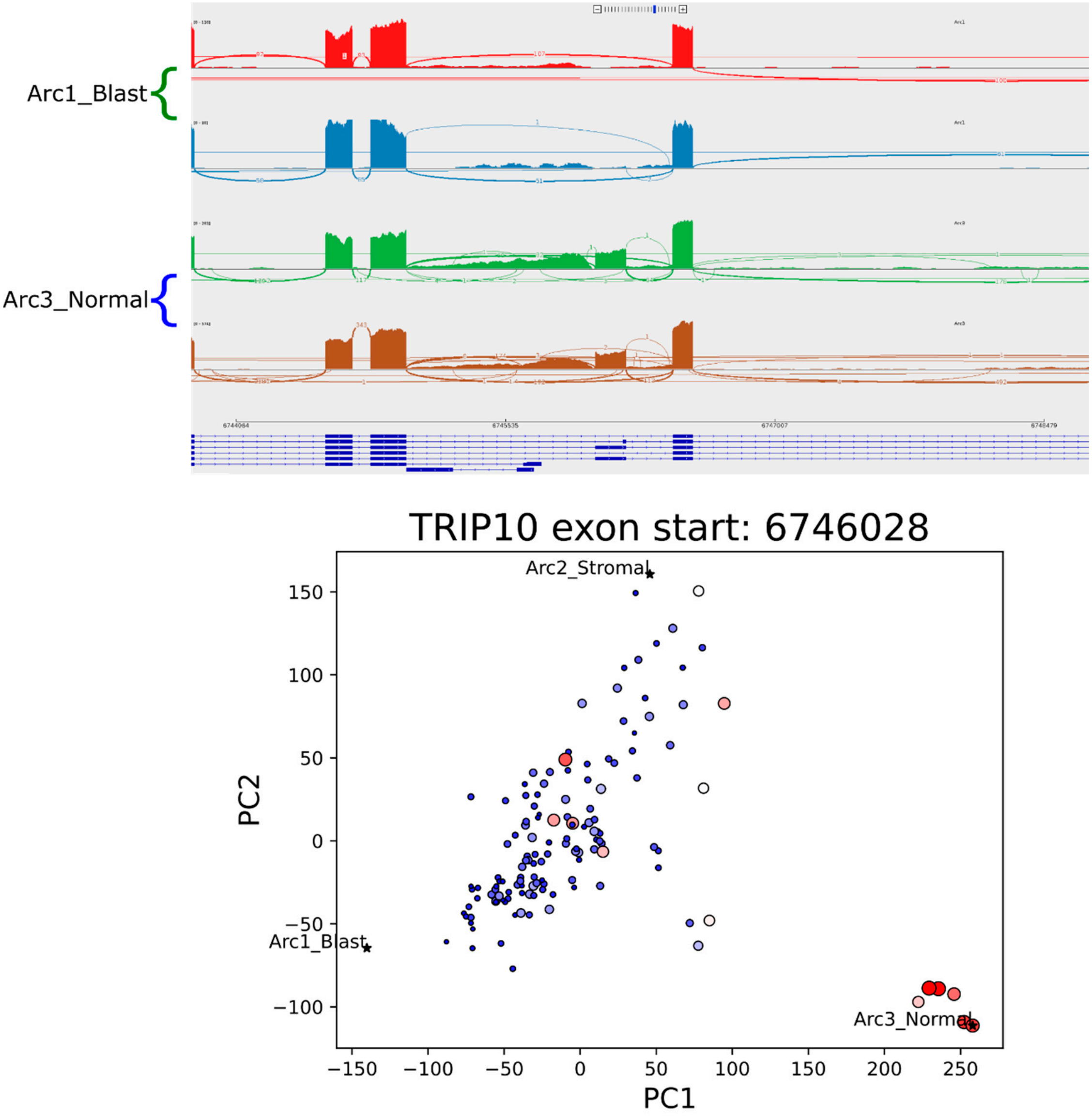

Figure S54: The gene TRIP10 is alternatively spliced between samples located in different regions of latent space.

Top: A Shashimi plot of representative samples located near different archetypes. Bottom: a PCA plot of tumors and normal kidney samples, where each sample is marked according to the inclusion level of a selected mRNA isoform (large red – high, small blue – low).

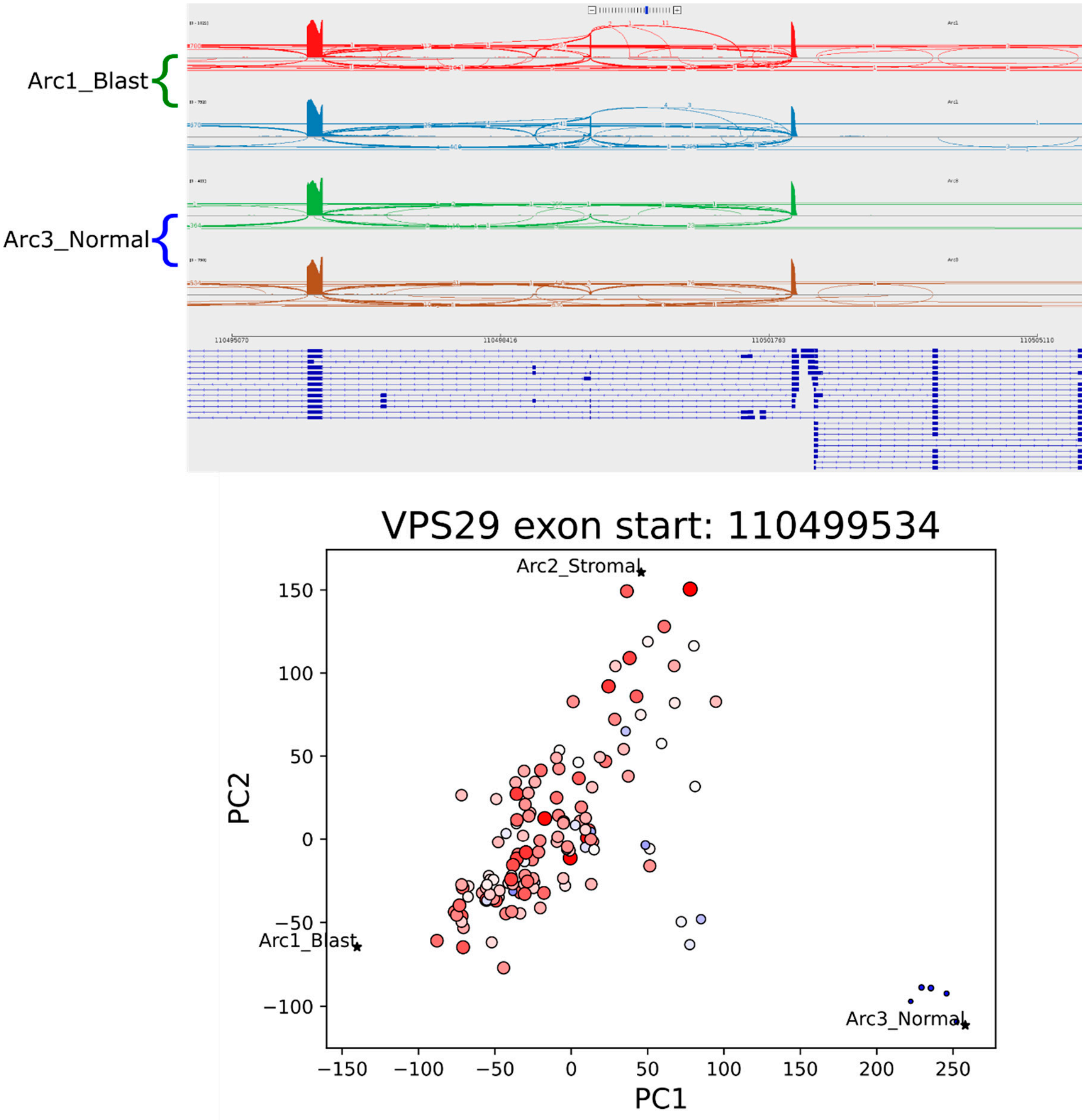

Figure S55: The gene VPS29 is alternatively spliced between samples located in different regions of latent space.

Top: A Shashimi plot of representative samples located near different archetypes. Bottom: a PCA plot of tumors and normal kidney samples, where each sample is marked according to the inclusion level of a selected mRNA isoform (large red – high, small blue – low).

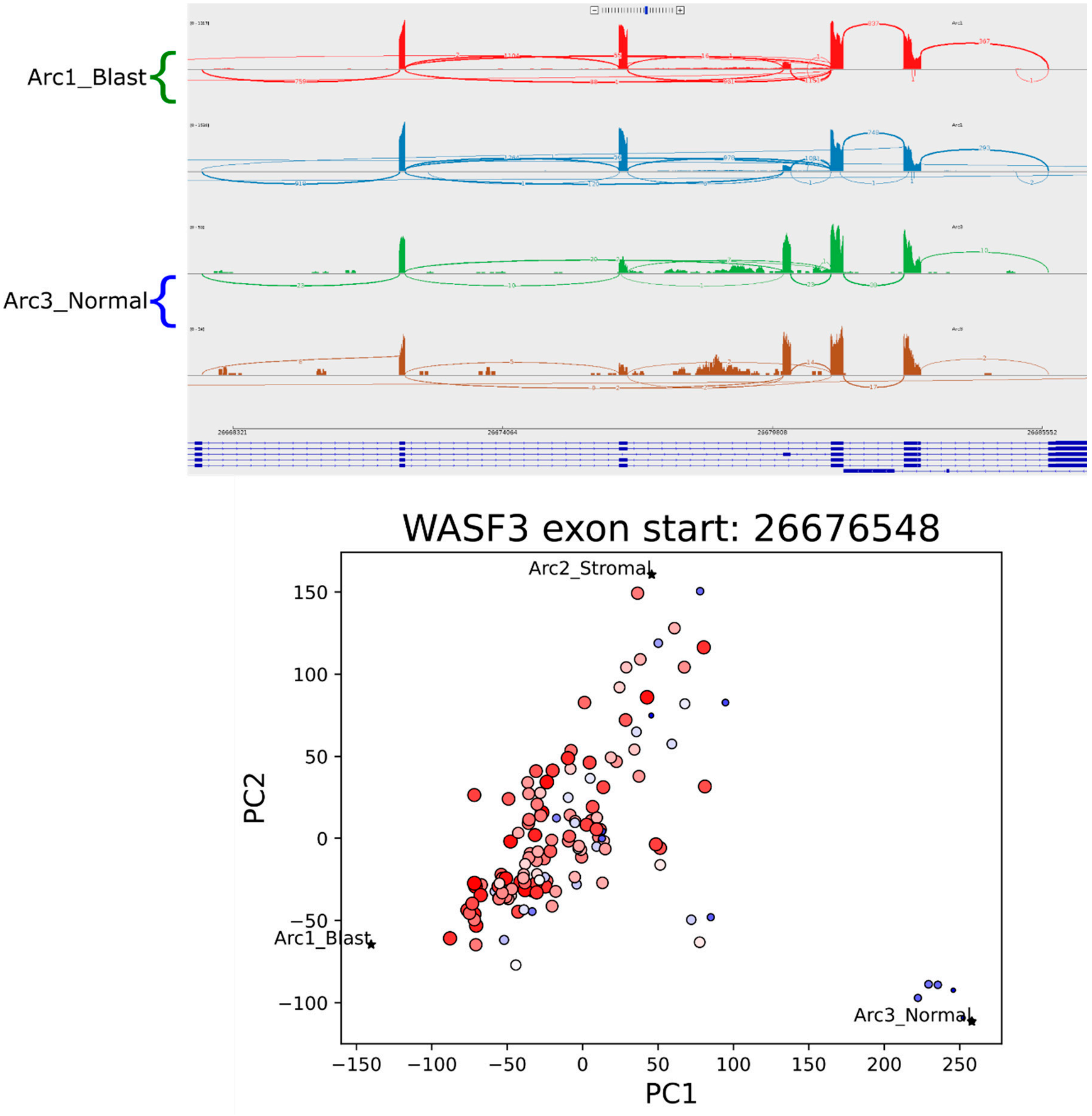

Figure S56: The gene WASF3 is alternatively spliced between samples located in different regions of latent space.

Top: A Shashimi plot of representative samples located near different archetypes. Bottom: a PCA plot of tumors and normal kidney samples, where each sample is marked according to the inclusion level of a selected mRNA isoform (large red – high, small blue – low).

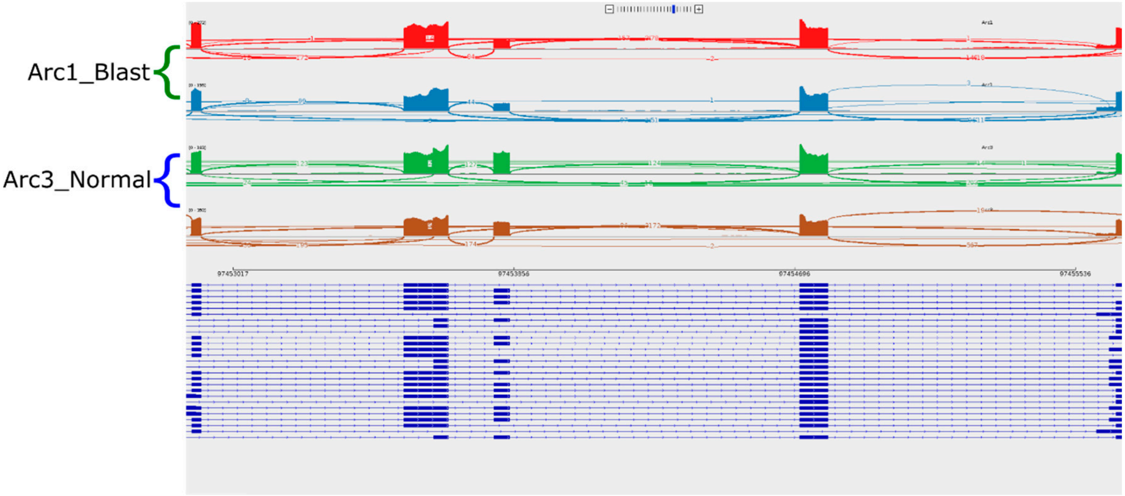

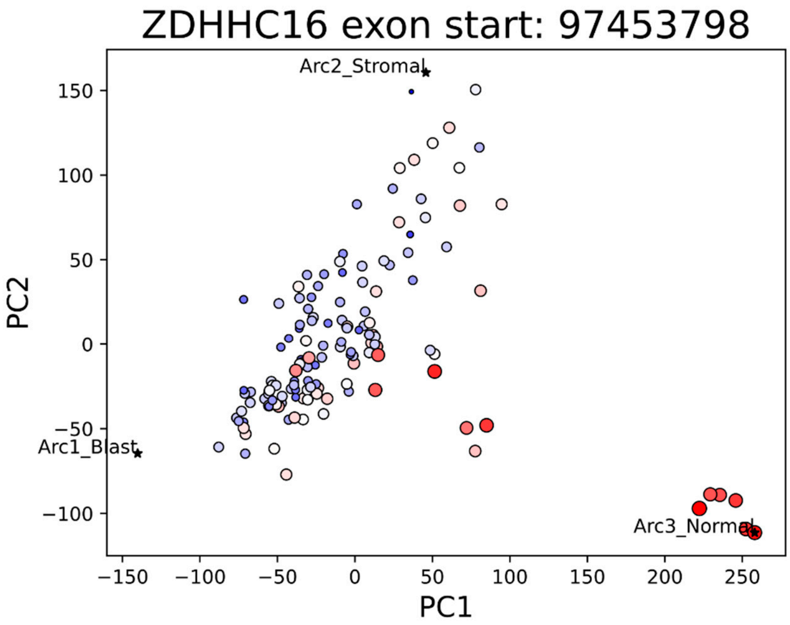

Figure S57: The gene ZDHHC16 is alternatively spliced between samples located in different regions of latent space.

Top: A Shashimi plot of representative samples located near different archetypes. Bottom: a PCA plot of tumors and normal kidney samples, where each sample is marked according to the inclusion level of a selected mRNA isoform (large red – high, small blue – low).

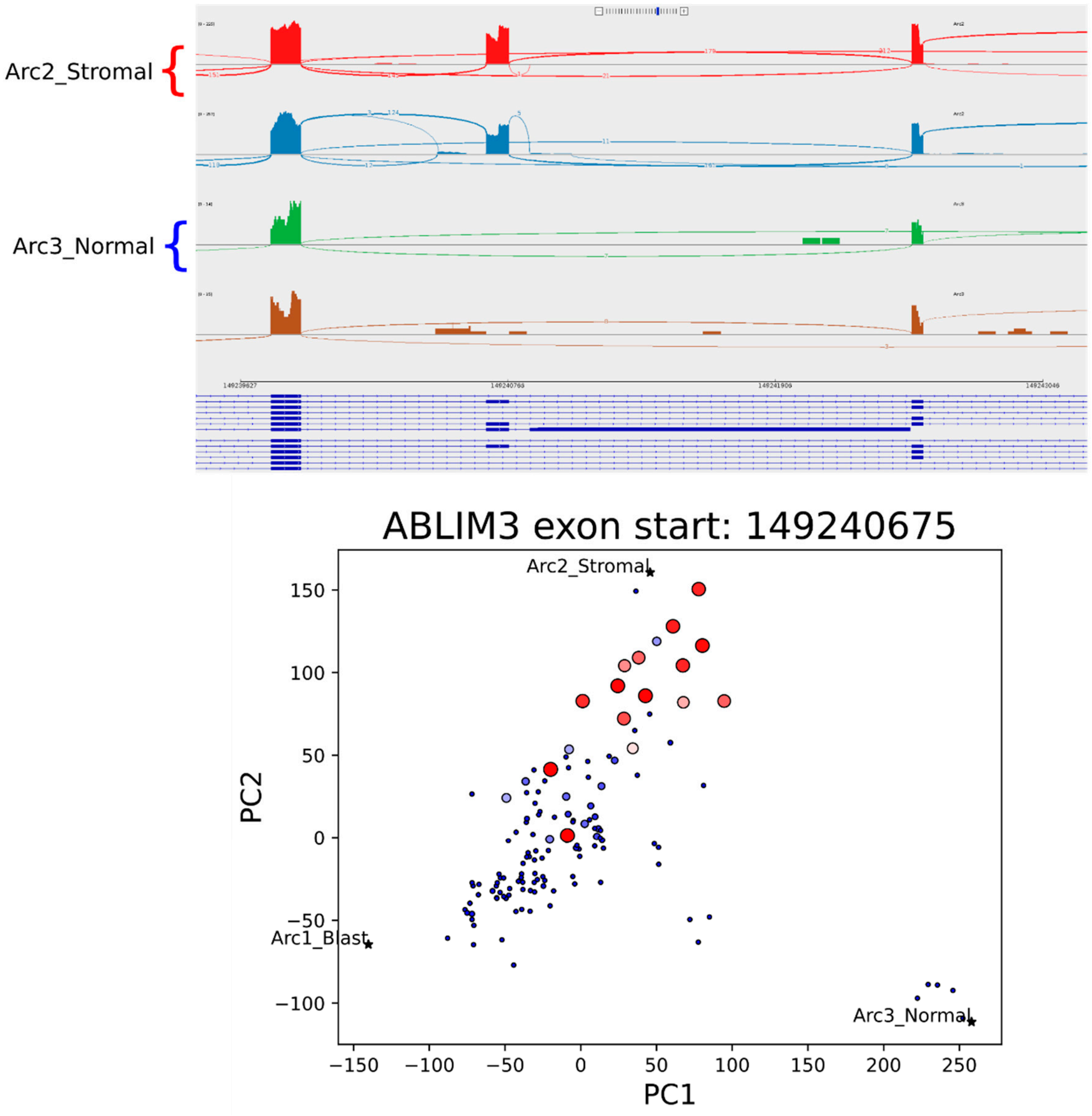

Figure S58: The gene ABLIM3 is alternatively spliced between samples located in different regions of latent space.

Top: A Shashimi plot of representative samples located near different archetypes. Bottom: a PCA plot of tumors and normal kidney samples, where each sample is marked according to the inclusion level of a selected mRNA isoform (large red – high, small blue – low).

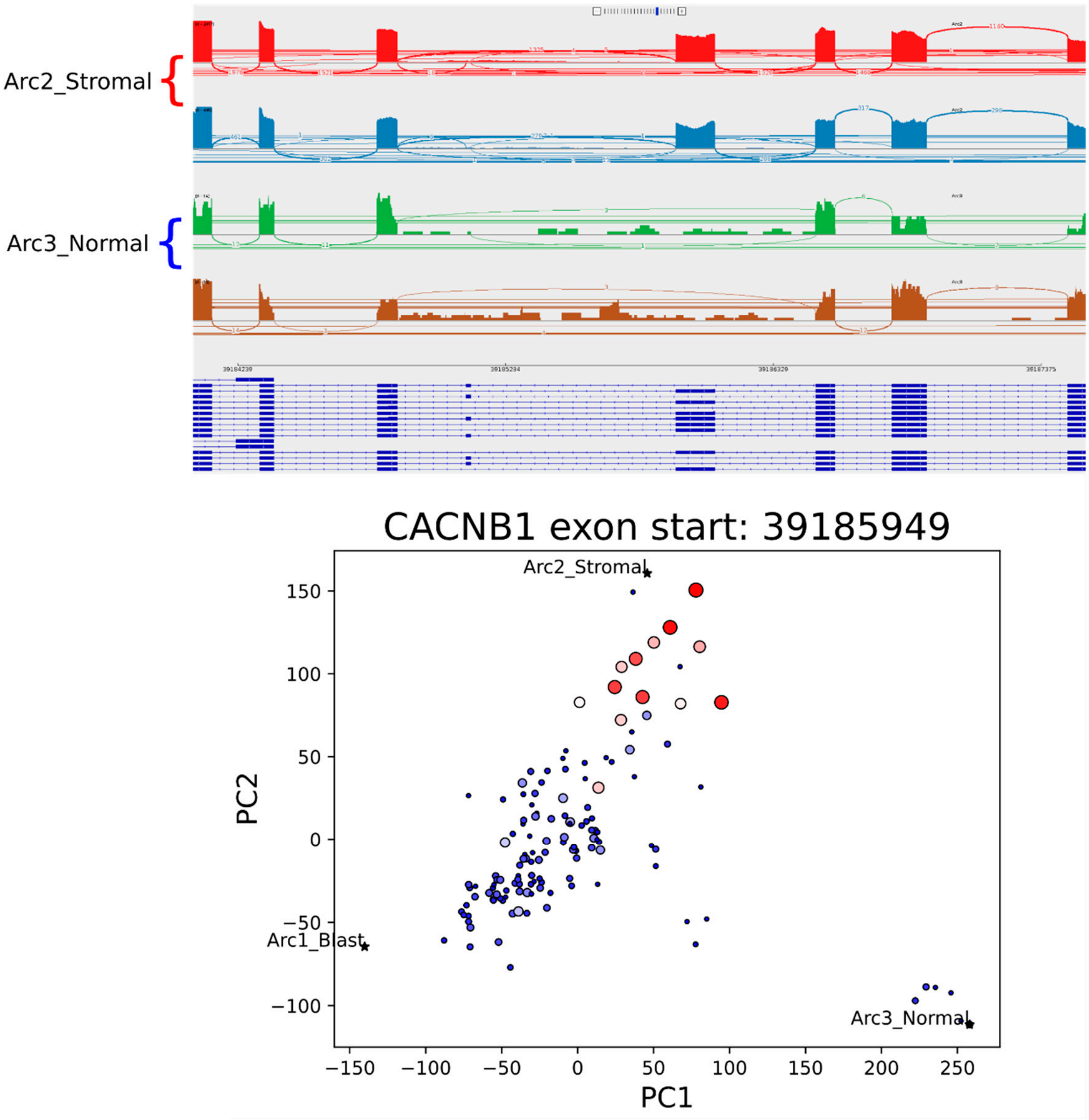

Figure S59: The gene CACNB1 is alternatively spliced between samples located in different regions of latent space.

Top: A Shashimi plot of representative samples located near different archetypes. Bottom: a PCA plot of tumors and normal kidney samples, where each sample is marked according to the inclusion level of a selected mRNA isoform (large red – high, small blue – low).

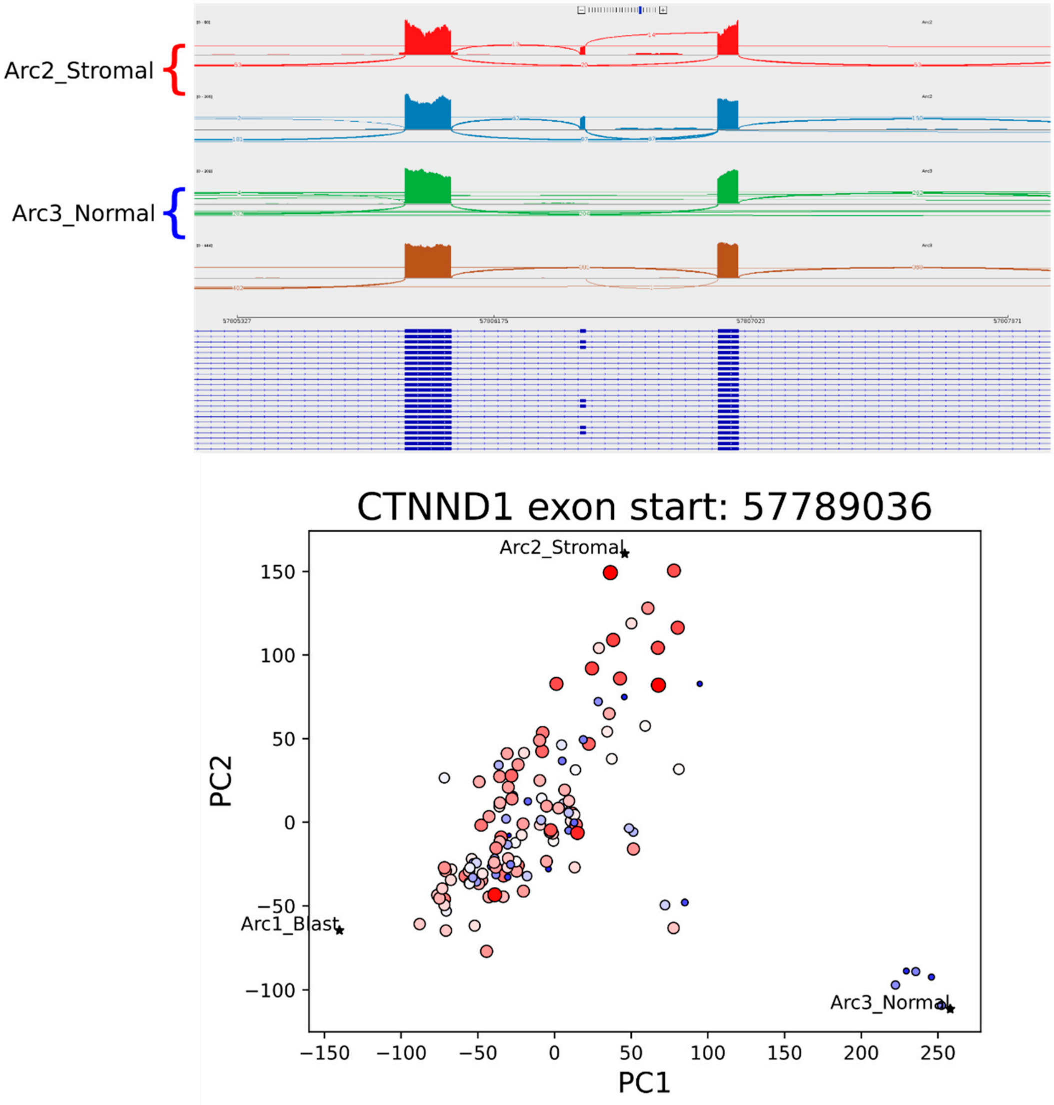

Figure S60: The gene CTNND1 is alternatively spliced between samples located in different regions of latent space.

Top: A Shashimi plot of representative samples located near different archetypes. Bottom: a PCA plot of tumors and normal kidney samples, where each sample is marked according to the inclusion level of a selected mRNA isoform (large red – high, small blue – low).

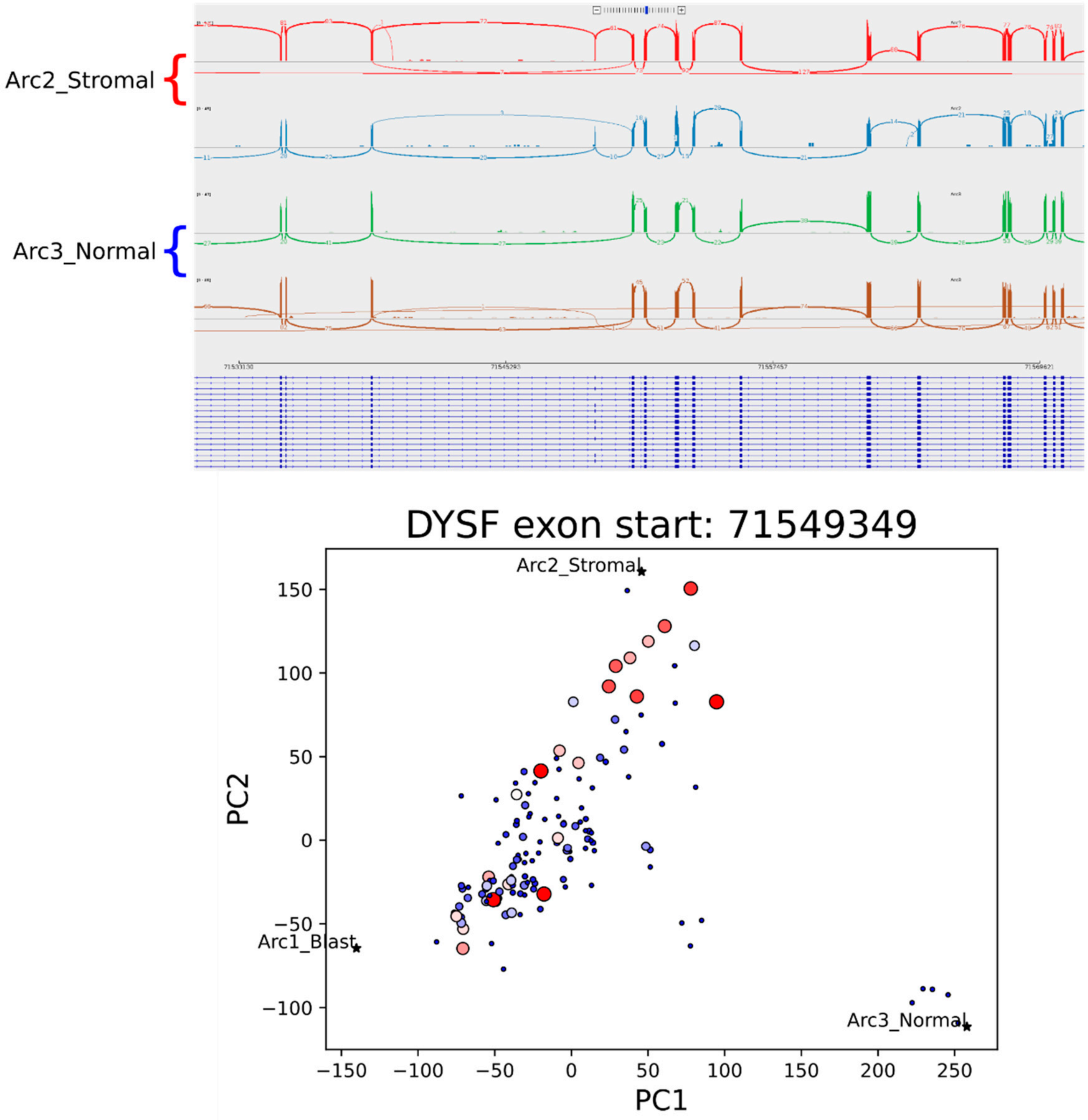

Figure S61: The gene DYSF is alternatively spliced between samples located in different regions of latent space.

Top: A Shashimi plot of representative samples located near different archetypes. Bottom: a PCA plot of tumors and normal kidney samples, where each sample is marked according to the inclusion level of a selected mRNA isoform (large red – high, small blue – low).

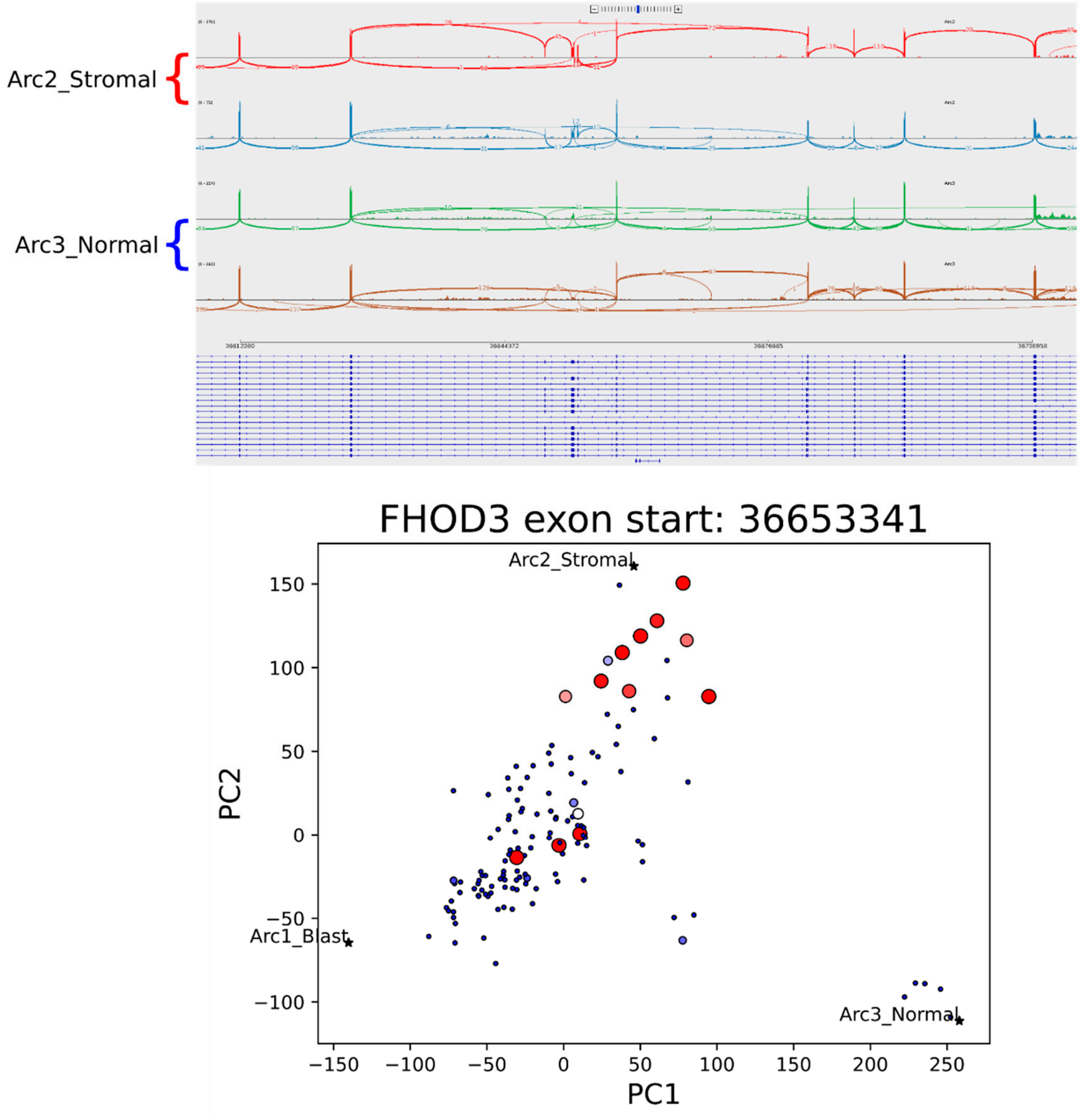

Figure S62: The gene FHOD3 is alternatively spliced between samples located in different regions of latent space.

Top: A Shashimi plot of representative samples located near different archetypes. Bottom: a PCA plot of tumors and normal kidney samples, where each sample is marked according to the inclusion level of a selected mRNA isoform (large red – high, small blue – low).

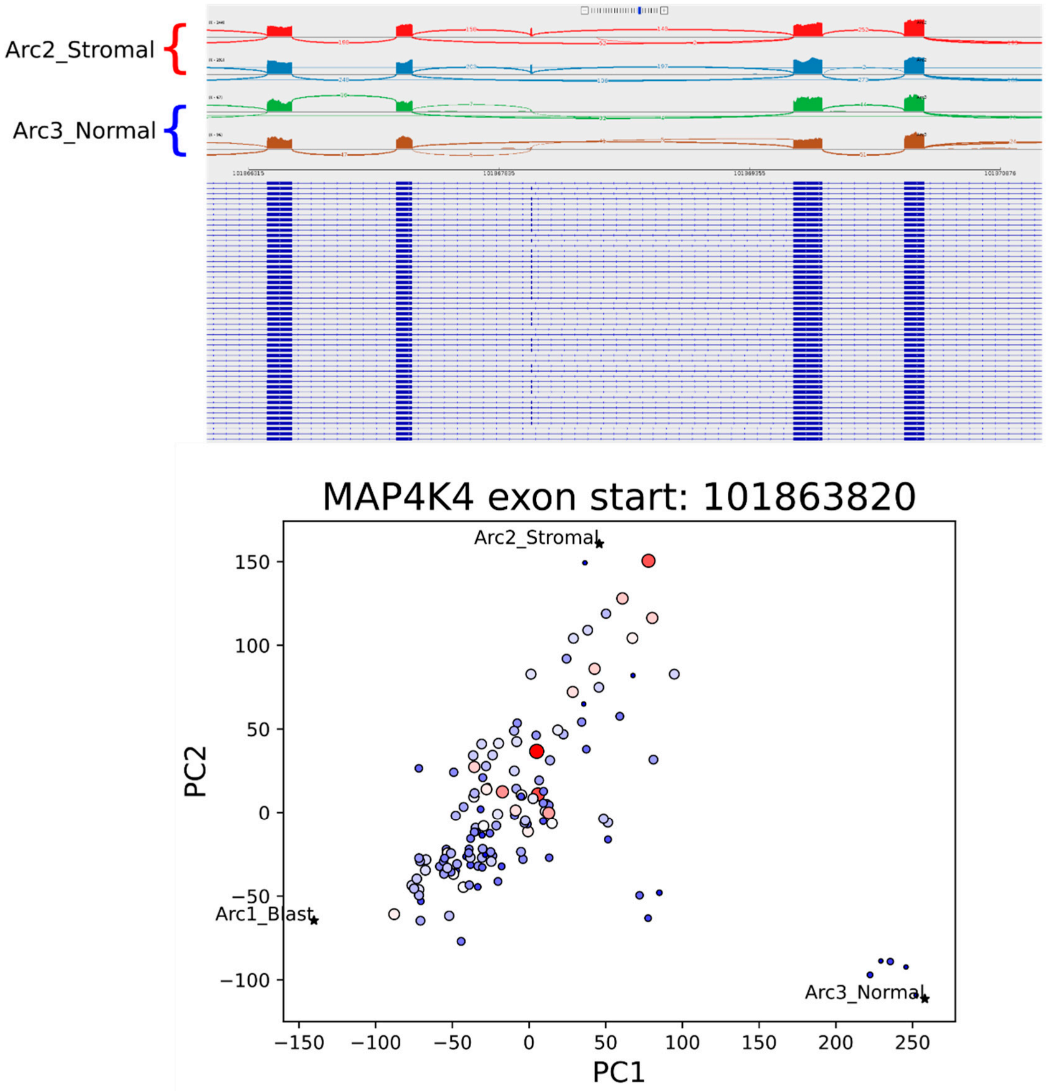

Figure S63: The gene MAP4K4 is alternatively spliced between samples located in different regions of latent space.

Top: A Shashimi plot of representative samples located near different archetypes. Bottom: a PCA plot of tumors and normal kidney samples, where each sample is marked according to the inclusion level of a selected mRNA isoform (large red – high, small blue – low).

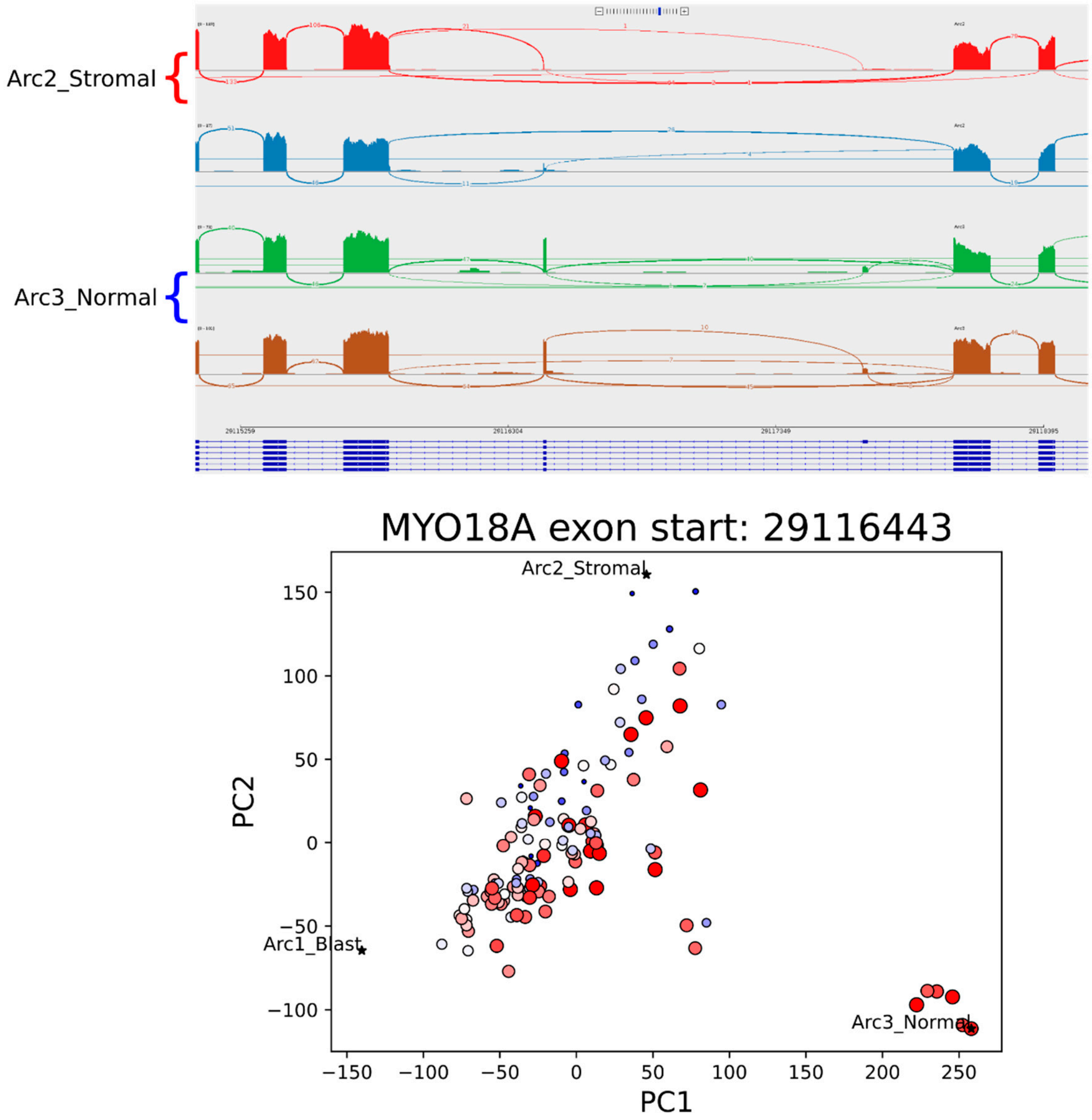

Figure S64: The gene MYO18A is alternatively spliced between samples located in different regions of latent space.

Top: A Shashimi plot of representative samples located near different archetypes. Bottom: a PCA plot of tumors and normal kidney samples, where each sample is marked according to the inclusion level of a selected mRNA isoform (large red – high, small blue – low). This gene is known to have muscle-specific transcripts that are dependent on the splicing regulator RBFOX2 [3].

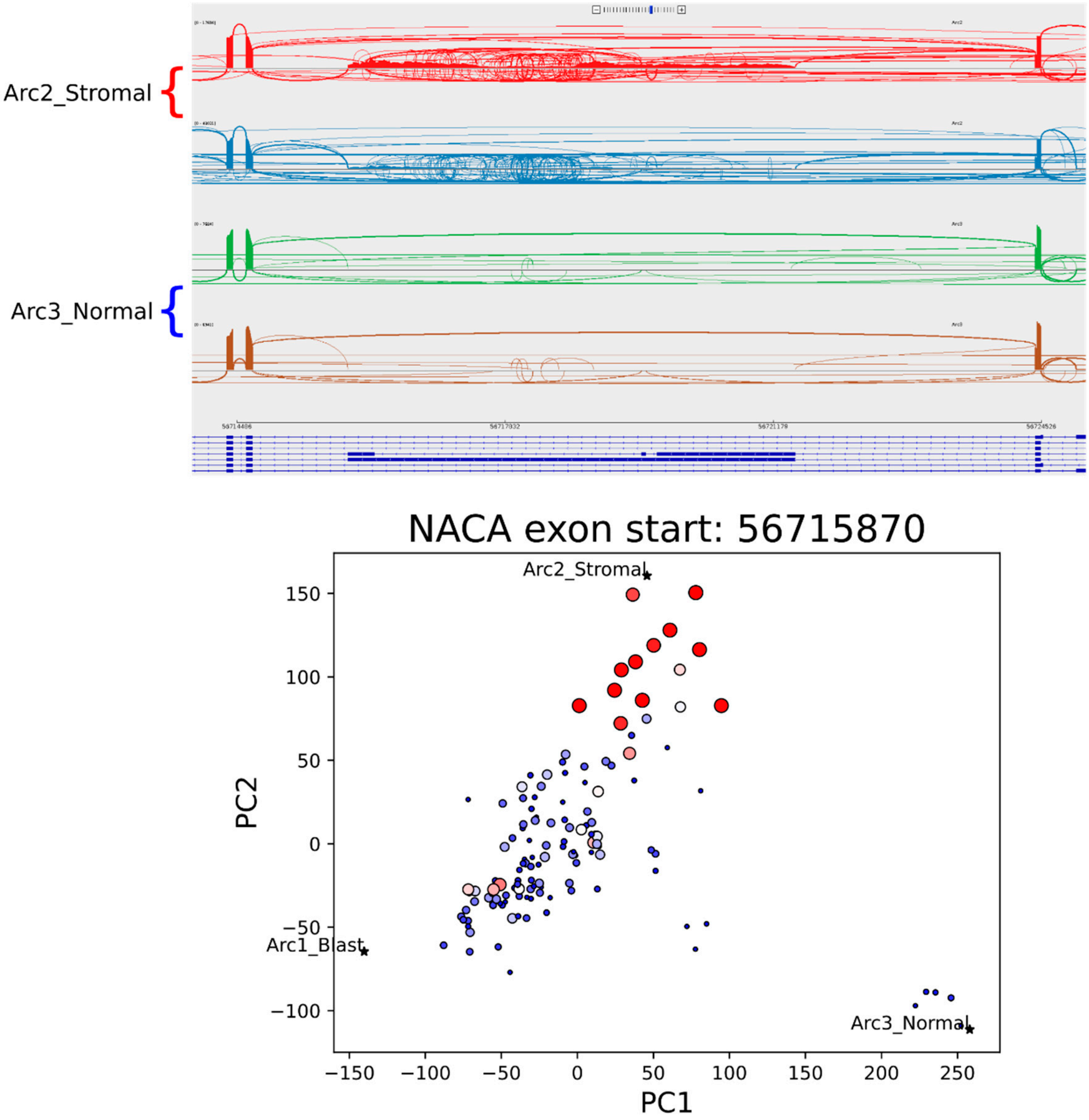

Figure S65: The gene NACA is alternatively spliced between samples located in different regions of latent space.

Top: A Shashimi plot of representative samples located near different archetypes. Bottom: a PCA plot of tumors and normal kidney samples, where each sample is marked according to the inclusion level of a selected mRNA isoform (large red – high, small blue – low).

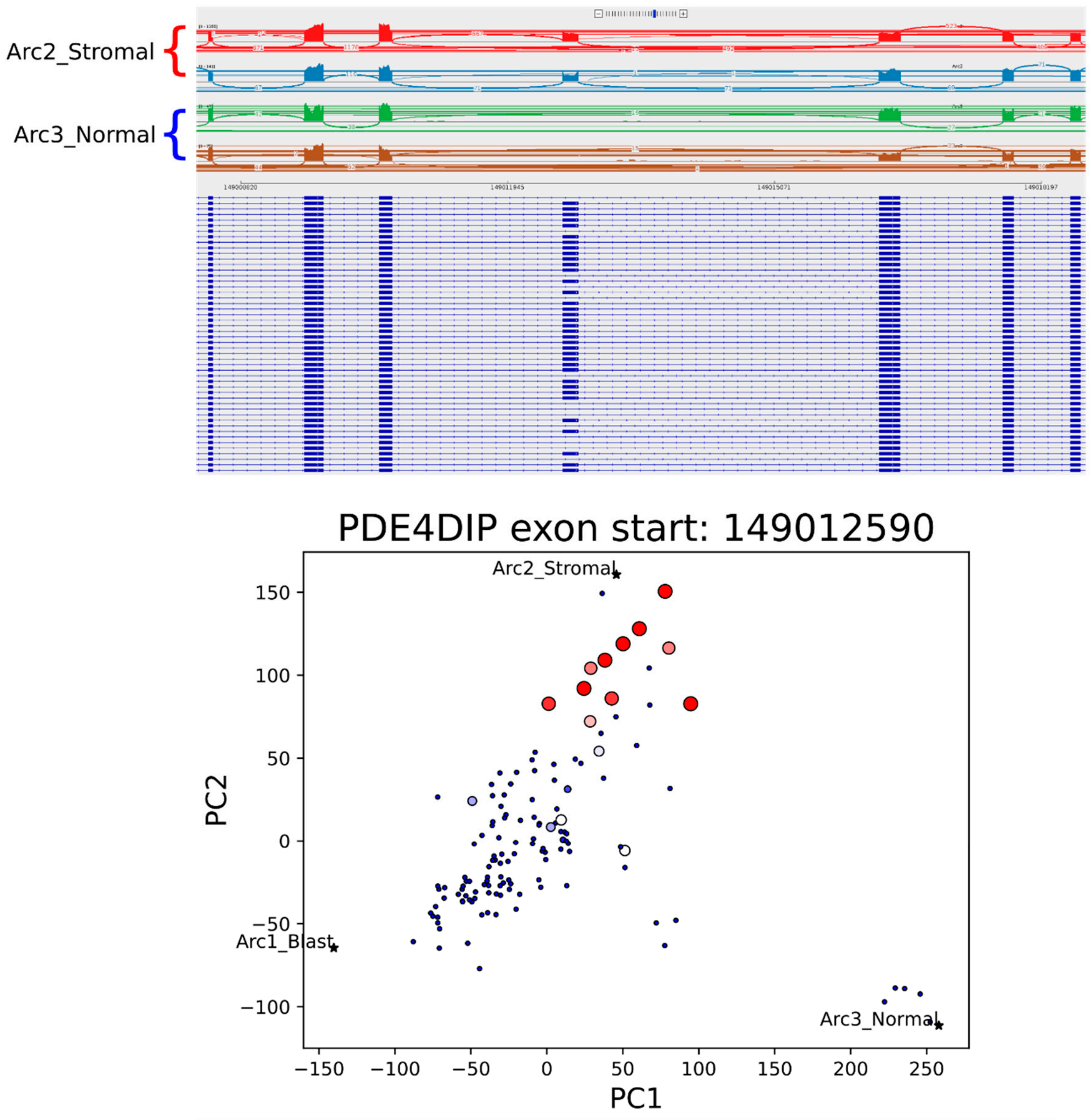

Figure S66: The gene PDE4DIP is alternatively spliced between samples located in different regions of latent space.

Top: A Shashimi plot of representative samples located near different archetypes. Bottom: a PCA plot of tumors and normal kidney samples, where each sample is marked according to the inclusion level of a selected mRNA isoform (large red – high, small blue – low).

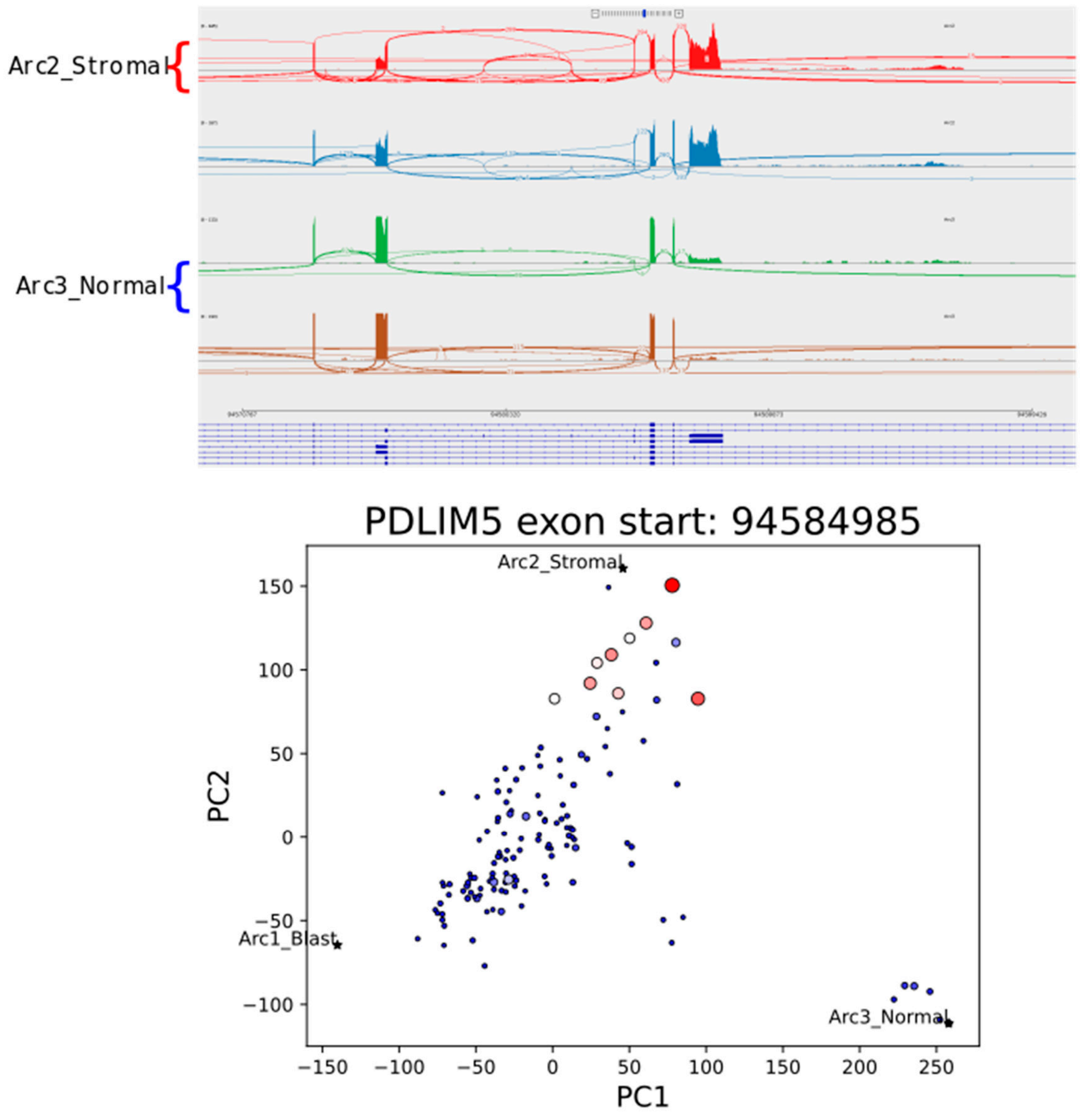

Figure S67: The gene PDLIM5 is alternatively spliced between samples located in different regions of latent space.

Top: A Shashimi plot of representative samples located near different archetypes. Bottom: a PCA plot of tumors and normal kidney samples, where each sample is marked according to the inclusion level of a selected mRNA isoform (large red – high, small blue – low).

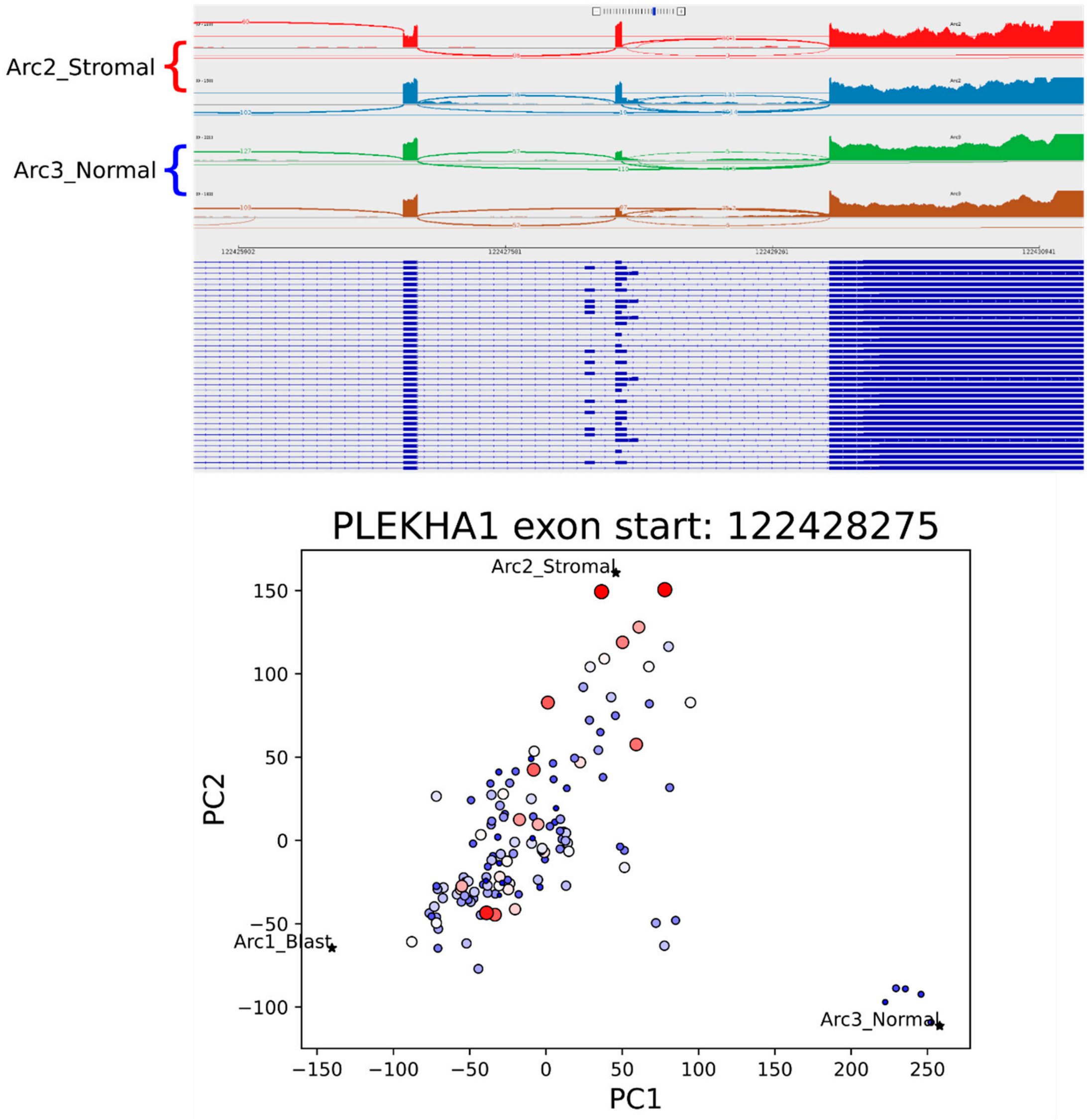

Figure S68: The gene PLEKHA1 is alternatively spliced between samples located in different regions of latent space.

Top: A Shashimi plot of representative samples located near different archetypes. Bottom: a PCA plot of tumors and normal kidney samples, where each sample is marked according to the inclusion level of a selected mRNA isoform (large red – high, small blue – low).

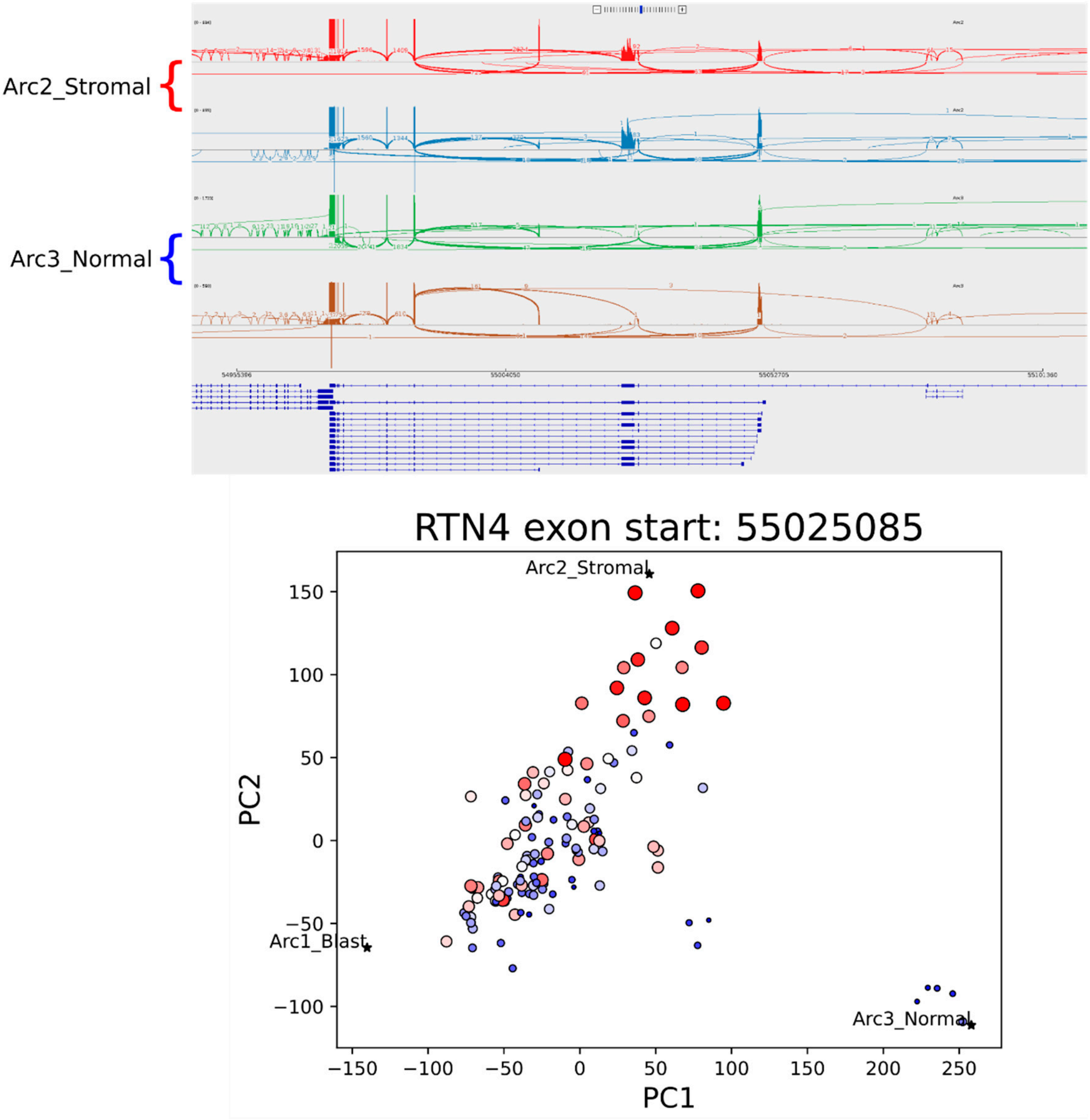

Figure S69: The gene RTN4 is alternatively spliced between samples located in different regions of latent space.

Top: A Shashimi plot of representative samples located near different archetypes. Bottom: a PCA plot of tumors and normal kidney samples, where each sample is marked according to the inclusion level of a selected mRNA isoform (large red – high, small blue – low).

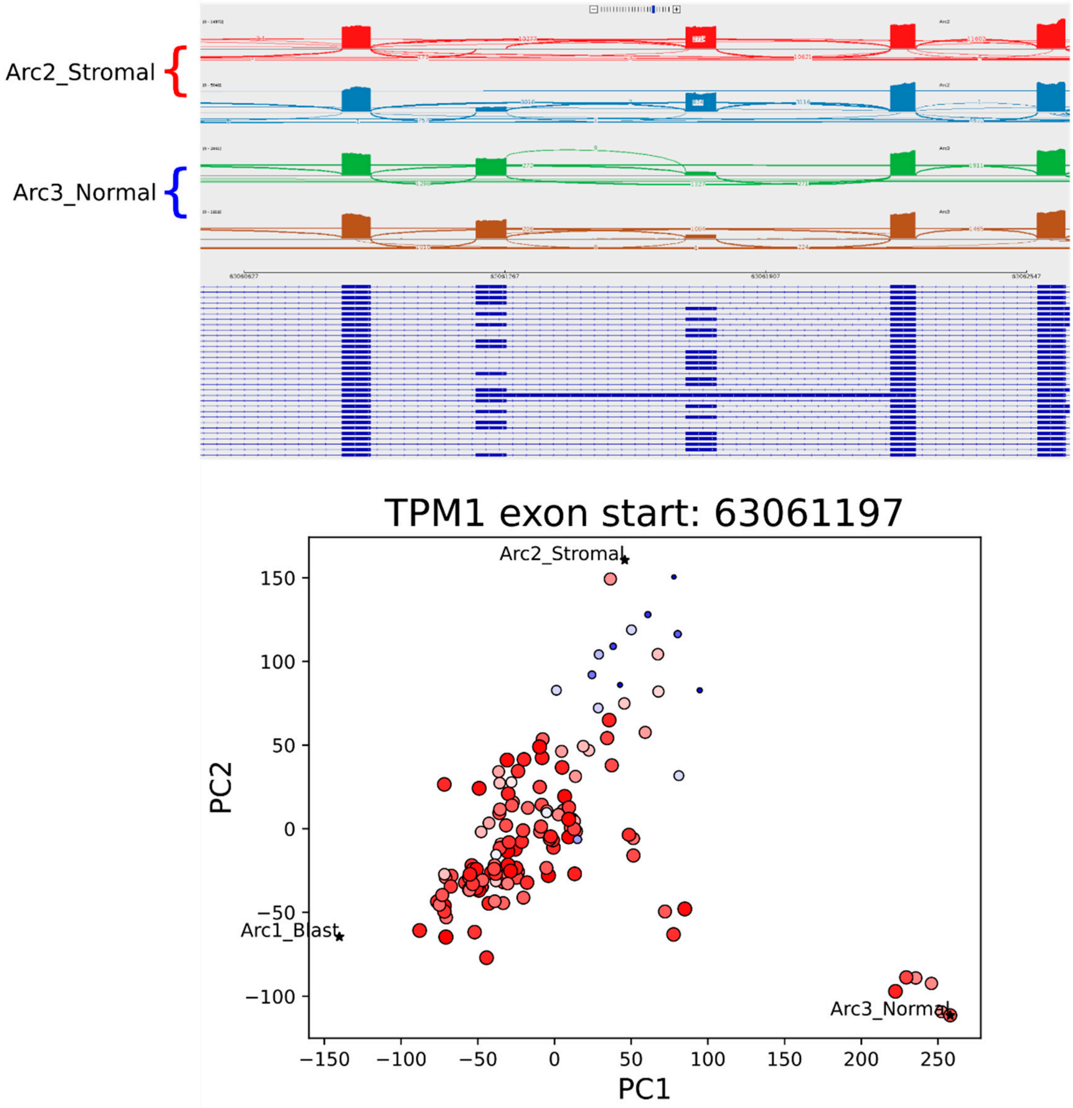

Figure S70: The gene TPM1 is alternatively spliced between samples located in different regions of latent space.

Top: A Shashimi plot of representative samples located near different archetypes. Bottom: a PCA plot of tumors and normal kidney samples, where each sample is marked according to the inclusion level of a selected mRNA isoform (large red – high, small blue – low).

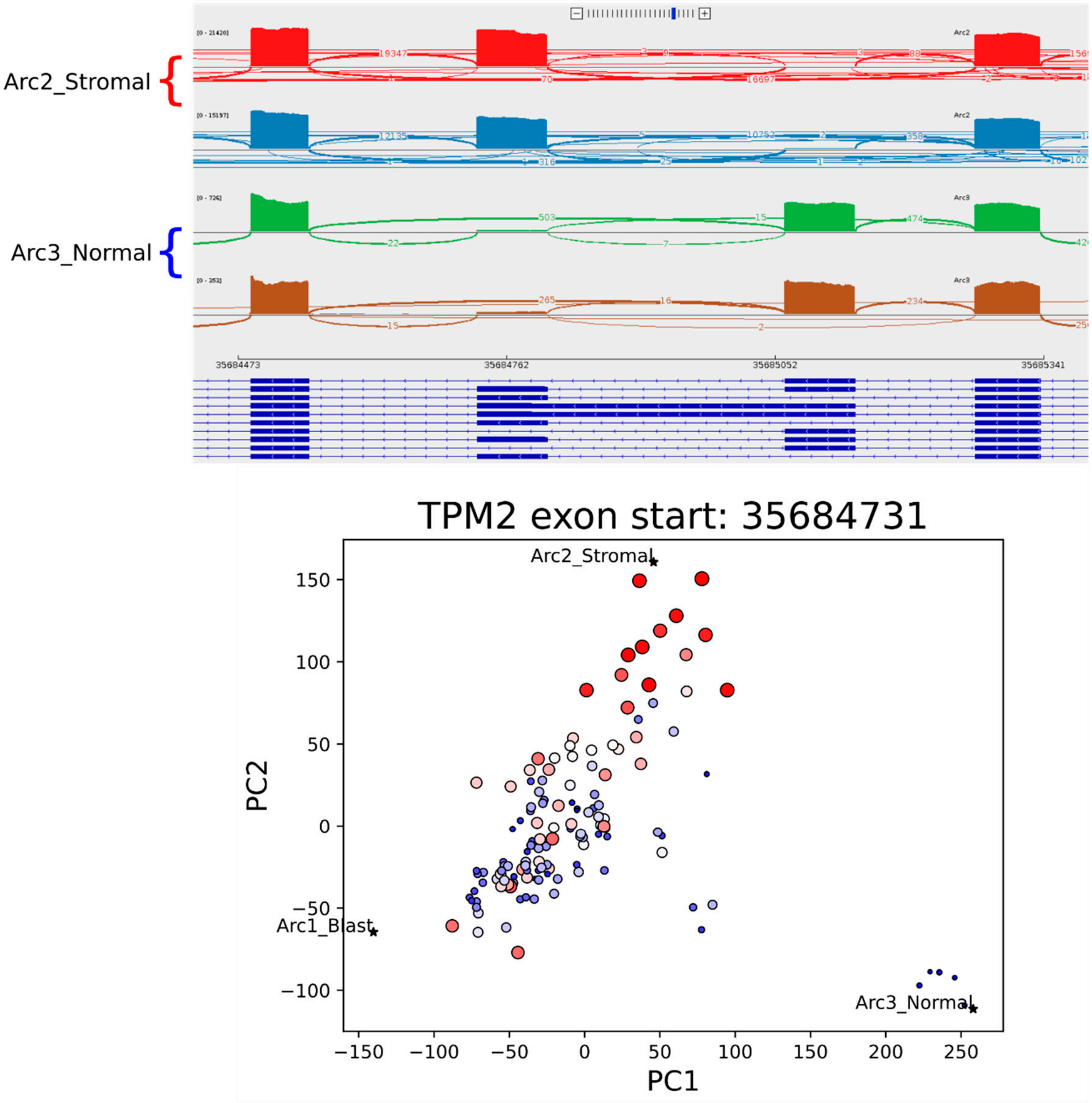

Figure S71: The gene TPM2 is alternatively spliced between samples located in different regions of latent space.

Top: A Shashimi plot of representative samples located near different archetypes. Bottom: a PCA plot of tumors and normal kidney samples, where each sample is marked according to the inclusion level of a selected mRNA isoform (large red – high, small blue – low).

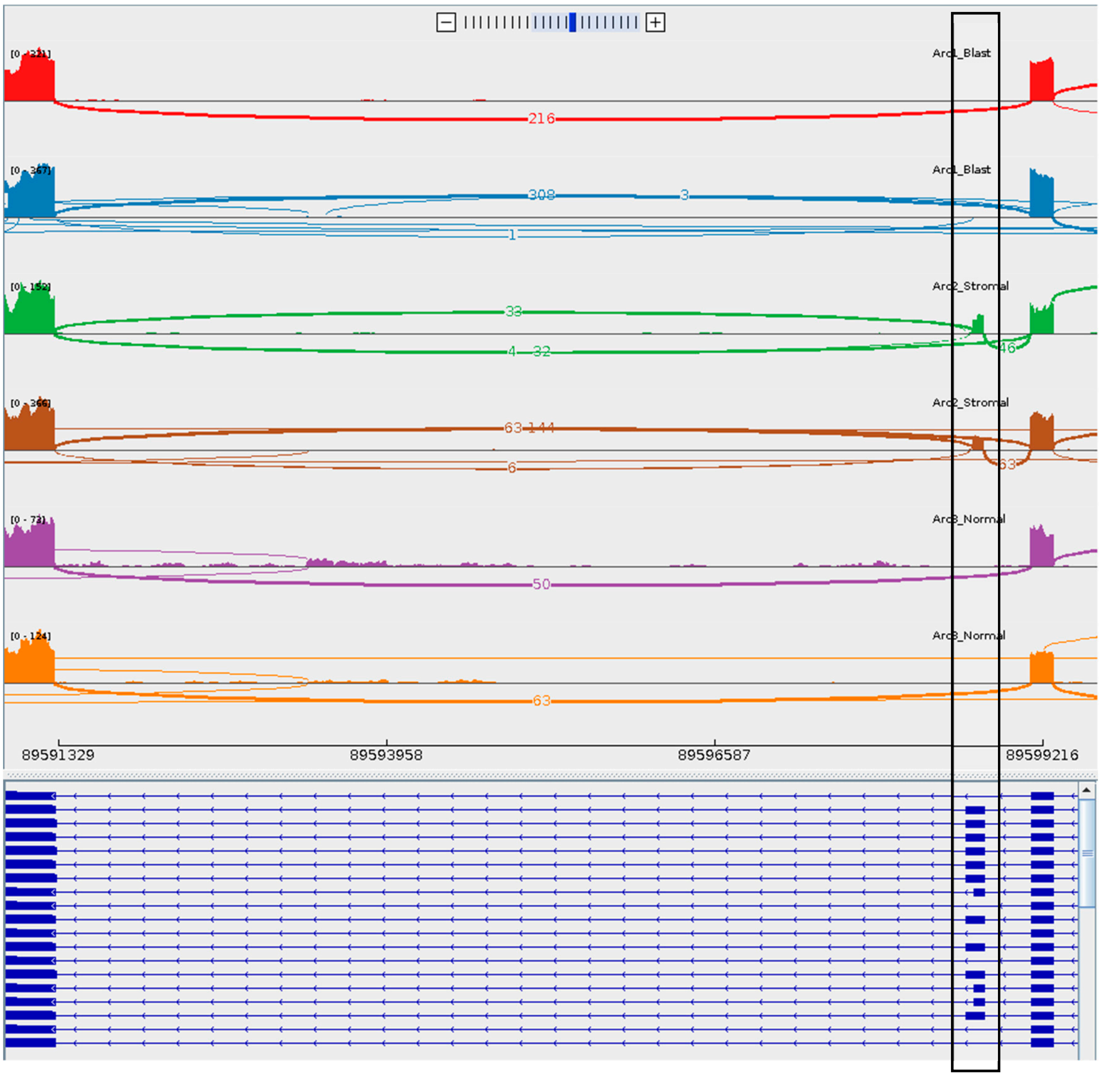

Figure S72: The gene ATP2B1 (PMCA1) is alternatively spliced between samples located in different regions of latent space.

Top: A Shashimi plot of representative samples located near different archetypes. The stromal tumors express transcripts from “splice variant c” of this gene [9], [10]. This isoform includes an extra exon (“site C”) and is over-expressed specifically in skeletal and heart muscle but not in kidney tissues.

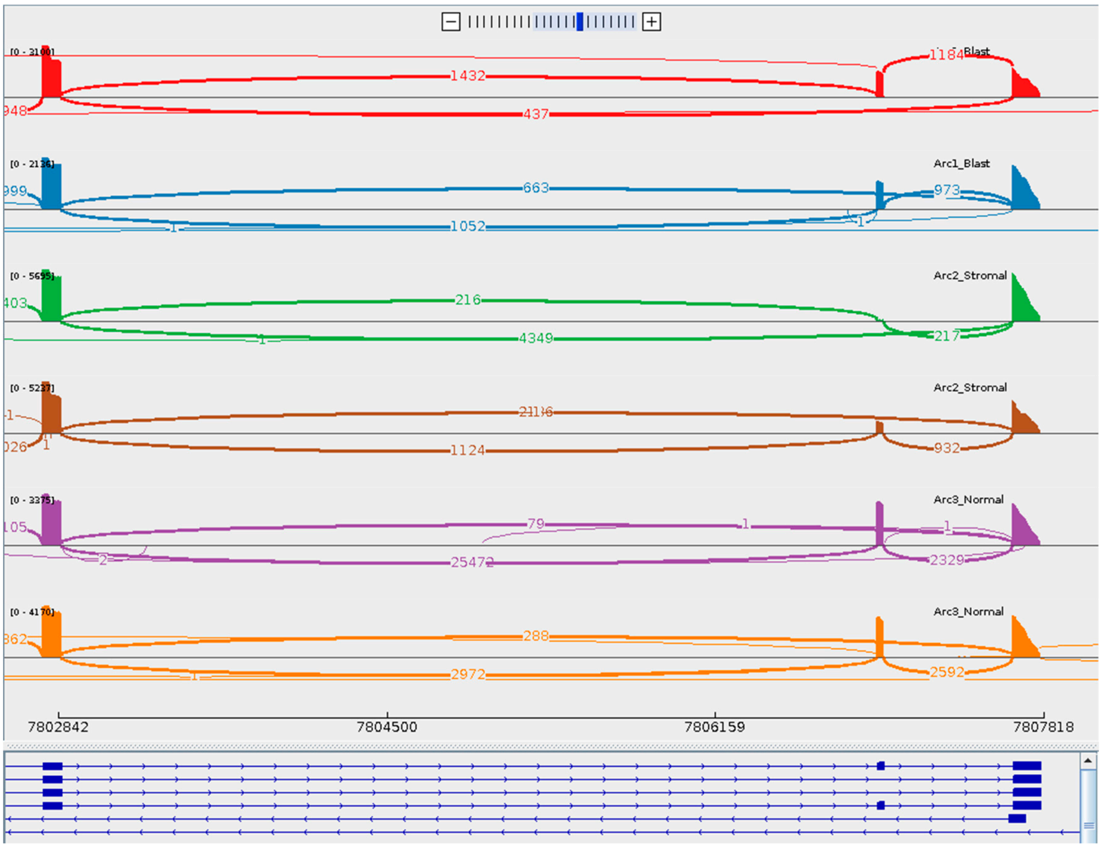

Figure S73: The gene ATP5F1C (ATP5C1) is alternatively spliced between samples located in different regions of latent space.

Top: A Shashimi plot of representative samples located near different archetypes. Previous observations showed that exon skipping in this gene in human mammary epithelial cells is associated with EMT and occurs when QKI and RBFOX1 are overexpressed [7].

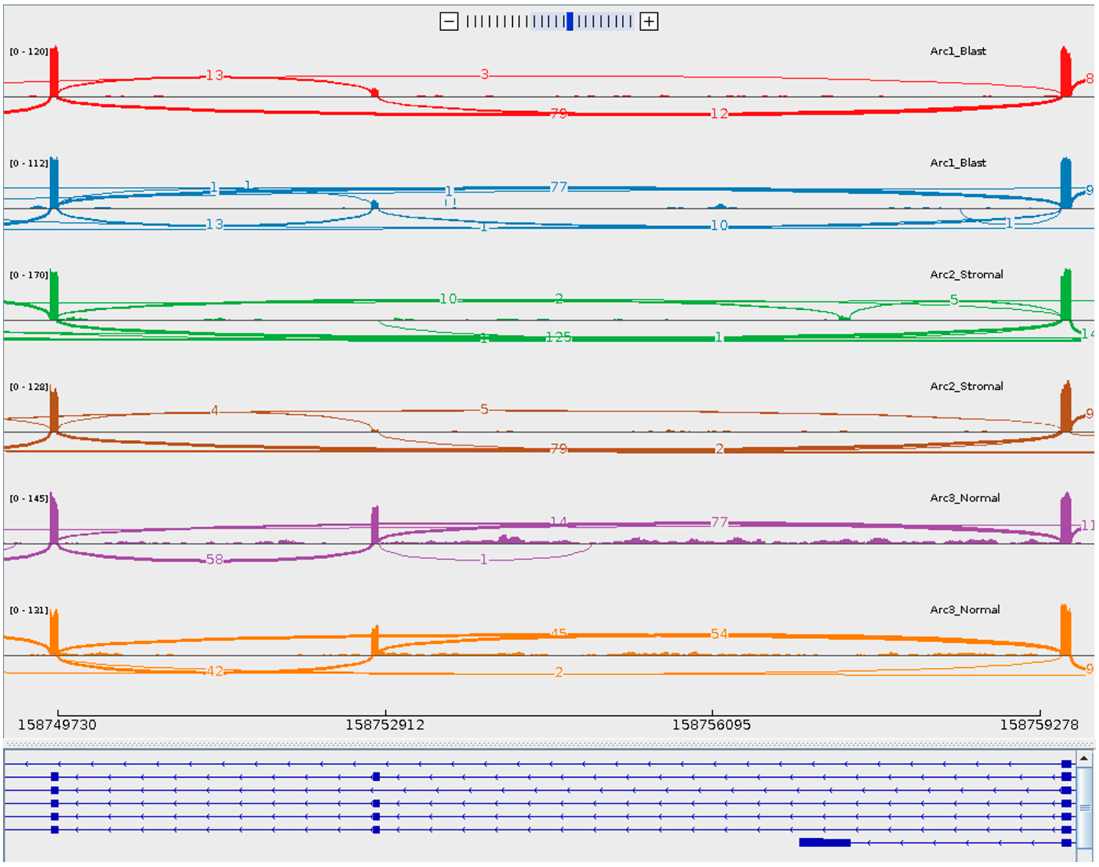

Figure S74: The gene ESYT2 is alternatively spliced between samples located in different regions of latent space.

Top: A Shashimi plot of representative samples located near different archetypes. These results are consistent with previous observations that exon skipping in this gene in human mammary epithelial cells is associated with EMT and occurs when QKI and RBFOX1 are overexpressed [7].

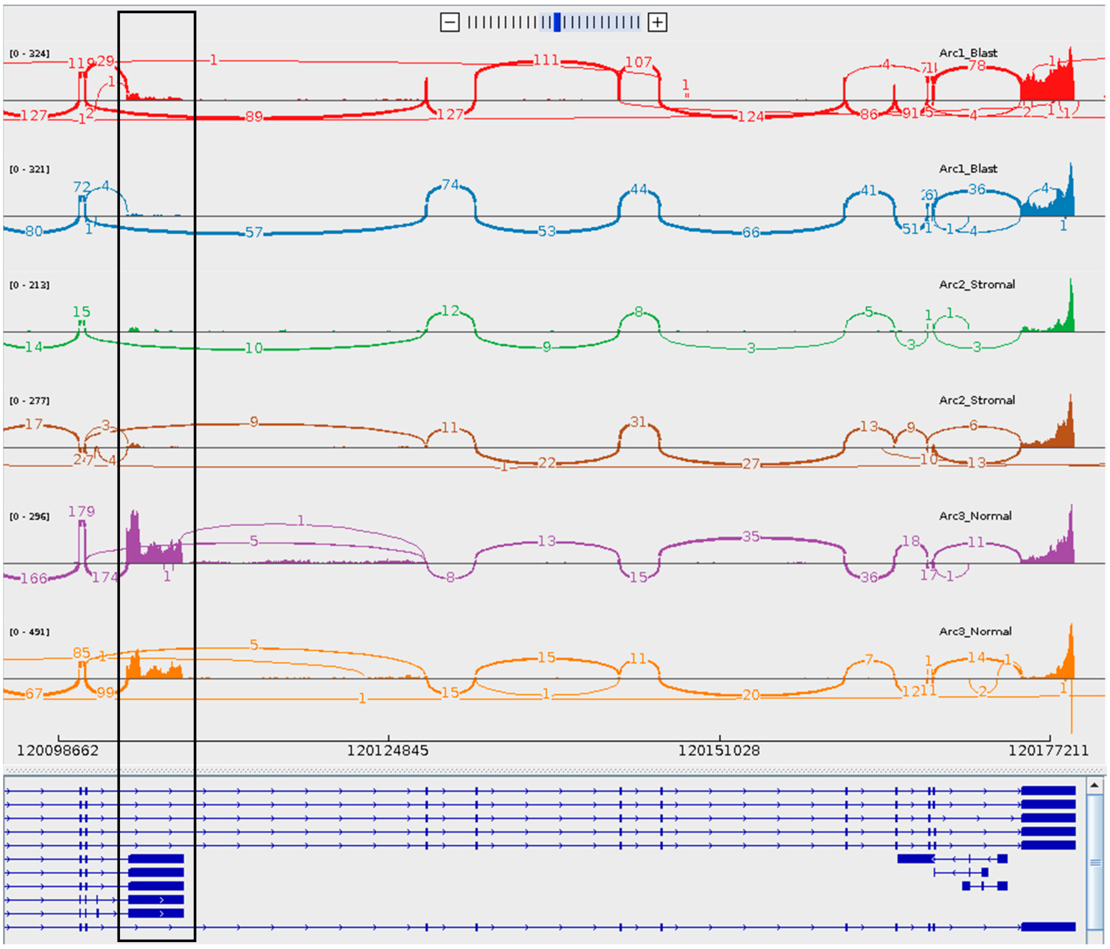

Figure S75: The gene EPB41L5 is alternatively spliced between samples located in different regions of latent space.

Top: A Shashimi plot of representative samples located near different archetypes. It can be seen that normal samples tend to have shorter 3'-ends, which is typical to epithelial cell states [11].

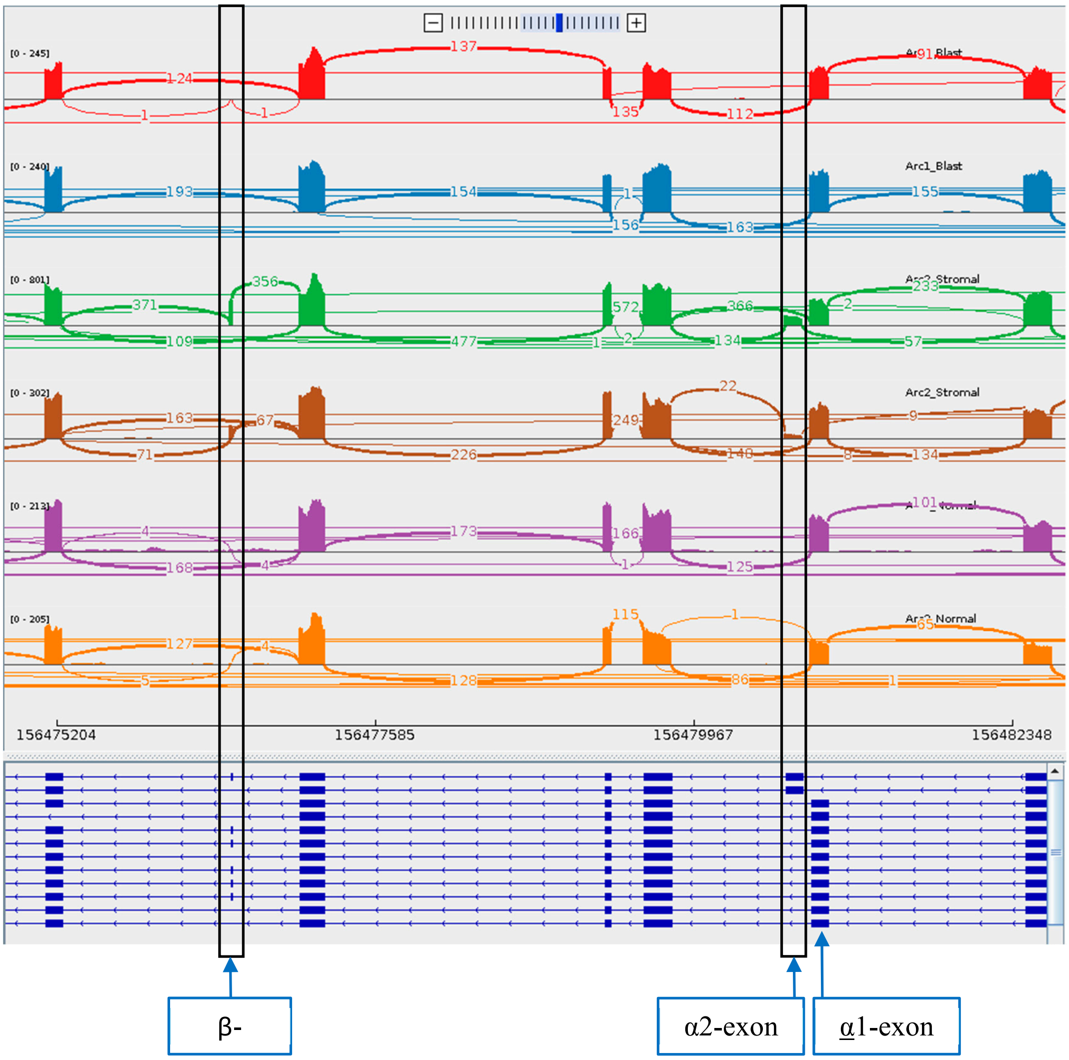

Figure S76: The gene MEF2D is alternatively spliced between samples located in different regions of latent space.

A Shashimi plot of representative samples located near different archetypes. It can be seen that transcripts in tumors located near the stromal archetype tend to have isoforms that are characteristic of muscle cells, that is, including the "beta" and "alpha-2" exons [12]. The inclusion of these exons was previously found to be dependent on the splicing regulators RBFOX1/2 binding downstream, and was also found to be required for myoblast fusion (an essential step of muscle differentiation) in the C2C12 mouse myoblast cell line [3], [13].

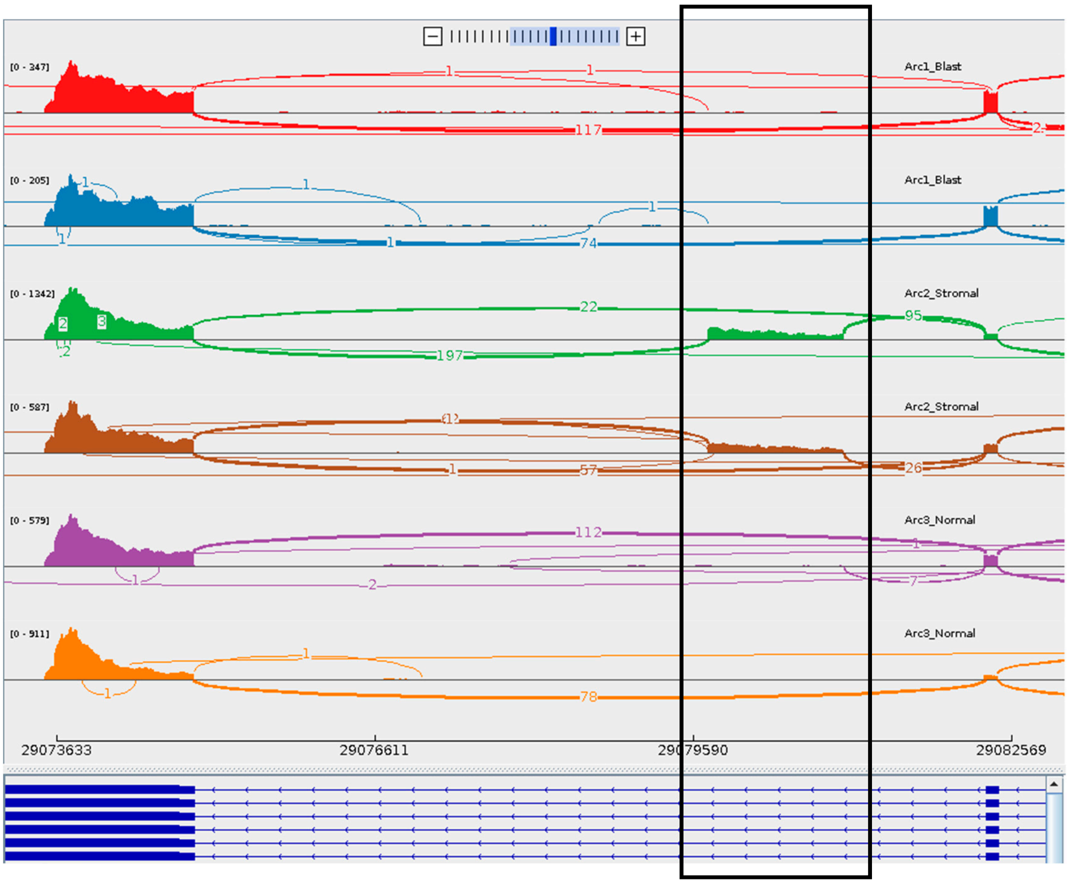

Figure S77: The gene MYO18A is alternatively spliced between samples located in different regions of latent space.

Top: A Shashimi plot of representative samples located near different archetypes. This gene is known to have muscle-specific transcripts that are dependent on the splicing regulator RBFOX2 [3].

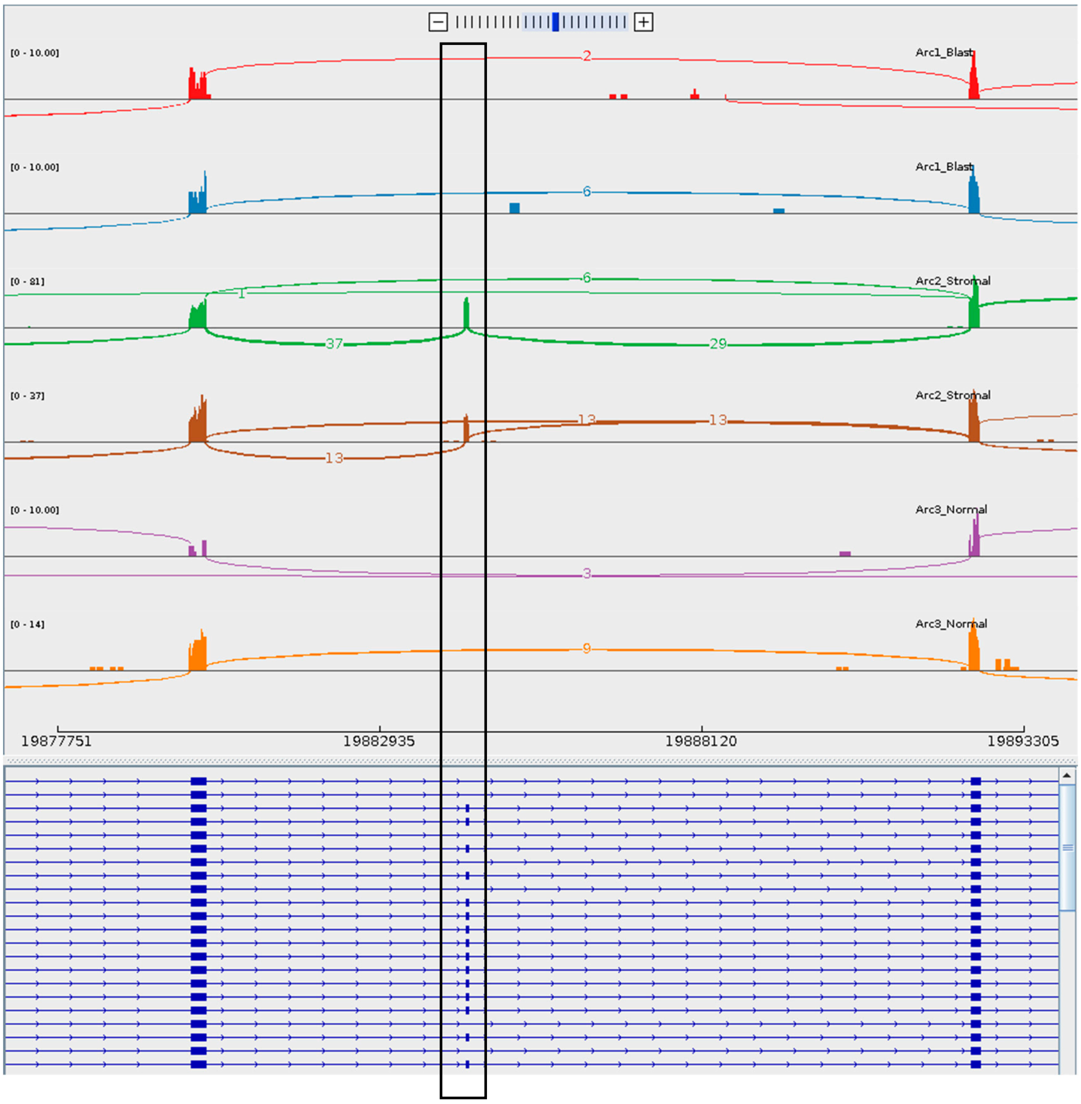

Figure S78: The gene NAV2 is alternatively spliced between samples located in different regions of latent space.

Top: A Shashimi plot of representative samples located near different archetypes. This gene is known to have muscle-specific transcripts that are dependent on the splicing regulator RBFOX2 [3].

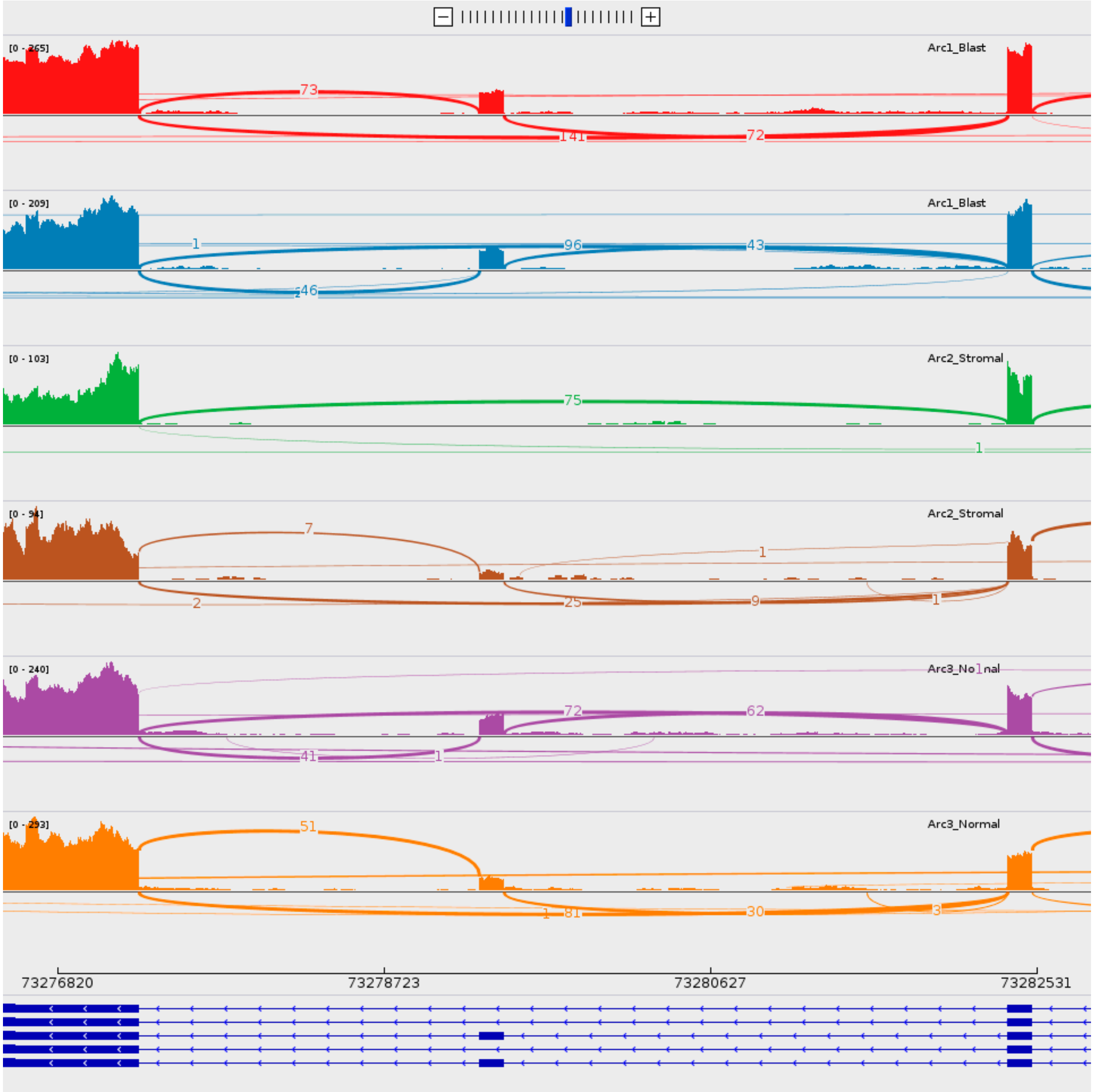

Figure S79: The gene NUMB is alternatively spliced between samples located in different regions of latent space.

Top: A Shashimi plot of representative samples located near different archetypes. Previous observations showed that exon skipping in this gene in human mammary epithelial cells is associated with EMT and occurs when QKI and RBFOX1 are overexpressed [7].

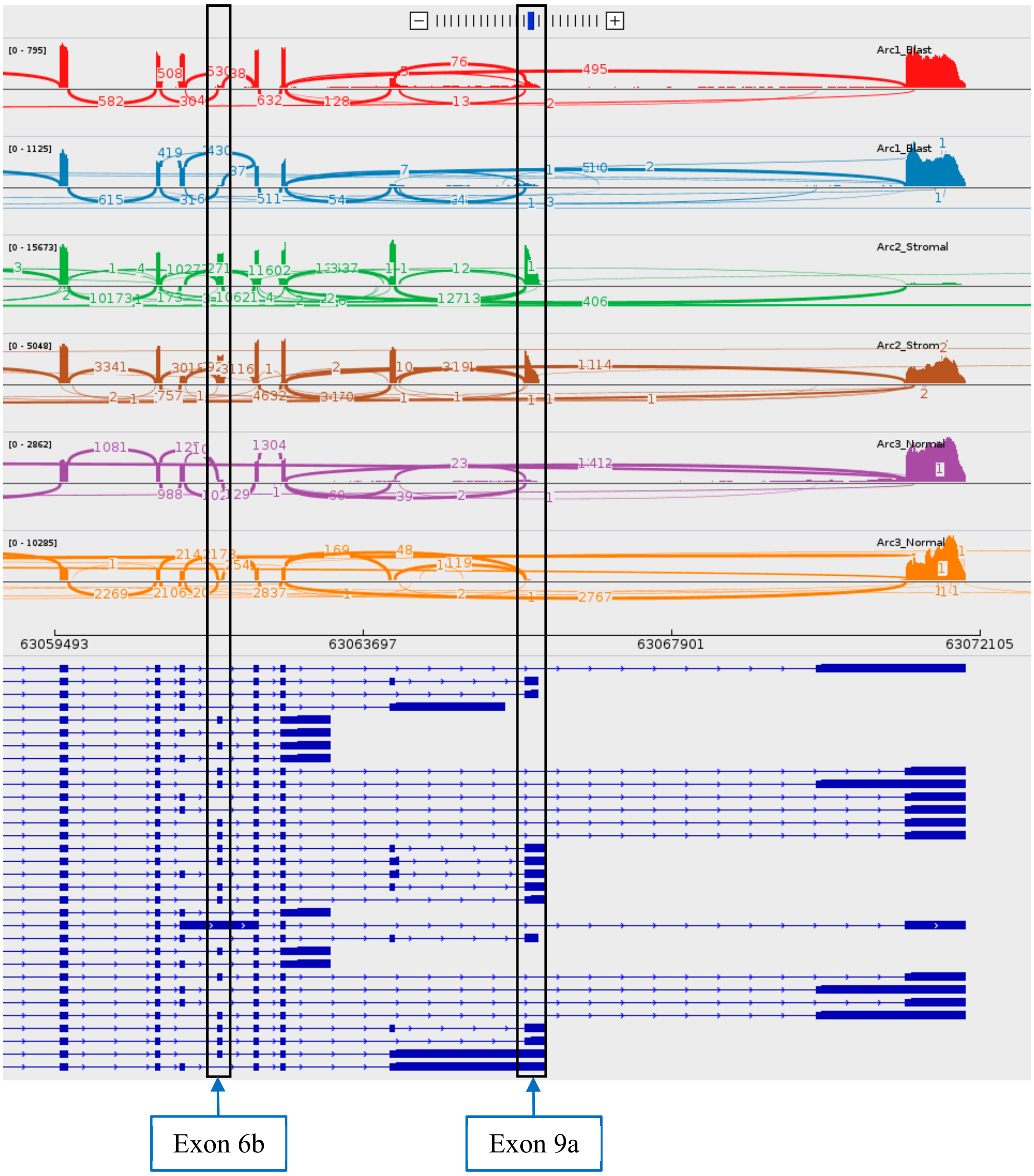

Figure S80: The gene TPM1 is alternatively spliced between samples located in different regions of latent space.

A Shashimi plot of representative samples located near different archetypes. It can be seen that transcripts in tumors located near the stromal archetype tend to have alternative 3'-ends that are specific to skeletal muscle cells (e.g., including exons 6b and 9a) [14]

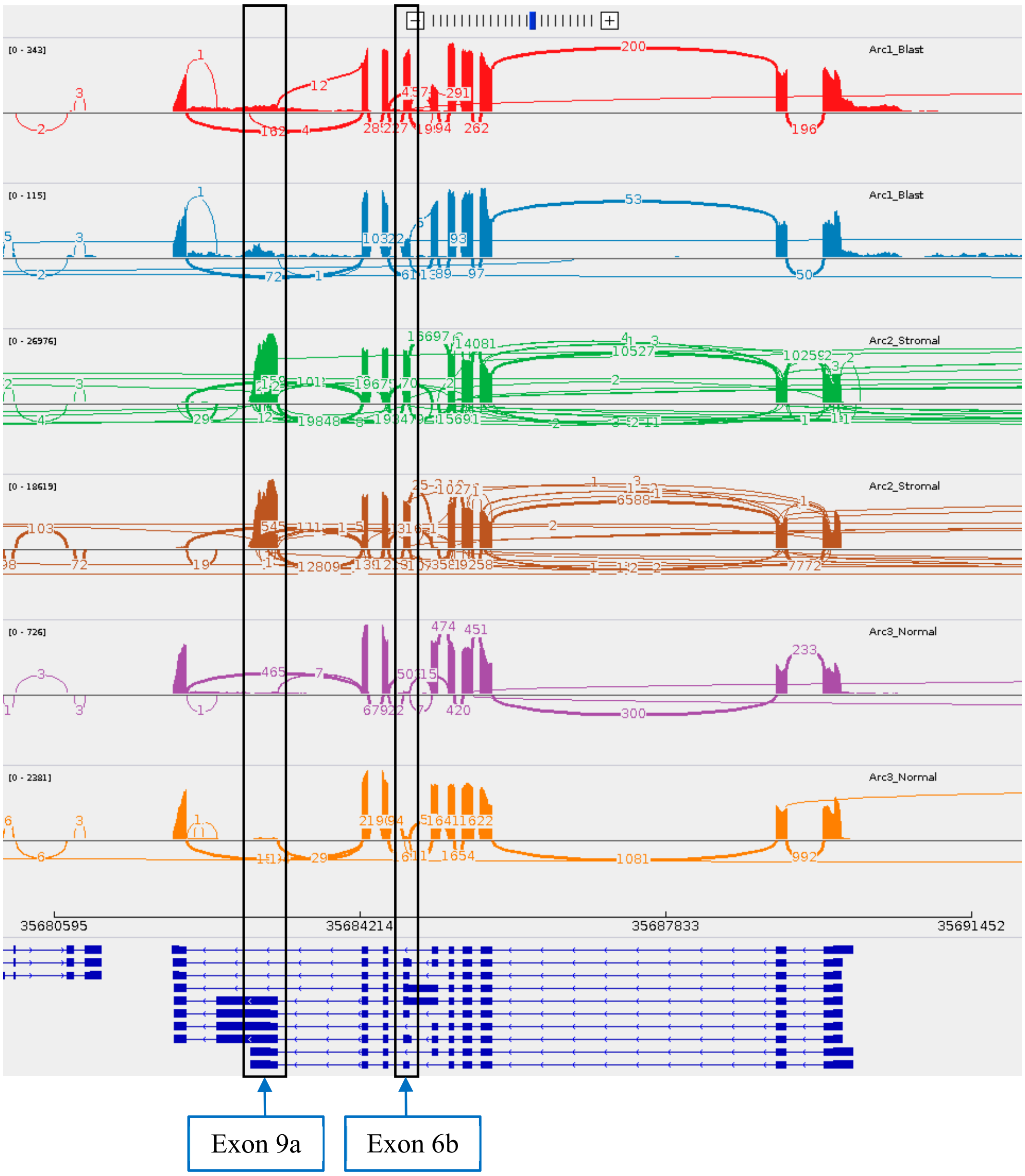

Figure S81: The gene TPM2 is alternatively spliced between samples located in different regions of latent space.

A Shashimi plot of representative samples located near different archetypes. It can be seen that transcripts in tumors located near the stromal archetype tend to have alternative 3'-ends that are specific to skeletal muscle cells, that is, including exons 6b and 9a [14].

Additional putative splicing regulators:

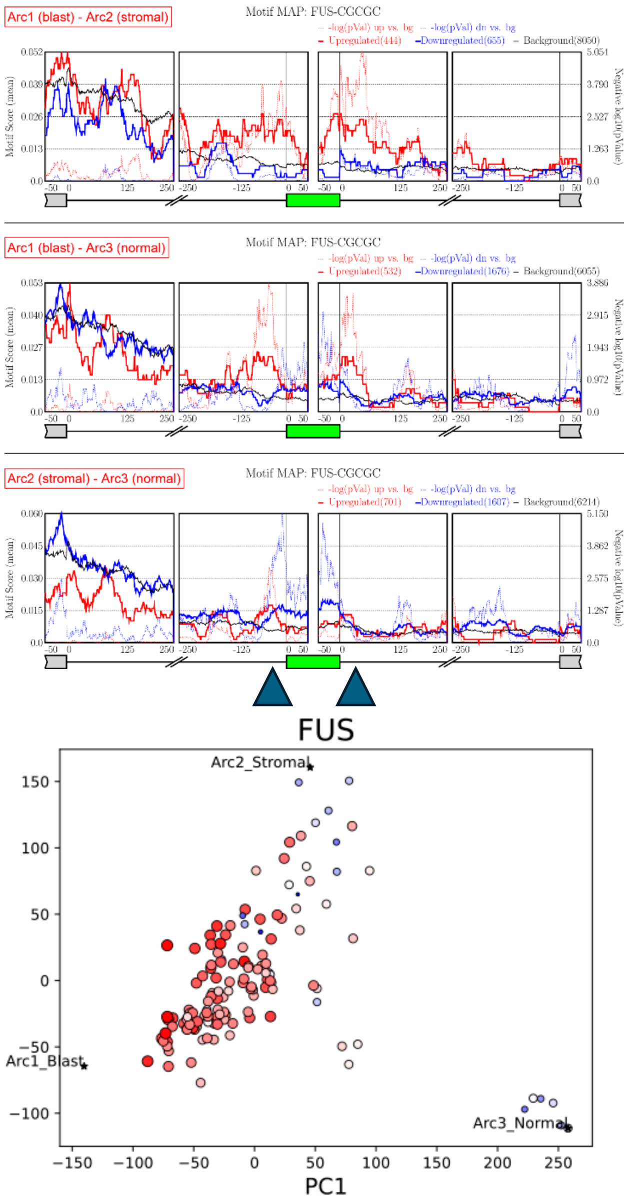

Figure S82: The putative splicing regulator FUS – RNA binding motif enrichment and expression plots.

Top: An RNA binding-motif enrichment map for this putative splicing regulator showing enrichment in the vicinity of exons that are alternatively spliced in different regions of latent space. Bottom: A PCA plot of tumors and normal kidney samples, where each sample is marked according to the expression levels of this putative splicing regulator (large red – high, small blue – low).

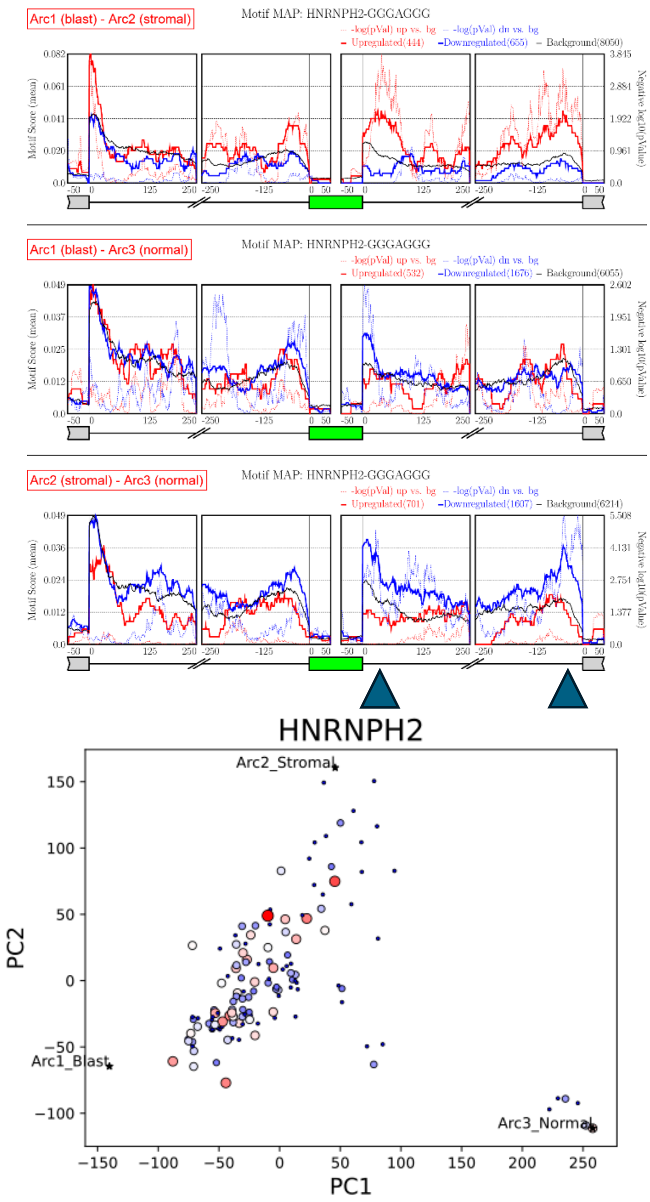

Figure S83: The putative splicing regulator HNRNPH2 – RNA binding motif enrichment and expression plots.

Top: An RNA binding-motif enrichment map for this putative splicing regulator showing enrichment in the vicinity of exons that are alternatively spliced in different regions of latent space. Bottom: A PCA plot of tumors and normal kidney samples, where each sample is marked according to the expression levels of this putative splicing regulator (large red – high, small blue – low).

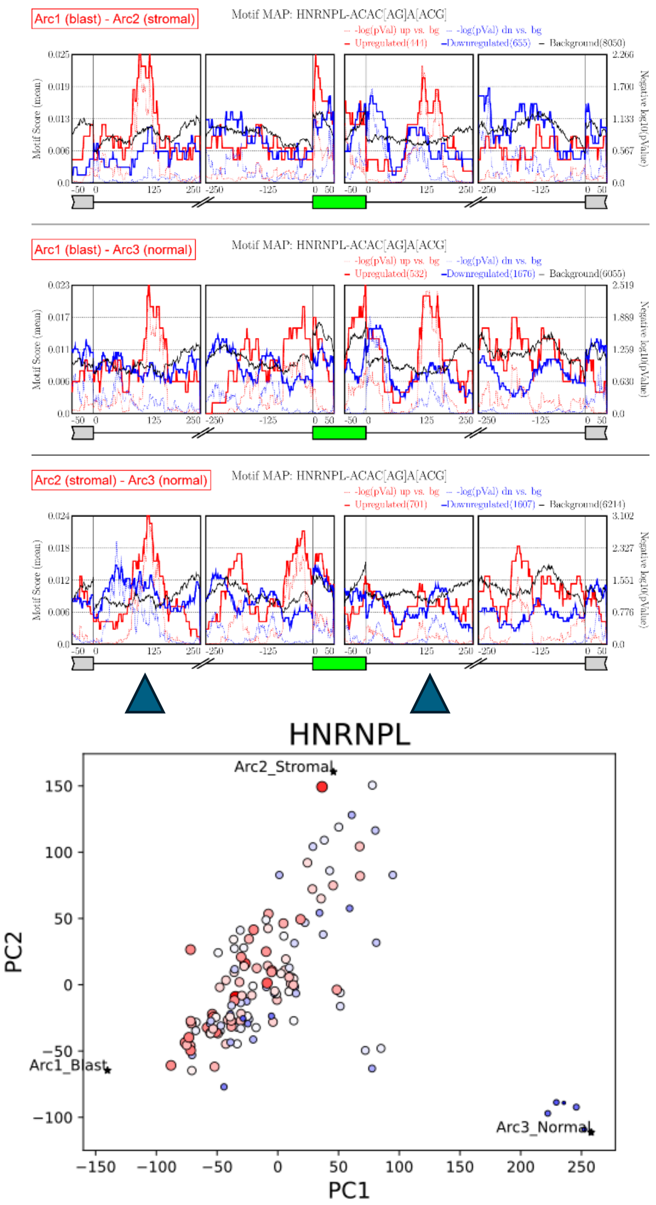

Figure S84: The putative splicing regulator HNRNPL – RNA binding motif enrichment and expression plots.

Top: An RNA binding-motif enrichment map for this putative splicing regulator showing enrichment in the vicinity of exons that are alternatively spliced in different regions of latent space. Bottom: A PCA plot of tumors and normal kidney samples, where each sample is marked according to the expression levels of this putative splicing regulator (large red – high, small blue – low).

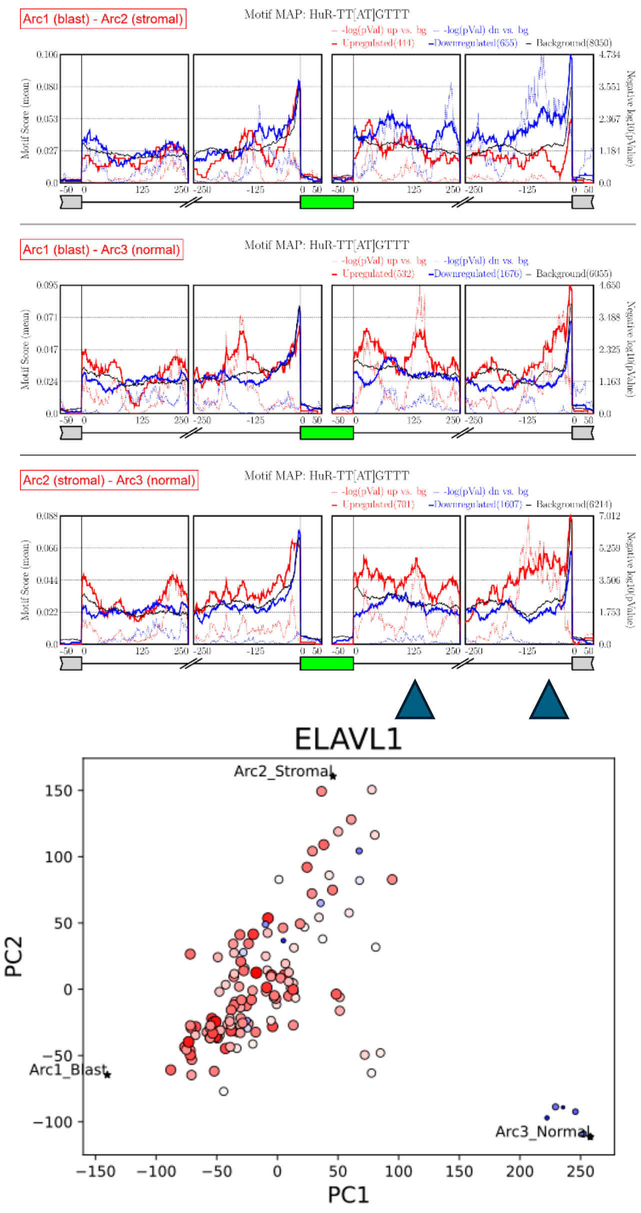

Figure S85: The putative splicing regulator HuR (ELAVL1) – RNA binding motif enrichment and expression plots.

Top: An RNA binding-motif enrichment map for this putative splicing regulator showing enrichment in the vicinity of exons that are alternatively spliced in different regions of latent space. Bottom: A PCA plot of tumors and normal kidney samples, where each sample is marked according to the expression levels of this putative splicing regulator (large red – high, small blue – low).

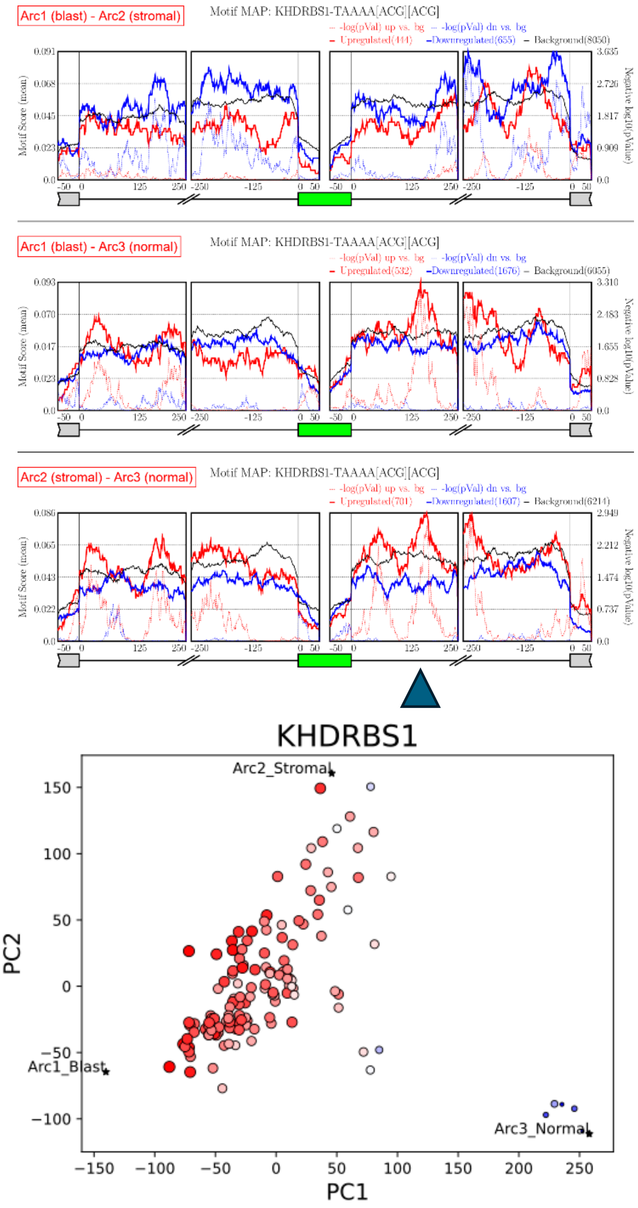

Figure S86: The putative splicing regulator KHDRBS1 – RNA binding motif enrichment and expression plots.

Top: An RNA binding-motif enrichment map for this putative splicing regulator showing enrichment in the vicinity of exons that are alternatively spliced in different regions of latent space. Bottom: A PCA plot of tumors and normal kidney samples, where each sample is marked according to the expression levels of this putative splicing regulator (large red – high, small blue – low).

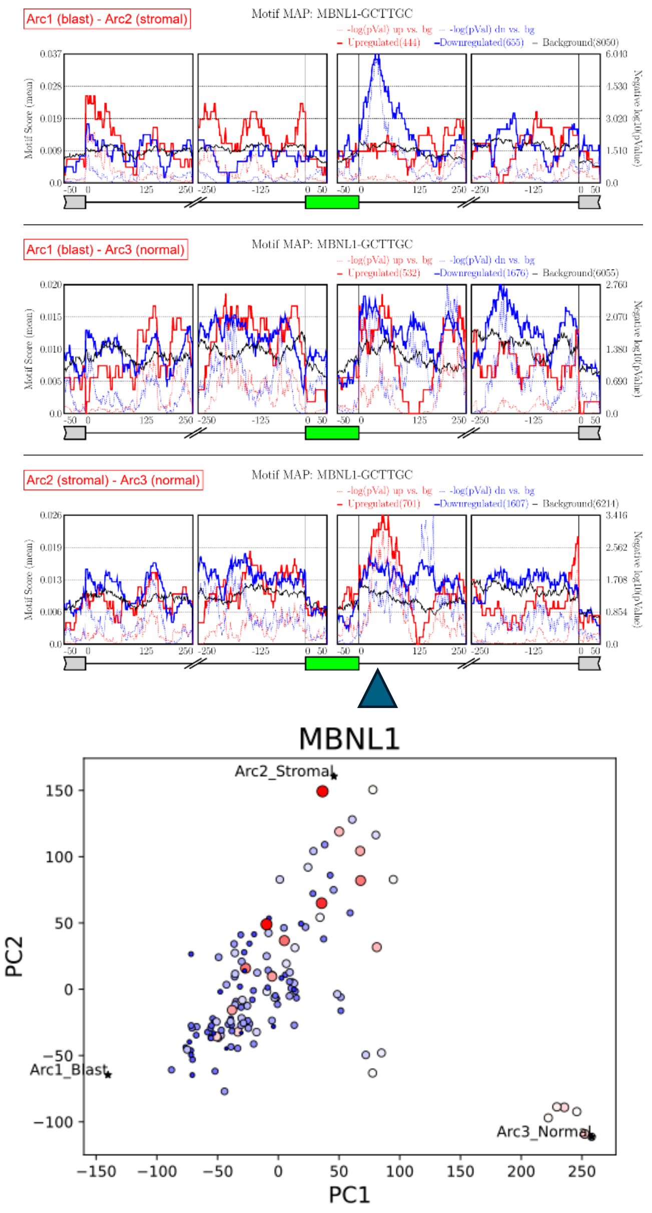

Figure S87: The putative splicing regulator MBNL1 – RNA binding motif enrichment and expression plots.

Top: An RNA binding-motif enrichment map for this putative splicing regulator showing enrichment in the vicinity of exons that are alternatively spliced in different regions of latent space. Bottom: A PCA plot of tumors and normal kidney samples, where each sample is marked according to the expression levels of this putative splicing regulator (large red – high, small blue – low). MBNL1 is known to be associated with muscle differentiation [15].

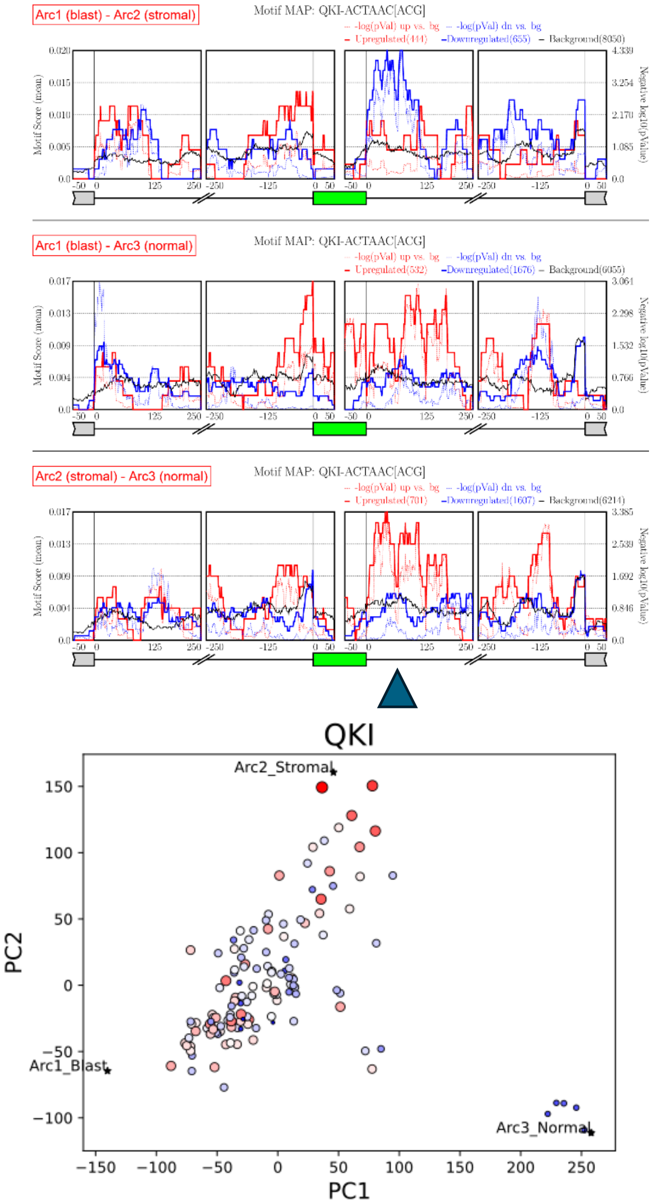

Figure S88: The putative splicing regulator QKI – RNA binding motif enrichment and expression plots.

Top: An RNA binding-motif enrichment map for this putative splicing regulator showing enrichment in the vicinity of exons that are alternatively spliced in different regions of latent space. Bottom: A PCA plot of tumors and normal kidney samples, where each sample is marked according to the expression levels of this putative splicing regulator (large red – high, small blue – low).

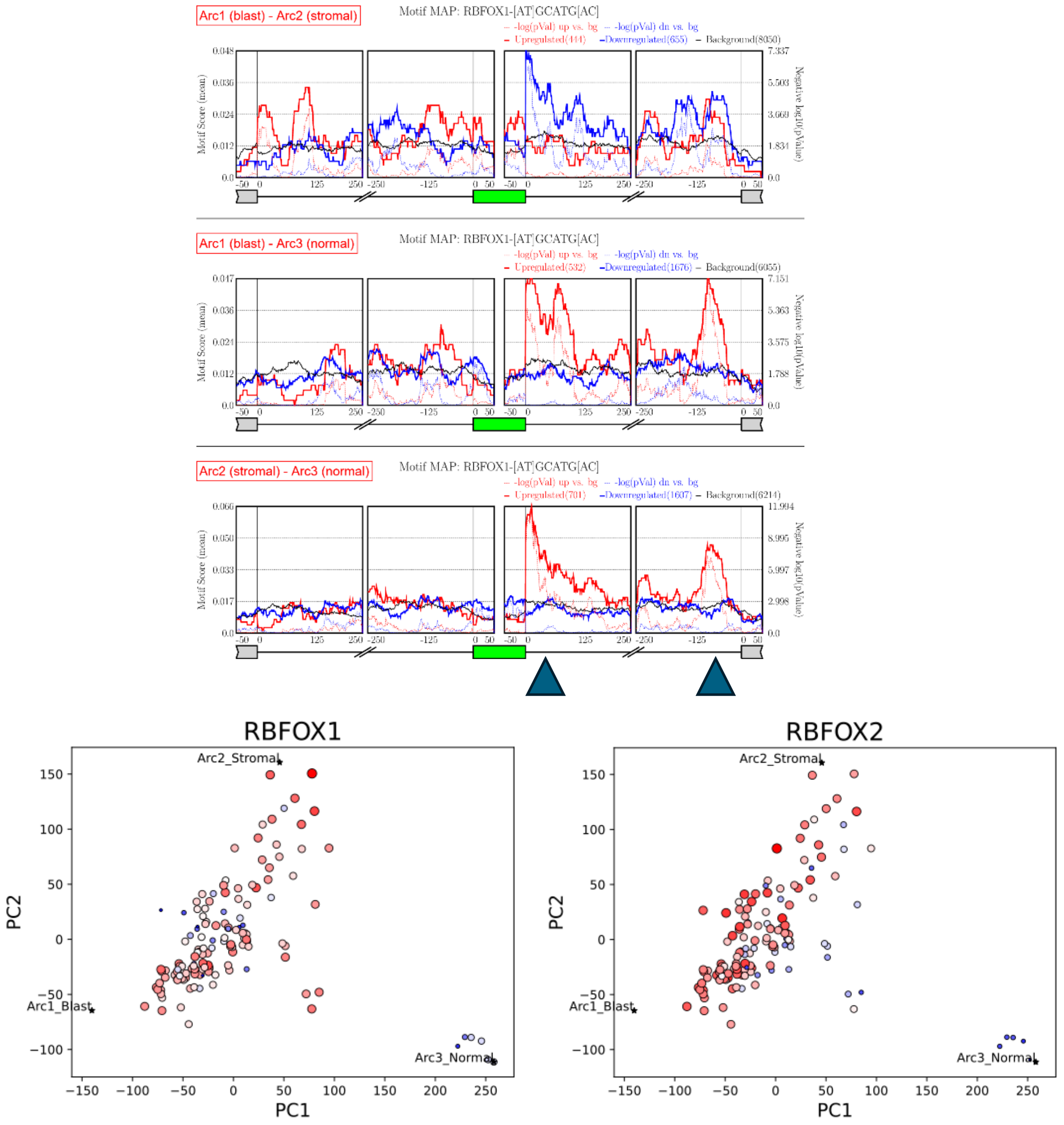

Figure S89: The putative splicing regulators RBFOX1 and RBFOX2 – RNA binding motif enrichment and expression plots.

Top: An RNA binding-motif enrichment map for this putative splicing regulator showing enrichment in the vicinity of exons that are alternatively spliced in different regions of latent space. Bottom: A PCA plot of tumors and normal kidney samples, where each sample is marked according to the expression levels of this putative splicing regulator (large red – high, small blue – low). Note that according to [16] and the CISBP-RNA database [17] (http://cisbp-rna.ccbr.utoronto.ca) the proteins RBFOX1 and RBFOX2 both bind to the same mRNA motif ([AT]GCATG[AC]).

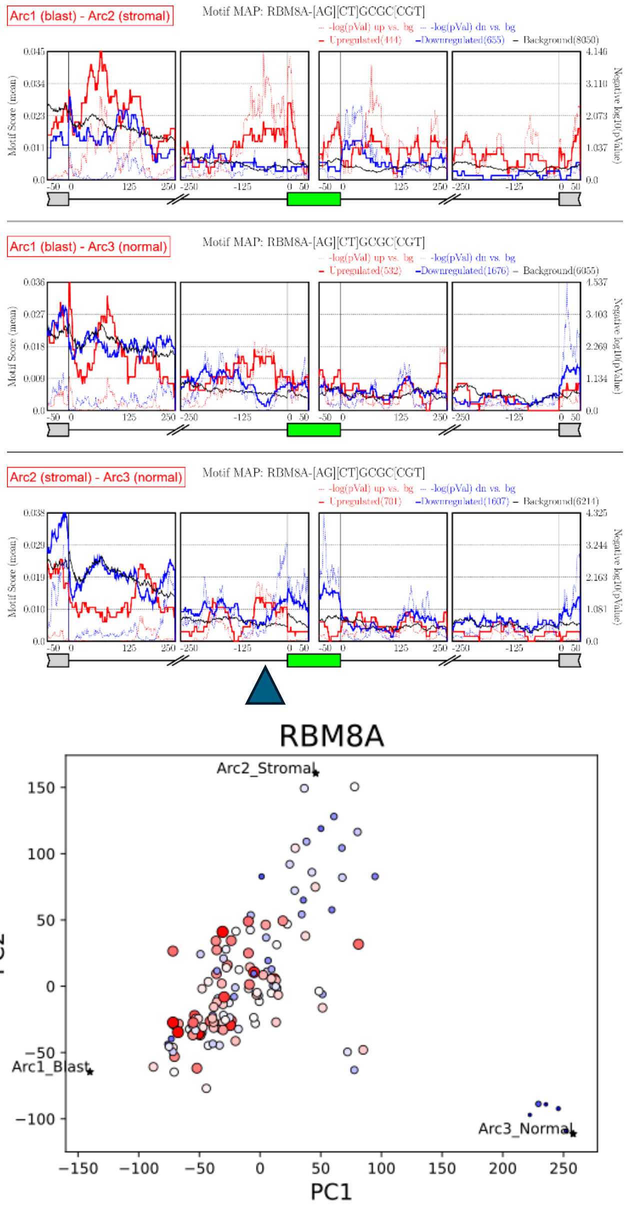

Figure S90: The putative splicing regulator RBM8A – RNA binding motif enrichment and expression plots.

Top: An RNA binding-motif enrichment map for this putative splicing regulator showing enrichment in the vicinity of exons that are alternatively spliced in different regions of latent space. Bottom: A PCA plot of tumors and normal kidney samples, where each sample is marked according to the expression levels of this putative splicing regulator (large red – high, small blue – low).

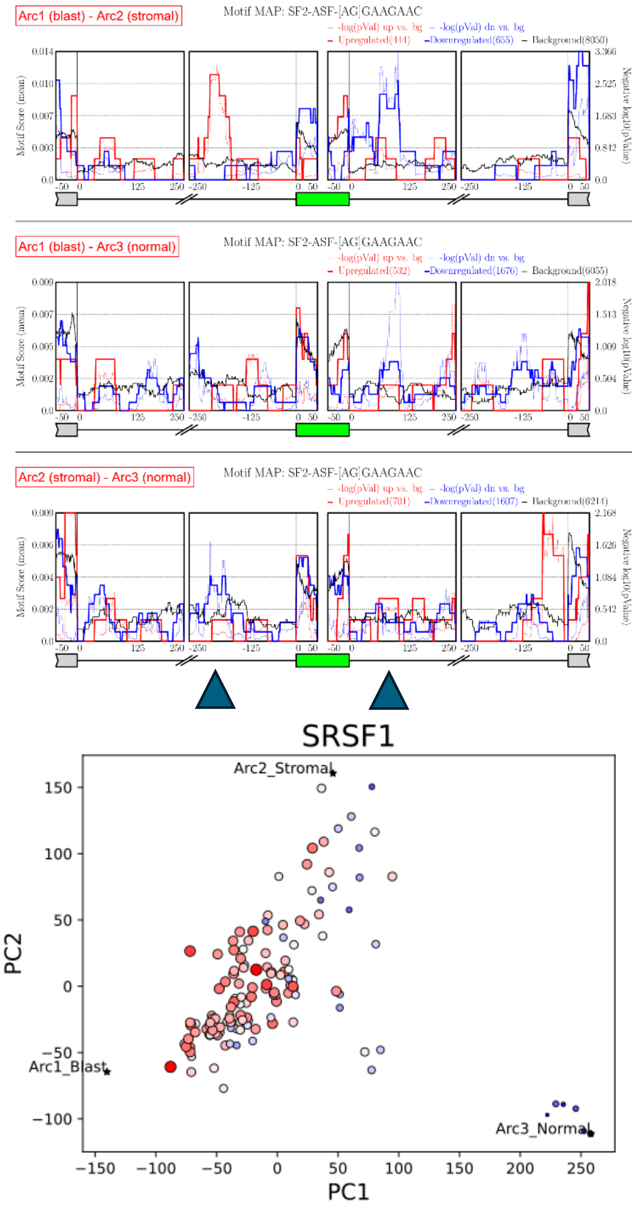

Figure S91: The putative splicing regulator SF2-ASF (SRSF1) – RNA binding motif enrichment and expression plots.

Top: An RNA binding-motif enrichment map for this putative splicing regulator showing enrichment in the vicinity of exons that are alternatively spliced in different regions of latent space. Bottom: A PCA plot of tumors and normal kidney samples, where each sample is marked according to the expression levels of this putative splicing regulator (large red – high, small blue – low).

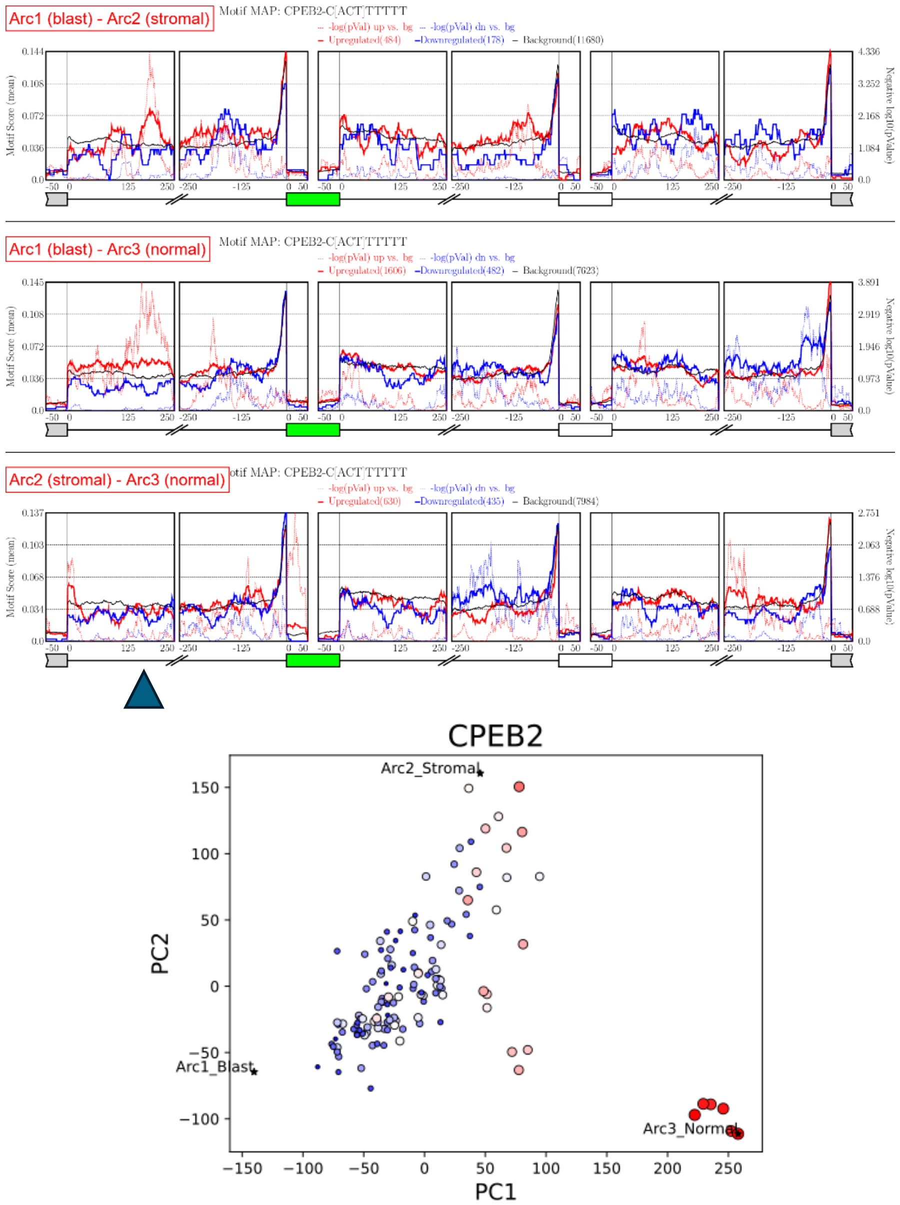

Figure S92: The putative splicing regulator CPEB2 – RNA binding motif enrichment and expression plots.

Top: An RNA binding-motif enrichment map for this putative splicing regulator showing enrichment in the vicinity of exons that are alternatively spliced in different regions of latent space. Bottom: A PCA plot of tumors and normal kidney samples, where each sample is marked according to the expression levels of this putative splicing regulator (large red – high, small blue – low).

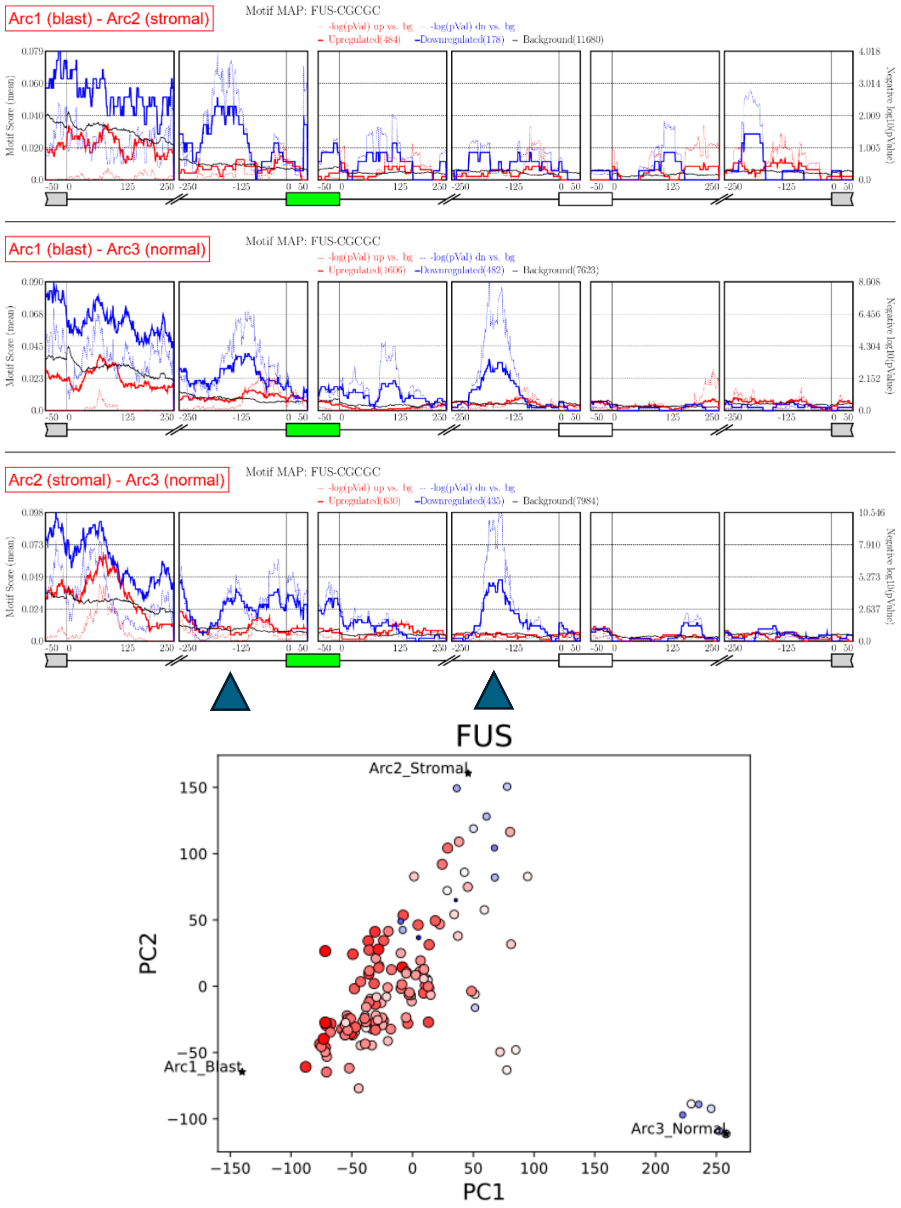

Figure S93: The putative splicing regulator FUS – RNA binding motif enrichment and expression plots.

Top: An RNA binding-motif enrichment map for this putative splicing regulator showing enrichment in the vicinity of exons that are alternatively spliced in different regions of latent space. Bottom: A PCA plot of tumors and normal kidney samples, where each sample is marked according to the expression levels of this putative splicing regulator (large red – high, small blue – low).

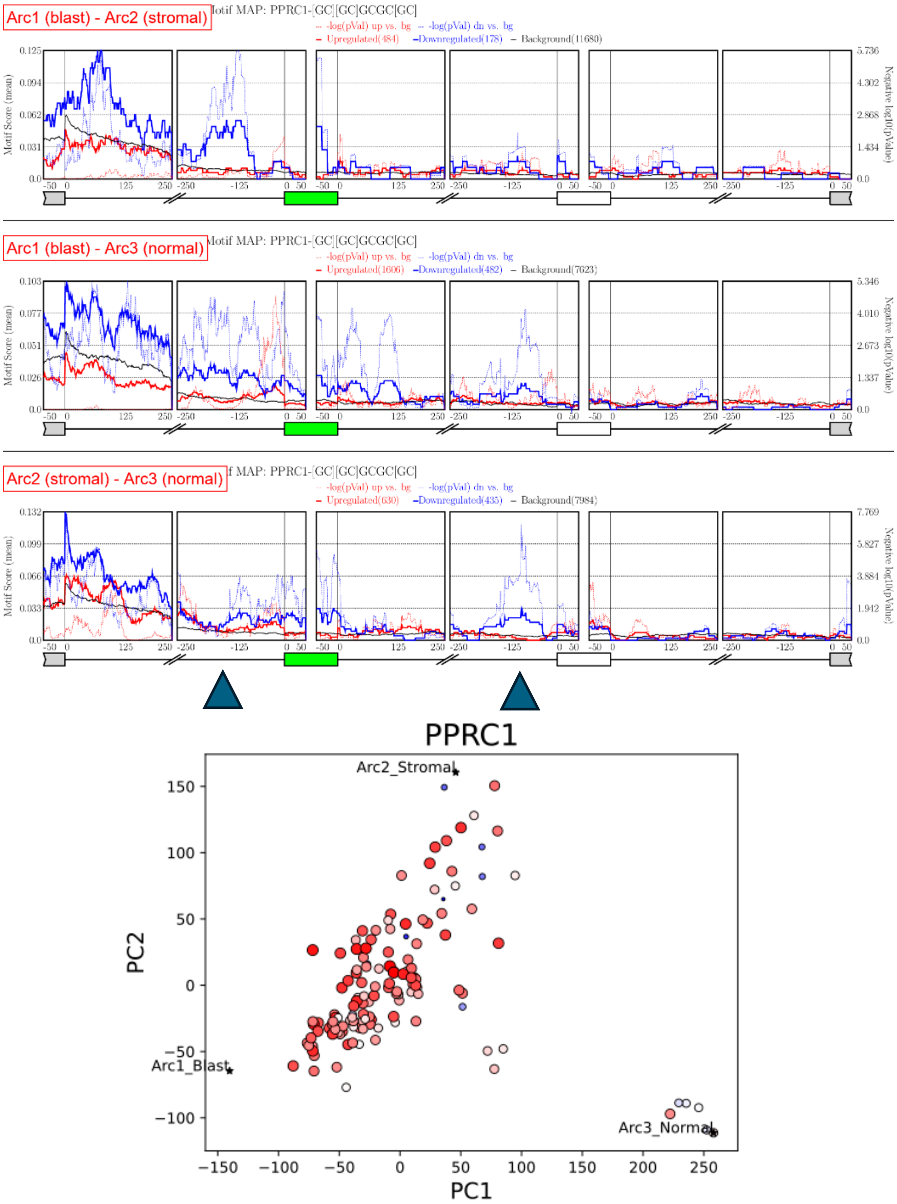

Figure S94: The putative splicing regulator PPRC1 – RNA binding motif enrichment and expression plots.

Top: An RNA binding-motif enrichment map for this putative splicing regulator showing enrichment in the vicinity of exons that are alternatively spliced in different regions of latent space. Bottom: A PCA plot of tumors and normal kidney samples, where each sample is marked according to the expression levels of this putative splicing regulator (large red – high, small blue – low).

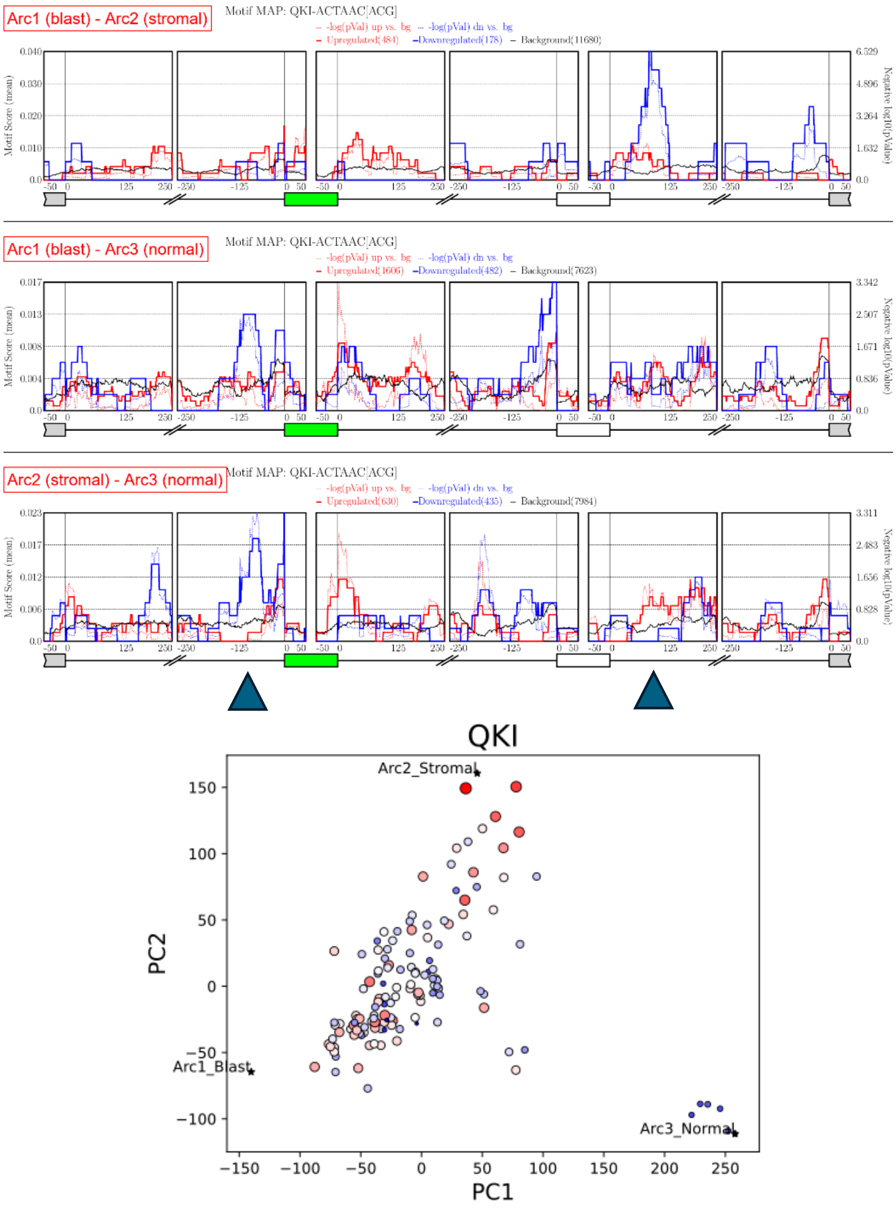

Figure S95: The putative splicing regulator QKI – RNA binding motif enrichment and expression plots.

Top: An RNA binding-motif enrichment map for this putative splicing regulator showing enrichment in the vicinity of exons that are alternatively spliced in different regions of latent space. Bottom: A PCA plot of tumors and normal kidney samples, where each sample is marked according to the expression levels of this putative splicing regulator (large red – high, small blue – low).

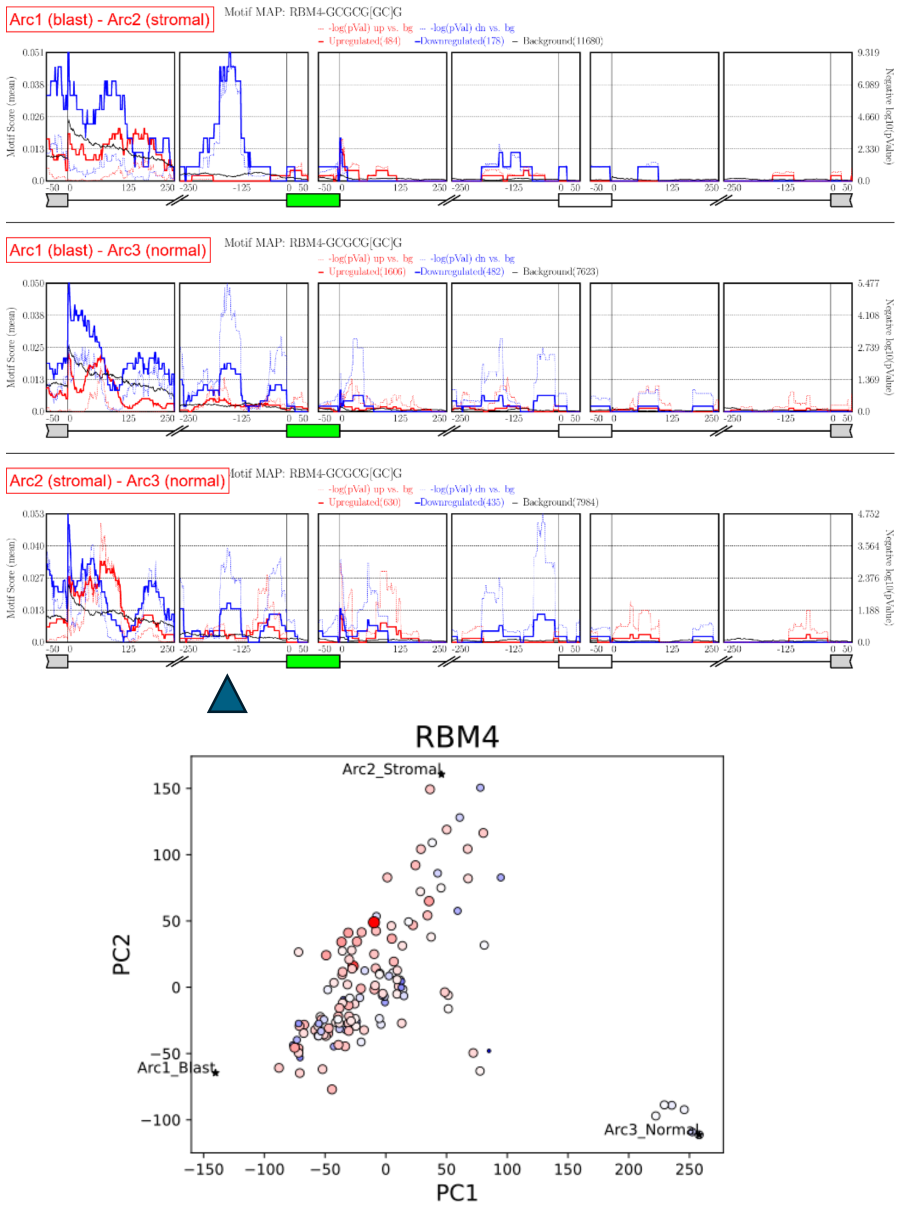

Figure S96: The putative splicing regulator RBM4 – RNA binding motif enrichment and expression plots.

Top: An RNA binding-motif enrichment map for this putative splicing regulator showing enrichment in the vicinity of exons that are alternatively spliced in different regions of latent space. Bottom: A PCA plot of tumors and normal kidney samples, where each sample is marked according to the expression levels of this putative splicing regulator (large red – high, small blue – low).

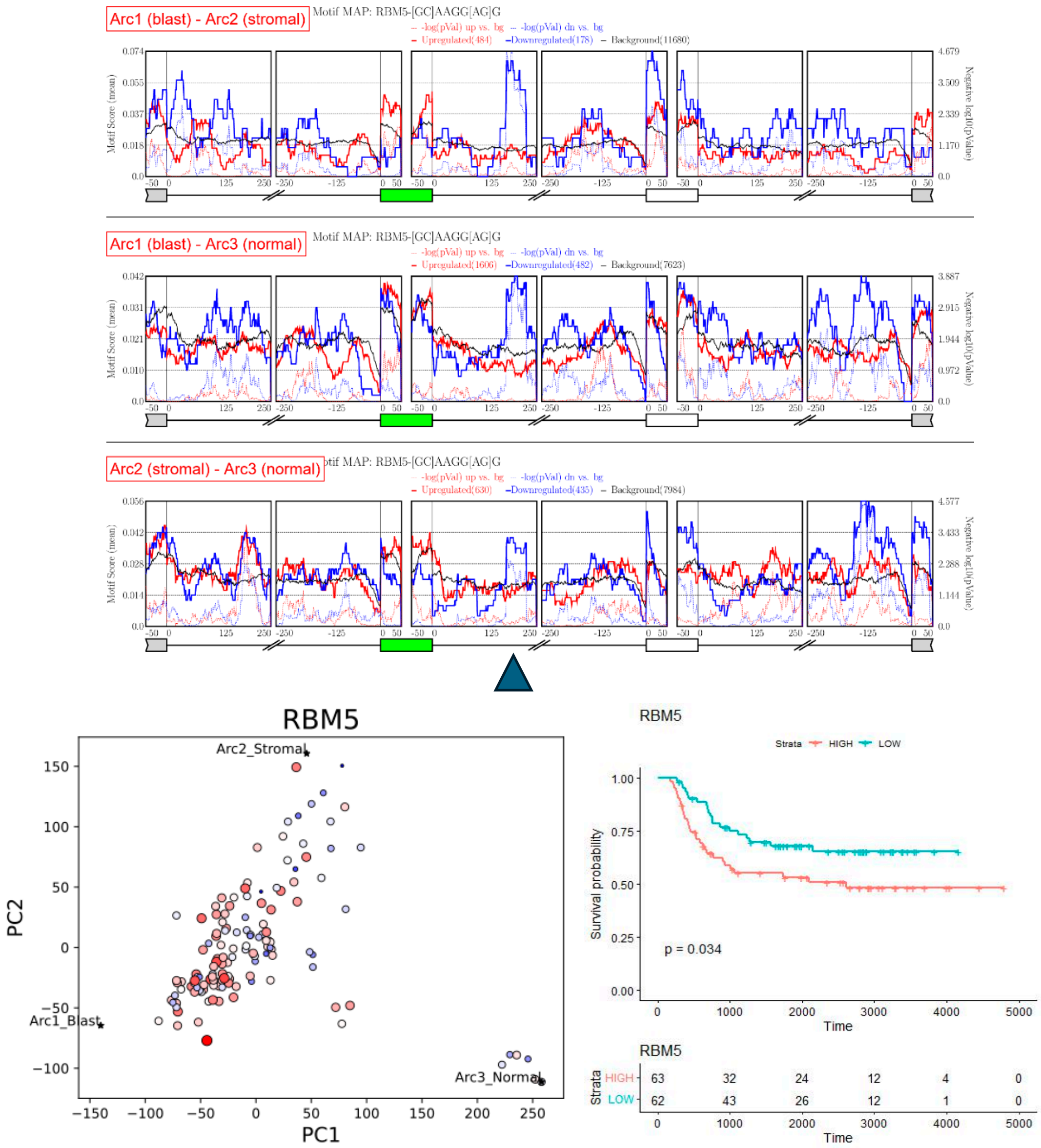

Figure S97: The putative splicing regulator RBM5 – RNA binding motif enrichment, expression, and survival plots.

Top: An RNA binding-motif enrichment map for this putative splicing regulator showing enrichment in the vicinity of exons that are alternatively spliced in different regions of latent space. Bottom left: A PCA plot of tumors and normal kidney samples, where each sample is marked according to the expression levels of this putative splicing regulator (large red – high, small blue – low). Bottom right: Kaplan-Meier survival curves of TARGET Wilms' tumor patients grouped by expression levels of RBM5.

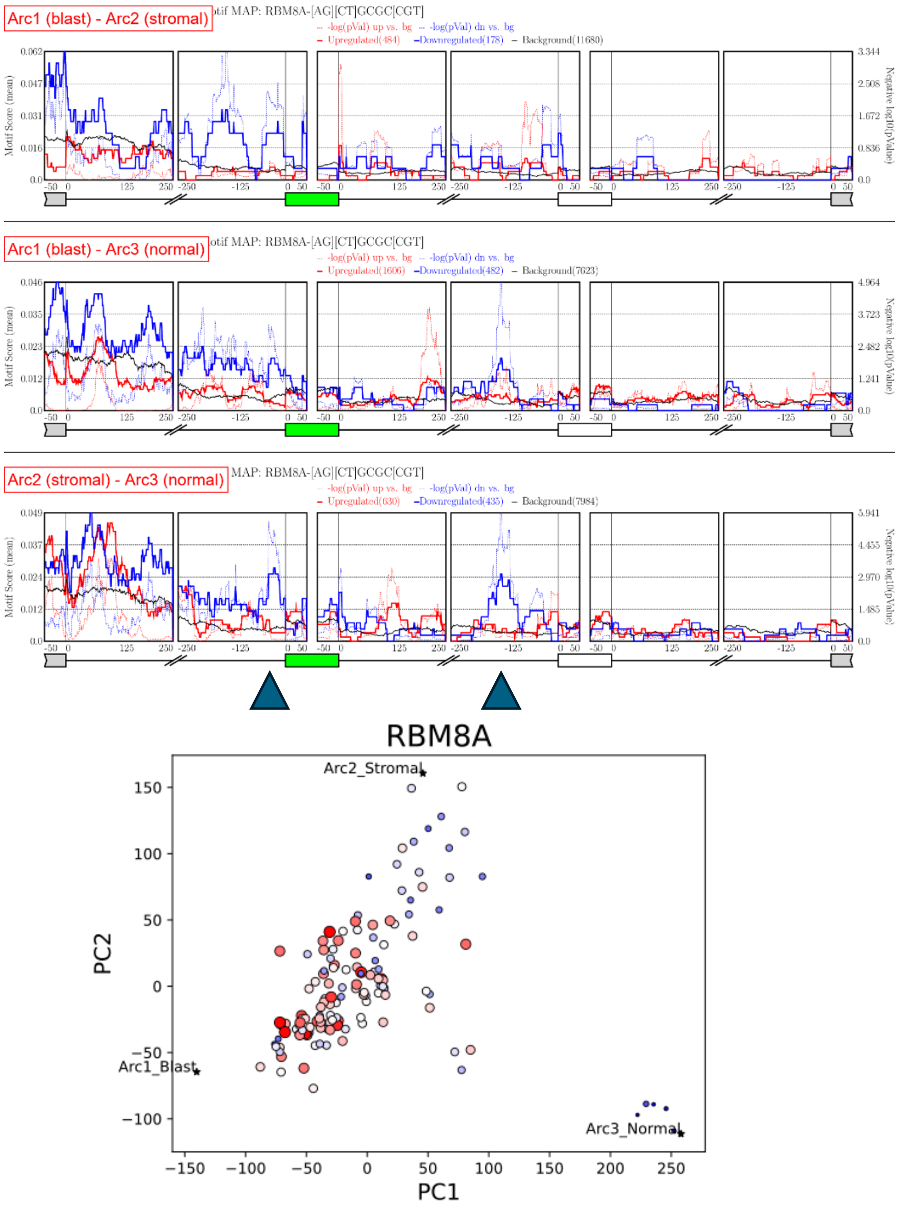

Figure S98: The putative splicing regulator RBM8A – RNA binding motif enrichment and expression plots.

Top: An RNA binding-motif enrichment map for this putative splicing regulator showing enrichment in the vicinity of exons that are alternatively spliced in different regions of latent space. Bottom: A PCA plot of tumors and normal kidney samples, where each sample is marked according to the expression levels of this putative splicing regulator (large red – high, small blue – low).

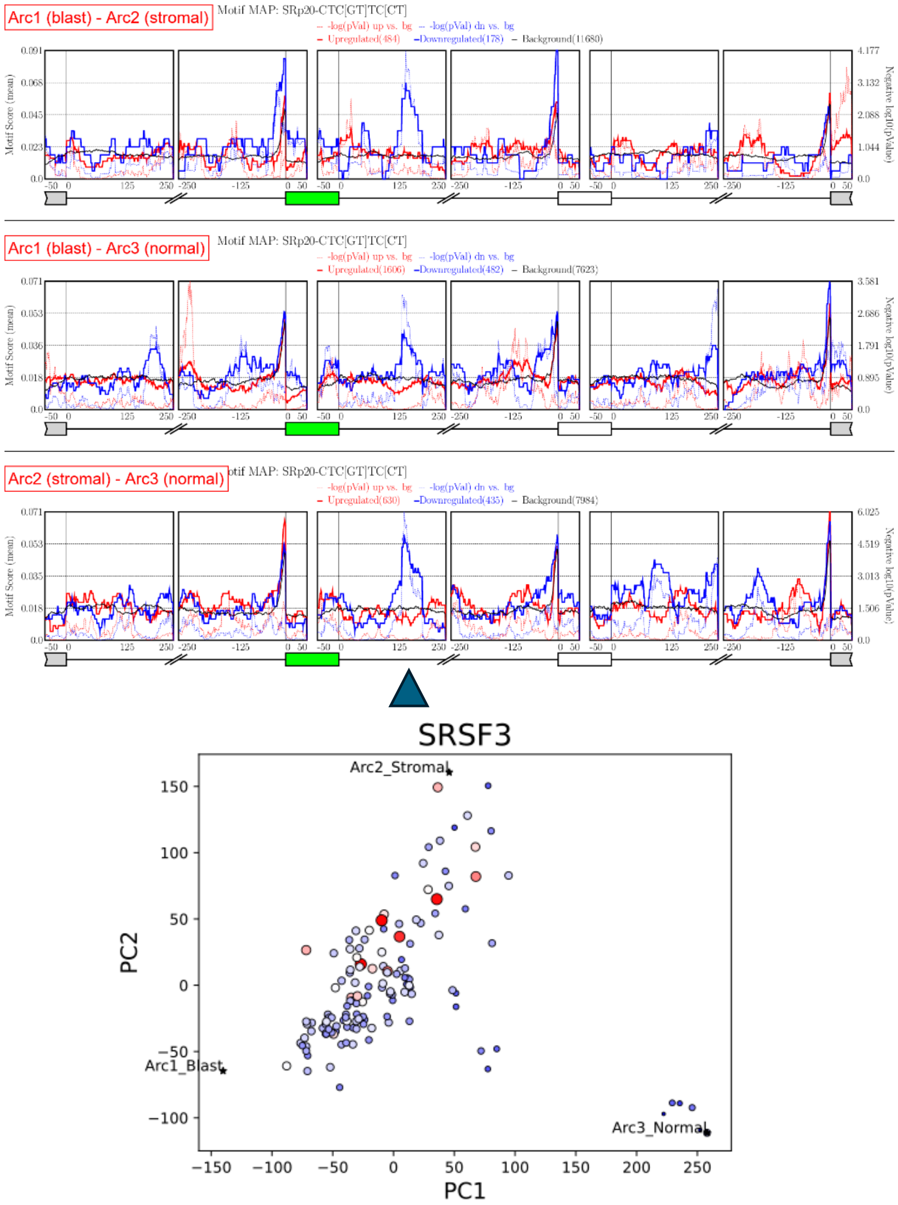

Figure S99: The putative splicing regulator SRp20 (SRSF3) – RNA binding motif enrichment and expression plots.

Top: An RNA binding-motif enrichment map for this putative splicing regulator showing enrichment in the vicinity of exons that are alternatively spliced in different regions of latent space. Bottom: A PCA plot of tumors and normal kidney samples, where each sample is marked according to the expression levels of this putative splicing regulator (large red – high, small blue – low).

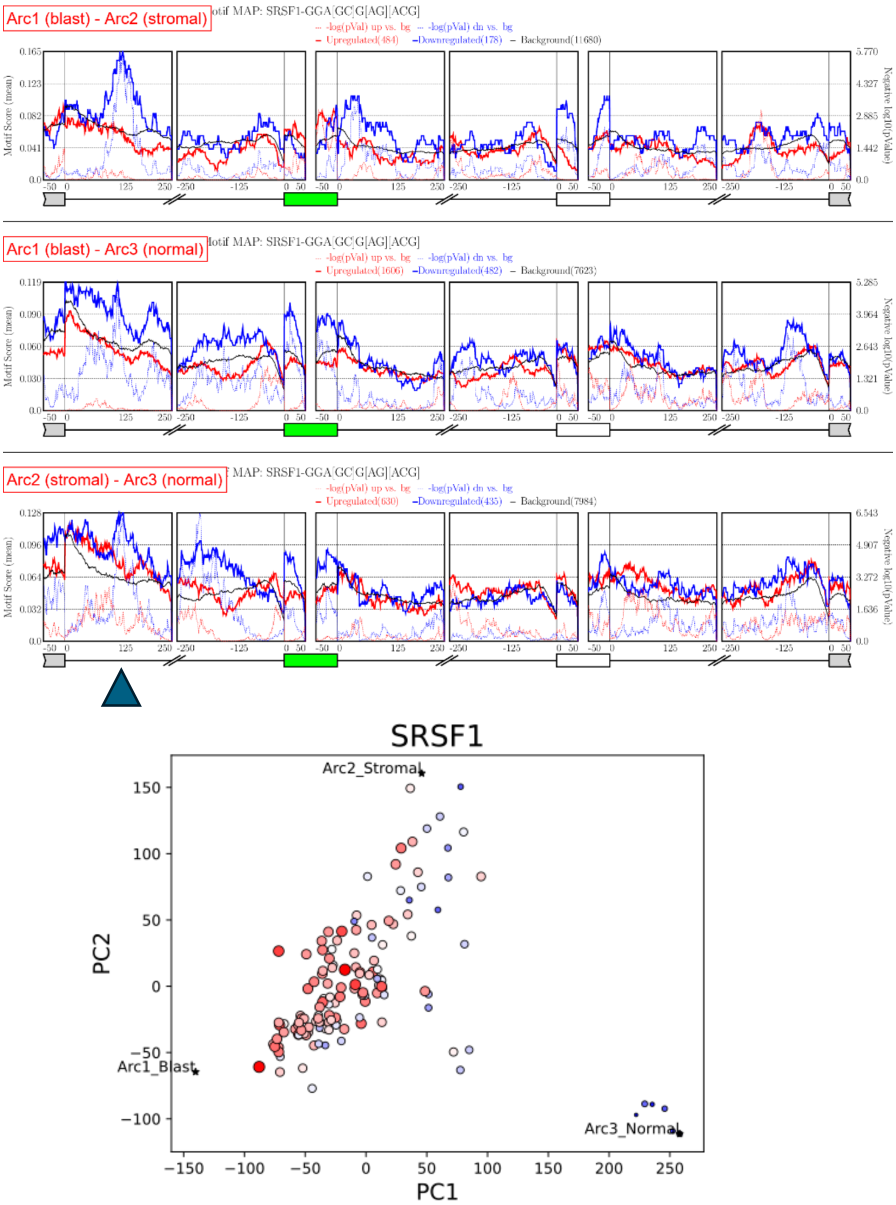

Figure S100: The putative splicing regulator SRSF1 – RNA binding motif enrichment and expression plots.

Top: An RNA binding-motif enrichment map for this putative splicing regulator showing enrichment in the vicinity of exons that are alternatively spliced in different regions of latent space. Bottom: A PCA plot of tumors and normal kidney samples, where each sample is marked according to the expression levels of this putative splicing regulator (large red – high, small blue – low).

REFERENCES

[1] J. Wegert *et al.*, "Mutations in the SIX1/2 Pathway and the DROSHA/DGCR8 miRNA Microprocessor Complex Underlie High-Risk Blastemal Type Wilms Tumors," *Cancer Cell*, vol. 27, no. 2, pp. 298–311, Feb. 2015, doi: 10.1016/j.ccell.2015.01.002.

[2] S. Gadd *et al.*, "A Children's Oncology Group and TARGET initiative exploring the genetic landscape of Wilms tumor," *Nat Genet*, vol. 49, no. 10, Art. no. 10, Oct. 2017, doi: 10.1038/ng.3940.

[3] R. K. Singh *et al.*, "Rbfox2-Coordinated Alternative Splicing of Mef2d and Rock2 Controls Myoblast Fusion during Myogenesis," *Molecular Cell*, vol. 55, no. 4, pp. 592–603, Aug. 2014, doi: 10.1016/j.molcel.2014.06.035.

[4] E. Sebestyén *et al.*, "Large-scale analysis of genome and transcriptome alterations in multiple tumors unveils novel cancer-relevant splicing networks," *Genome Res.*, vol. 26, no. 6, pp. 732–744, Jun. 2016, doi: 10.1101/gr.199935.115.

[5] J. Qin and J. Xu, "Arginine methylation in the epithelial-to-mesenchymal transition," *The FEBS Journal*, vol. 289, no. 23, pp. 7292–7303, 2022, doi: 10.1111/febs.16152.

[6] J. Zhong *et al.*, "Identification and characterization of novel spliced variants of PRMT2 in breast carcinoma," *The FEBS Journal*, vol. 279, no. 2, pp. 316–335, 2012, doi: 10.1111/j.1742-4658.2011.08426.x.

[7] J. Li *et al.*, "An alternative splicing switch in FLNB promotes the mesenchymal cell state in human breast cancer," *eLife*, vol. 7, p. e37184, Jul. 2018, doi: 10.7554/eLife.37184.

[8] V. Gonçalves, P. Matos, and P. Jordan, "Antagonistic SR proteins regulate alternative splicing of tumor-related Rac1b downstream of the PI3-kinase and Wnt pathways," *Human Molecular Genetics*, vol. 18, no. 19, pp. 3696–3707, Oct. 2009, doi: 10.1093/hmg/ddp317.

[9] T. P. Keeton, S. E. Burk, and G. E. Shull, "Alternative splicing of exons encoding the calmodulin-binding domains and C termini of plasma membrane Ca(2+)-ATPase isoforms 1, 2, 3, and 4.," *Journal of Biological Chemistry*, vol. 268, no. 4, pp. 2740–2748, Feb. 1993, doi: 10.1016/S0021-9258(18)53836-9.

[10] T. P. Stauffer, H. Hilfiker, E. Carafoli, and E. E. Strehler, "Quantitative analysis of alternative splicing options of human plasma membrane calcium pump genes.," *Journal of Biological Chemistry*, vol. 268, no. 34, pp. 25993–26003, Dec. 1993, doi: 10.1016/S0021-9258(19)74484-6.

[11] C. C. Warzecha and R. P. Carstens, "Complex changes in alternative pre-mRNA splicing play a central role in the epithelial-to-mesenchymal transition (EMT)," *Seminars in Cancer Biology*, vol. 22, no. 5, pp. 417–427, Oct. 2012, doi: 10.1016/j.semcancer.2012.04.003.

[12] B. Zhu, B. Ramachandran, and T. Gulick, "Alternative Pre-mRNA Splicing Governs Expression of a Conserved Acidic Transactivation Domain in Myocyte Enhancer Factor 2 Factors of Striated Muscle and Brain *," *Journal of Biological Chemistry*, vol. 280, no. 31, pp. 28749–28760, Aug. 2005, doi: 10.1074/jbc.M502491200.

[13] V. Runfola, S. Sebastian, F. J. Dilworth, and D. Gabellini, "Rbfox proteins regulate tissue-specific alternative splicing of Mef2D required for muscle differentiation," *J Cell Sci*, vol. 128, no. 4, pp. 631–637, Feb. 2015, doi: 10.1242/jcs.161059.

[14] M. Gimona, A. Watakabe, and D. M. Helfman, "Specificity of dimer formation in tropomyosins: influence of alternatively spliced exons on homodimer and heterodimer assembly.," *Proc Natl Acad Sci U S A*, vol. 92, no. 21, pp. 9776–9780, Oct. 1995.

[15] M. Pascual, M. Vicente, L. Monferrer, and R. Artero, “The Muscleblind family of proteins: an emerging class of regulators of developmentally programmed alternative splicing,” *Differentiation*, vol. 74, no. 2, pp. 65–80, Mar. 2006, doi: 10.1111/j.1432-0436.2006.00060.x.

[16] Y. Yang *et al.*, “Determination of a Comprehensive Alternative Splicing Regulatory Network and Combinatorial Regulation by Key Factors during the Epithelial-to-Mesenchymal Transition,” *Molecular and Cellular Biology*, vol. 36, no. 11, pp. 1704–1719, Jun. 2016, doi: 10.1128/MCB.00019-16.

[17] D. Ray *et al.*, “A compendium of RNA-binding motifs for decoding gene regulation,” *Nature*, vol. 499, no. 7457, pp. 172–177, 2013, doi: 10.1038/nature12311.
